# Novel Planar Pt(II) Cyclometallated Cytotoxic Complexes with G-Quadruplex Stabilisation and Luminescent Properties

**DOI:** 10.3390/ijms231810469

**Published:** 2022-09-09

**Authors:** Brondwyn S. McGhie, Jennette Sakoff, Jayne Gilbert, Christopher P. Gordon, Janice R. Aldrich-Wright

**Affiliations:** 1 School of Science, Western Sydney University, Locked Bag 1797 Penrith South DC, Penrith, NSW 2751, Australia; 2 Calvary Mater Newcastle, Waratah, Newcastle, NSW 2298, Australia

**Keywords:** cyclometallated, G-quadruplex, Pt(II), anticancer

## Abstract

Herein is described the development of a series of novel quadruplex DNA (QDNA)-stabilising cyclometallated square–planar metal complexes (CMCs). Melting experiments using quadruplex DNA (QDNA) demonstrated that interactions with the complexes increased the melting temperature by up to 19 °C. This QDNA stabilisation was determined in two of the major G-quadruplex structures formed in the human c-MYC promoter gene (c-MYC) and a human telomeric repeat sequence (H-Telo). The CMCs were found to stabilise H-telo more strongly than c-MYC, and the CMCs with the highest cytotoxic effect had a low–moderate correlation between H-telo binding capacity and cytotoxicity (R^2^ values up to 10 times those of c-MYC). The melting experiments further revealed that the stabilisation effect was altered depending on whether the CMC was introduced before or after the formation of QDNA. All CMCs’ GI_50_ values were comparable or better than cisplatin in human cancer cell lines HT29, U87, MCF-7, H460, A431, Du145, BE2-C, SJ-G2, MIA, and ADDP. Complexes **6**, **7,** and **9** were significantly more cytotoxic than cisplatin in all cell lines tested and had good to moderate selectivity indices, 1.7–4.5 in MCF10A/MCF-7. The emission quantum yields were determined to be relatively high (up to 0.064), and emission occurred outside cellular autofluorescence, meaning CMC fluorescence is ideal for in vitro analyses.

## 1. Introduction

Cancer is increasingly becoming the leading cause of premature death worldwide, ranking first in 57 countries whilst placing either second or third in an additional 78 countries [1,2]. In 2020, cancer was the cause of almost 10 million deaths worldwide, and we can only expect this number to rise as cancer, more than likely, becomes the number one cause of death in most countries by the end of the century [1,2,3]. There is a clear and increasing need for efficient cancer treatments, yet current cancer therapy options remain limited, with low success rates for many types of cancers, particularly those in the later stages of the disease [4,5,6].

As an emerging alternative chemotherapeutic target, G-quadruplex DNA (QDNA) is attracting the interest of cancer scientists [7,8,9]. QDNA is an atypical DNA structure that is present in the telomeric region of chromosomes in which guanines undergo Hoogsteen pairing to form planar quartets rather than a double helix [9,10,11]. In many types of cancer, tumour cells survive through the extension of the telomeric region of their DNA using the telomerase enzyme. The binding and stabilisation of QDNA has been shown to prevent the action of telomerase, which can result in cell death [9,10,11].

Improving metal-based chemotherapy drugs remains a highly active area of cancer research [12,13,14,15]. Current clinically employed complexes that include cisplatin, oxaliplatin, and carboplatin are sub-optimal, and researchers continue to aspire to develop next-generation platinum metal complexes (MCs) with increased efficacy that target cancer cells to achieve better patient prognoses. Our research team has previously reported several Pt(II) complexes, with the structure [Pt(P_L_)_2_]^+2^, with specific binding affinity to QDNA [16]. The work presented here details the synthesis of cyclometallated complexes (CMCs), which bind QDNA and exhibit fluorescent properties. It is hypothesised that planar CMCs can and will stack with the base pairs at each end of the QDNA, thereby stabilising the quadruplex structure [17], preventing transcription, and ultimately leading to cell death. 

**Figure 1:**
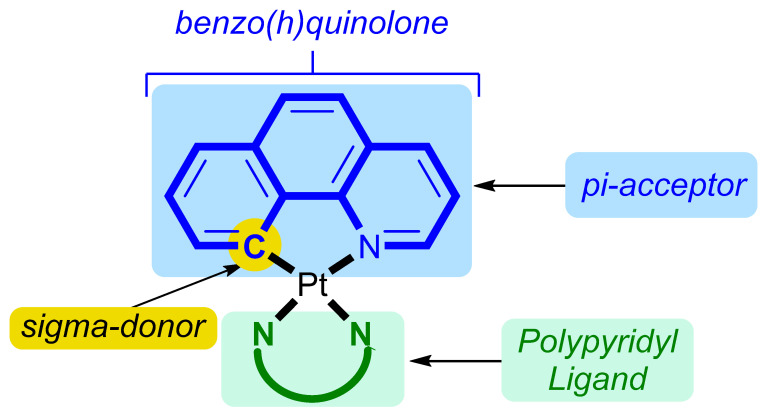
General structure of cyclometallated ligand benzo(h)quinoline CMCs.

The structural design of the complexes is based on previously unpublished developments from our group and comparable ruthenium(II) complexes studied by other groups, including 1,10-phenanthroline [18]. For these square–planar complexes, the cyclometallated ligand benzo(h)quinoline replaces 1,10-phenanthroline, chosen for its large surface area and flat aromatic structure and because it introduces fluorescence. Platinum coordinates through C1, forming an organo-metallic bond that results in the deprotonation of carbon, making it an effective σ-donor, whilst the polypyridyl group is a good π-acceptor creating a strong ligand field (Figure 1). This increase in the energy gap between the unoccupied and occupied orbitals caused by the σ-bond results in fluorescence. The fluorescence of the complexes may provide a means for intracellular tracking, hence enabling further mechanism-of-action studies to be performed [19,20,21,22].

**Figure 2:**
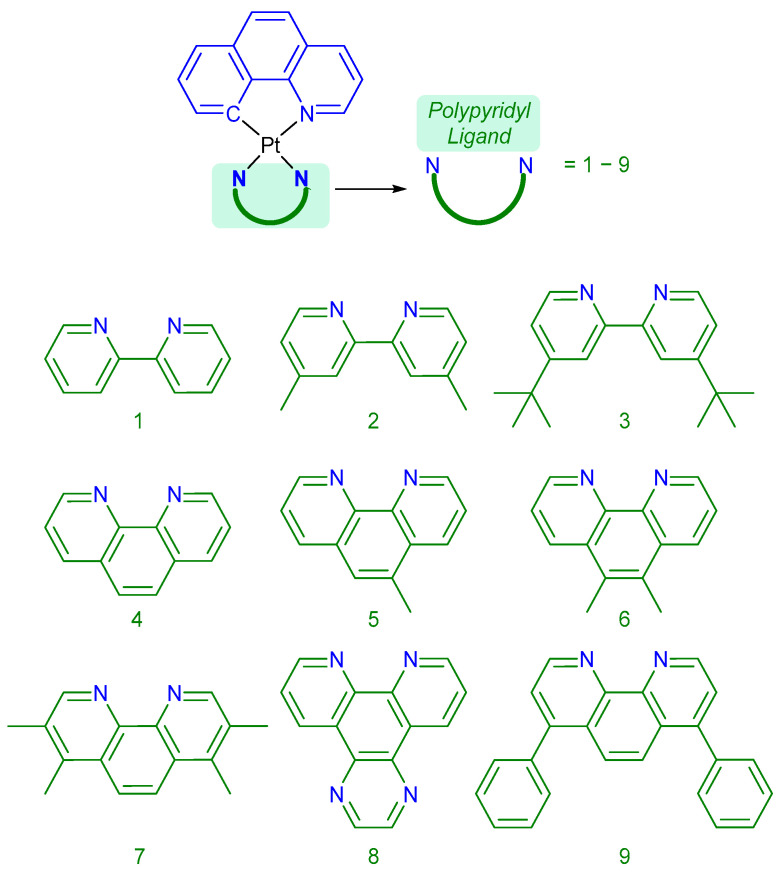
Structure of polyromantic ligands of all CMCs, coordinated as shown in the general structure outlined in Figure 1 to achieve: **1** [Pt(Bequ)(BPY)]^+^, **2** [Pt(Bequ)(44BPY)]^+^, **3** [Pt(Bequ)(tertBPY)]^+^, **4** [Pt(Bequ)(PHEN)]^+^, **5** [Pt(Bequ)(5MePHEN)]^+^, **6** [Pt(Bequ)(56Me_2_PHEN)]^+^, **7** [Pt(Bequ)(TMP)]^+^, **8** [Pt(Bequ)(DPQ)]^+^ and, **9** [Pt(Bequ)(BathoPHEN)]^+^. All complexes have chloride counterions.

A variety of polyaromatic ligands were chosen to compliment the cyclometallated ligand, with phenanthroline/bipyridine structures similar to those of the cyclometallated ligand beno[h]quinoline. We selected a variety of sizes and included ligands that have previously been observed to have anticancer and quadruplex-stabilising activities within our group [16]. Polyaromatic ligands provide a broad spectrum of lipophilicity and size from which to optimise cytotoxicity, fluorescence, and QDNA stabilisation (Figure 2). Accordingly, we report on the nine square–planar cyclometallated complexes (**1**–**9**) that were synthesised under microwave conditions affording high yields with high purity. Emission quantum yields were used to determine the innate fluorescence. While many techniques can be used to study the interactions of CMCs with QDNA, in this work, the stabilisation of QDNA as a result of interactions with CMCs was ascertained using UV DNA melting experiments, while the florescence was determined using the quantum yield.

## 2. Experimental Section

### 2.1. Materials and Methods

Reagents were used as received unless otherwise specified. All solvents used were of analytical grade or higher and purchased from Labserv, Chem-Supply, or Merck chemicals. Potassium tetrachloroplatinate (K_2_PtCl_4_) was purchased from Precious Metals Online. The chloride salts of platinum(II) complexes were synthesised using novel methods. Benzo(h)quinoline (Bequ), 2,2-bipyridine (BPY), 4,4′-dimethyl-2,2′-dipyridyl (44BPY), 4,4′-di-tert-butyl-2,2′-dipyridyl (tertBPY), 1,10-phenanthroline (PHEN), 5-methyl-1,10-phenanthroline (5MePHEN), 5,6-dimethyl-1,10-phenanthroline (56MePHEN), 3,4,7,8-tetramethyl-1,10-phenanthroline (TMP), pyrazino [2,3*f*][1,10]phenanthroline (DPQ), and batho(h)phenanthroline (BathoPHEN) were purchased from Sigma–Aldrich. Methanol, ethanol, diethyether, and methoxyethanol were obtained from Honeywell. Deuterated solvents d_6_-dimethylsulphoxide (DMSO-d_6_; 99.9%) and deuterium oxide (D_2_O; 99.9%) were purchased from Cambridge Isotope Laboratories. Oligonucleotides were purchased from Eurogentec. Microwave reactions took place in Biotage Initiator Robot 8.

### 2.2. Synthesis

#### 2.2.1. Synthesis of [Pt(Bequ)(Cl)_2_]^−^

Potassium tetrachloroplatinate (242.83 mg: 585.00 mmol: 1 equiv.) was dissolved in a 3:1 solution of water and methoxyethanol. Benzo(h)quinoline (263.65 mg: 1.471 mol: 2.5 equiv.) was subsequently added to the solution before the solution was heated to 80 °C and maintained for 16 h whilst stirring. After 16 h, the insoluble [Pt(Bequ)(Cl)_2_]^−^ had been synthesised and was filtered from the reaction solution and washed with diethyether (3 mL).

#### 2.2.2. Synthesis of [Pt(Bequ)(P_L_)]^+^ Complexes

[Pt(Bequ)(Cl)_2_]^−^ (1 equivalent) and the polypyridyl ligand (1.1 equivalents) were weighed into a 10 mL microwave tube with H_2_O (7 mL) and a magnetic stirring bar. Nitrogen or argon gas was bubbled through for ten minutes before the tube was quickly sealed with the microwave cap. The tube was then heated in the microwave (Biotage Initiator Robot 8 or CEM discover) to 175 °C for 1 h with the maximum Watts set at 100. The solution was allowed to cool to room temperature before it was transferred into a beaker (250 mL). To precipitate the final product, a mixture of methanol and diethyl ether (1:9) was added, starting with 50 mL and adding up to 200 mL in 50 mL aliquots until the complex precipitated. The solution was filtered, and the precipitate was washed and dried with diethylether (3 mL). This method was utilised for all nine complexes.

### 2.3. Cytotoxicity Methodology

Cytotoxicity assay studies were performed at Calvary Mater Newcastle Hospital, Waratah, NSW Australia. In vitro studies were performed according to described methods [23]. Complexes were prepared in DMSO as stock treatment (30 mM) solutions and stored at −20 °C. All cell lines were cultured in a humidified atmosphere with 5% CO_2_ at 37 °C and maintained in Dulbecco’s modified Eagle’s medium (DMEM; Trace Biosciences, Australia) supplemented with 10% foetal bovine serum, sodium bicarbonate (10 mM), penicillin (100 IU mL^−1^), streptomycin (100 μg mL^−1^), and L-glutamine (4 mM). The non-cancer MCF10A cell line was cultured in DMEM.F12 (1:1) cell culture medium (Composition in SI). Cytotoxicity was determined by plating cells in duplicate in 100 μL of medium at a density of 2500–4000 cells per well in 96-well plates. After 24 h, when cells were in logarithmic growth, medium (100 μL) with or without the test agent was added to each well (day 0). After 72 h of exposure, growth inhibitory effects were evaluated using the MTT (3-[4,5-dimethylthiazol-2-yl]-2,5-diphenyltetrazolium bromide) assay, and absorbance was read at 540 nm. An eight-point dose–response curve was produced, from which the drug concentration at which cell growth was inhibited by 50% (GI_50_) was calculated. These calculations were based on the difference between the optical density values on day 0 and those at the end of drug exposure.

### 2.4. Biophysical Characterisation

NMR spectral data were obtained using a 400 MHz Bruker Avance spectrometer at 298 K, using 10 mm samples prepared in D_2_O. ^1^H NMR spectra were obtained using a spectral width of 8250 Hz and 65,536 data points, while ^195^Pt NMR spectra were acquired using a spectral width of 85,470 Hz and 674 data points. ^1^H-^195^Pt HMQC spectra were recorded using a spectral width of 214,436 Hz and 256 data points for the ^195^Pt nucleus (F1 dimension) and a spectral width of 4808 Hz with 2048 data points for the ^1^H nucleus (F2 dimension).

UV spectra were recorded on a Cary 3500 UV-Vis spectrophotometer at room temperature in the 200–400 nm range, using a 10 mm quartz cell. All samples were automatically corrected for solvent baseline. The titration of a stock solution into a known volume of solvent in triplicate allowed the calculation of the molar absorption coefficient.

HPLC chromatograms were acquired on an Agilent Technologies 1260 Infinity machine equipped with a Phenomenex Onyx™ Monolithic C18 reverse-phase column (100 × 4.6 mm, 130 Å). The mobile phase comprised 0.06% TFA in water (solvent A) and 0.06% TFA in ACN:H_2_O (90:10; solvent B). Complexes were dissolved in water and flowed at a 0–100 gradient over 15 min, with an additional 15 min flush between each sample.

Electrospray ionisation mass spectroscopy (ESI-MS) experiments were performed using a Waters TQ-MS triple quadrupole mass spectrometer in positive mode. Sample solutions were made up to 0.5 mM in H_2_O and flowed at 0.1 mL/min. The desolvation temperature of 300 °C and desolvation flow rate (nitrogen) of 500 L/h remained consistent whilst the cone voltage and capillary voltage were varied for each sample to adjust for fragmentation. Spectra were collected over varied *m*/*z* ranges depending on the target mass.

Luminescence quantum yields were obtained using the comparison method, where [Ru(BPY)_3_]Cl_2_ was the standard chosen due to its similar excitation and emission maxima (Ex_max_ and Em_max_ respectively) [24]. Dilute solutions were prepared and titrated into water, and UV spectra were recorded, ensuring that they absorbed at no more than 0.1 abs at 310 nm after 5 titres. The solution was then titrated again into water, and the fluorescence emission, when excited at 310 nm, was recorded. This was repeated three times per sample; the results were then graphed to discover the final QY value.

Quadruplex-stabilisation studies were undertaken on a Cary 3500 UV-Vis spectrophotometer with a fully integrated air-cooled Peltier temperature control system. QDNA concentration was first checked using E_260_ (ds DNA; nn model) calculated using the ATDBio Ltd. online calculator. Then, stock solutions of oligonucleotides were made and annealed both in the presence and absence of the metal complexes at 0–6 equivalents of QDNA. Spectra were measured at every degree between 20 and 90 °C, with the temperature increasing at a rate of 0.5 °C min^−1^. Each melt was performed in triplicate, and the raw data were normalised before a sigmoidal model was fit to the curve to determine the melting temperature. The average melting temperature was then determined and tabulated.

The surface area of each complex was calculated using Vega ZZ x64 version with structures first refined using Avogadro 1.2.0 version.

Lipophilicity was calculated using an RP-HPLC method to determine log Kw, whereby a stock solution was injected at different isocratic ratios ranging from 70 to 90% solvent B (organic) at a flow rate of 1 mL min^−1^. These experiments were undertaken on an Agilent Technologies 1260 Infinity machine equipped with a Phenomenex Onyx™ Monolithic C18 reverse-phase column (100 × 4.6 mm, 130 Å). The mobile phase comprised 0.06% TFA in water (solvent A) and 0.06% TFA in ACN:H_2_O (90:10; solvent B). The dead-volume time was determined using potassium iodide as an external dead-volume marker (further details can be found in the Supplementary Materials).

## 3. Results and Discussion

### 3.1. Synthesis and Characterisation

**Scheme 1:**
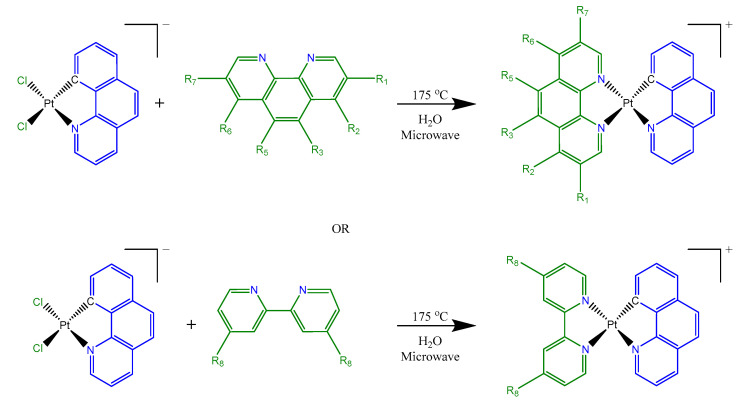
Synthesis of polyaromatic cyclometallated complexes (CMC): 1 if R_8_ = H ([Pt(Bequ)(BPY)]^+^), 2 if R_8_ = CH_3_ ([Pt(Bequ)(44BPY)]^+^), 3 if R_8_ = tertbutyl ([Pt(Bequ)(TertBPY)]^+^), 4 if R_1–7_ = H ([Pt(Bequ)(PHEN)]^+^), 5 if R_1,2,5–7_ = H and R_3_ = CH_3_ ([Pt(Bequ)(PHEN)]^+^), 6 if R_1,2,6,7_ = H and R_3,5_ = CH_3_ ([Pt(Bequ)(56Me_2_PHEN)]^+^, 7 if R_1,2,6,7_ = CH_3_ and R_3,5_ =H ([Pt(Bequ)(TMP)]^+^), 8 if the polypyridyl ligand was pyrazino[2,3-f][1,10]phenanthroline ([Pt(Bequ)(DPQ)]^+^) and, 9 if the polypyridyl ligand was batho(h)phenanthroline ([Pt(Bequ)(BathoPHEN)]^+^).

The synthesis of [Pt(benzo(h)quinoline)(Cl)_2_]^−^ ([Pt(Bequ)(Cl)_2_]^−^) was achieved through a novel method developed using microwave irritation. Initially, a reflux method was used; however, the reagents tended to degrade in the solution before a high yield could be achieved. This was most likely due to the slow reaction time at this temperature (48–72 h), which was solved using a microwave, which could bring the solution to 150 °C in 20 s, and the reaction was completed within an hour. Except for varying the solvent for the induction of final precipitation, the [Pt(Bequ)(P_L_)]^+^ complexes were afforded through the afore-described procedure (Scheme 1), where the counter ions were the chloride ions retained from the initial starting material, K_2_PtCl_4_. N_2_ gas was bubbled through the solution prior to microwave irritation; this was necessary for the safe use of the microwave and not for the sake of the reaction. We tested a reflux method under laboratory conditions, and although the yield was low, we believe this was due to the poor solubility of the products rather than the presence of oxygen.

### 3.2. Biophysical Characterisation

Each complex was characterised using a combination of NMR spectroscopy, HPLC, UV spectroscopy, ESI-MS, fluorescence, and MTT assays, as summarised in Table 1 and Table 2. NMR characterisation was achieved using a combination of ^1^H proton NMR spectra and ^1^H-^195^Pt heteronuclear multiple quantum correlation (HMQC) spectra. The NMR spectra produced peaks consistent with those seen in the literature for similar compounds, with little to no impurities being detected [18]. All platinum complexes were dissolved in deuterated dimethyl sulfoxide (450 µL) to obtain representative spectra, and the resonances characteristic of each complex were assigned (Supplementary Figures S2–S10).

**Figure 3:**
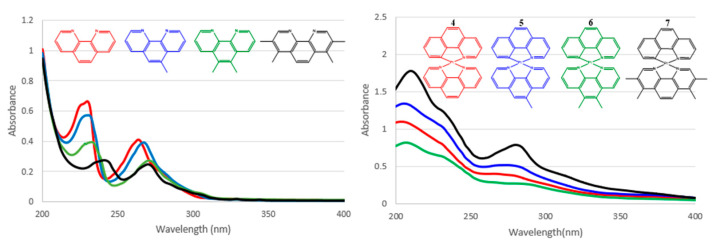
UV spectra of PHEN (red), 5MePHEN (blue), 56MePHEN (green), and TMP (black) at the same concentration in ethanol (**Left**) and UV spectra of **4** (red), **5** (blue), **6** (green), and **7** (black) at the same concentration in water (**Right**).

The UV absorption spectra of complexes **1**–**9** demonstrated that these complexes were predominantly absorbent between 200 and250 nm (π–π* between 200 and 250 nm; metal-to-ligand charge transfer (MLCT), 250–100 nm). The molar absorption coefficient (ε) was then calculated (Supplementary Figures S18–S26) for each individual complex that was obtained via the titration of a stock solution (10 µL) into a known volume of water (500 µL). The molar absorption coefficients of each ligand were calculated using the afore-described method using ethanol rather than water due to solubility (Supplementary Table S3). Although the UV data for ligands were obtained using ethanol and the data for complexes were obtained using water, the ligands’ spectra allowed us to distinguish different peaks in the complex spectra, which had relatively broad peaks with multiple shouldering (Supplementary Figures S11–S16). Distinct differences between the BPY coordinated complexes **1**–**3**; the PHEN derived complexes **4**–**7**; and large heteroaromatic complexes **8**–**9** were produced as expected based on the UV spectra of the ligands alone (Supplementary Figures S11–S14). Complexes **1**–**3** had low absorption from 400 to 250 nm (small peaks/shoulders due to d–d transitions) before increasing slowly to form an MLCT shoulder between 230 and 240 nm before rising to a peak between 205 and 210 nm (π–π*). In comparison, the absorbance of PHEN complexes **4**–**7** showed red-shifted absorbance with a small MLCT/d–d peak at ~270 nm before dropping again at 250 nm, with the π–π* peak being in the same 205–210 nm range as the BPY complexes. For complexes **4**–**8** and the corresponding PHEN ligands, the two peaks increasingly red-shifted as methylation increased (Figure 3).

**Figure 4:**
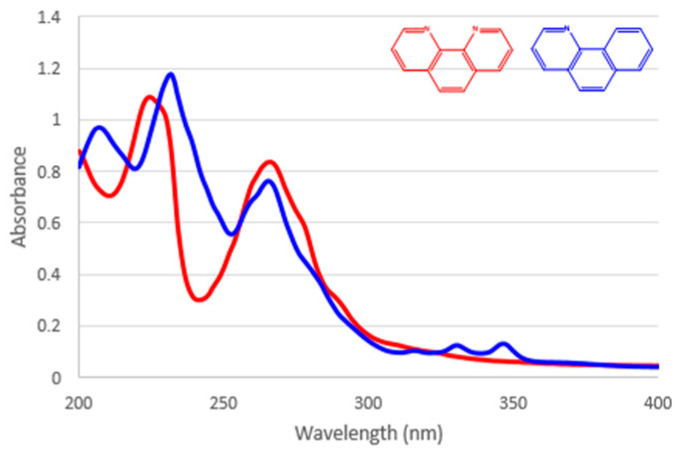
UV spectra of PHEN (red) and Bequ (blue) at the same concentration in ethanol and water solution.

With the exception of the MLCT/d–d small peak displaying an increased red shift to 230 nm and the large π–π* peak towards blue, complex **9** displayed a similar spectrum to those acquired for **4**–**7**. Complex **8** had the most distinct spectra with a large shoulder at 254 nm (whereas other complexes produced a trough in this region) and peaked at 206 nm. The absorbance of this complex was consistently high between 320 and 200 nm. Comparing the spectra, cyclometallated complex **4** ([Pt(bequ)(Phen)]^+^) and [Pt(Phen)_2_]^2+^, where both coordinated ligands are N^N, displayed similar spectra; however, the largest peak was blue-shifted (206 compared with 227 nm) and red-shifted (293 nm compared with 277 nm). A trough at 250 nm was more pronounced, and the peaks were less broad for the N^N complex, demonstrating that the loss of symmetry that resulted from the coordination of the cyclometallated C^N ligand impacted on absorbance properties similarly to the differences seen between the two absorbance spectra of the ligands (PHEN vs. Bequ) alone (Figure 4).

The radiative quantum yield was measured using [Ru(BPY)_3_]^2+^ as the standard. Samples were measured in water in triplicate, and Φem was calculated according to published methods [24] (see Supplementary Equation (S3)). Φ_em_ was relatively high, making them good luminescent complexes for use in cells; however, the excitation and emission wavelengths were outside the optic window of tissue, meaning their fluorescence properties are limited to in vitro studies. In future works, we hope to refine our understanding of the mechanism of action using their fluorescence, for example, in co-localisation studies.

Turning to an assessment of Log Kw (the capacity factor of the compound in 100% water) (Supplementary Equations (S4) and (S5)), no observable trends could be determined between the complexes’ volume, surface area, or length and lipophilicity until we divided them into three representative groups. These were the “BPY” complexes (**1**–**3**), the “PHEN” complexes (**4**–**6**), and the “Large” complexes (**7**–**9**). However, a positive correlation between lipophilicity and the three measurements of size with R^2^ values upwards of 0.97 were observable. Interestingly, the size of the “PHEN” complexes had no observable effects on lipophilicity, with R^2^ values in the range of 0.04–0.08. The largest complexes were the most unusual, in that there seemed to be a mild correlation with the complexes’ volume (R^2^ 0.7), a tenuous relationship with the complexes’ surface area (R^2^ 0.4), and no relationships with the complexes’ length (R^2^ 0.04) (Supplementary Tables S5–S8).

The stabilisation of QDNA was undertaken, with the DNA being annealed both in the presence of CMCs and without the metal complex, added just before the melt (as indicated by w/o CMCs and with CMCs, respectively). Seven samples were prepared for each of the two oligonucleotides with 0, 1, 2, 3, 4, 5, and 6 equivalents of CMCs in triplicate for each of the nine CMCs. The two sequences selected were the major G-quadruplex structures formed in human c-MYC promoter gene PDB reference 1XAV (TGA GGG T GGG TA GGG T GGG TAA) [25,26] and a human telomeric repeat sequence, d[AG_3_(T_2_AG_3_)_3_] PDB reference 143D (AGGG TTA GGG TTA GGG TTA GGG) [27]. The 22mer parallel structures are abbreviated as c-MYC and H-telo, respectively. Both oligonucleotides were stabilised in the presence of all nine CMCs, with stabilisation increasing as the ratio of CMCs increased.

**Figure 5:**
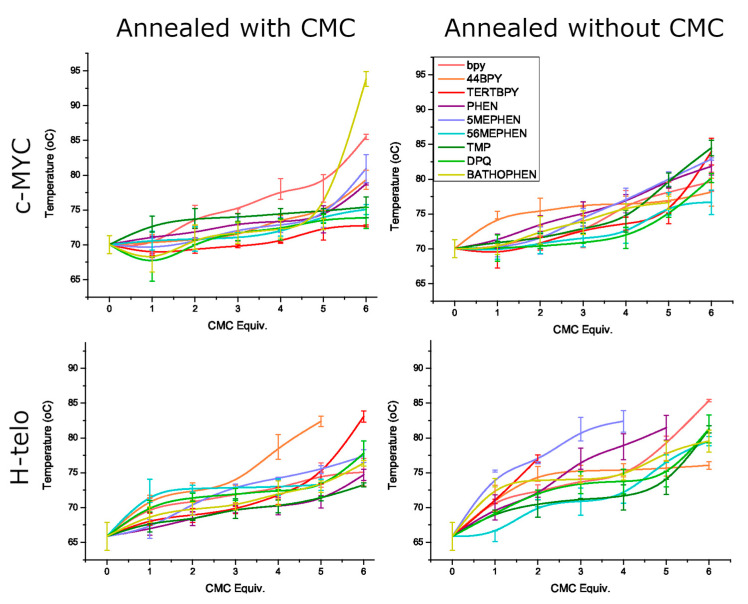
ΔTM of c-MYC and H-telo per equivalent of CMCs when annealed with vs. without CMCs.

There were considerable differences between the melting temperature of QDNA annealed in the presence of CMCs and in their absence, and a more apparent trend was observed for the c-MYC sequence, which appeared to be destabilised by the presence of CMCs upon annealing (Figure 5). This trend suggests that 1 equivalent of a CMC initially disrupted the stability of QDNA during annealing. The addition of subsequent equivalents stabilised the structure, and we saw the overall stabilisation up to 6 equivalents. It was anticipated that the melting temperature would have plateaued after a certain number of equivalents of CMCs, as all the potential binding sites were filled; however, this was not observed under either of the reaction conditions. For example, for c-MYC annealed with CMCs, the melting temperature plateaued beyond 2 equivalents remained relatively stable up to 5 equivalents before spiking at 6 equivalents, whereas for c-MYC annealed in the absence of CMCs, the melting temperature continued to steadily increase with additional equivalents of CMCs (Figure 5).

The change in the melting temperature (ΔTM) was plotted against lipophilicity, molecular weight, molecular length, molecular volume, surface area, and GI_50_ values to determine if any correlations between these CMC properties and the overall stabilisation of QDNA could be observed (Supplementary Tables S2–S4) for the three groups: the “BPY” complexes (**1**–**3**), the “PHEN” complexes (**4**–**6**), and the “Large” complexes (**7**–**9**). For the BPY group size complexes’ length, volume, and surface area, each correlated with c-MYC stabilisation (R^2^ upwards of 0.98) but less so for H-Telo (R^2^ between 0.4 and 0.7). This trend was not observed for either of the other two groups, yet stabilisation for both c-MYC and H-Telo somewhat correlated with CMC lipophilicity. ΔTM did not seem to directly influence the GI_50_ values, although it was interesting to note that for both the “PHEN” and “BPY” groups, c-MYC stabilisation related to the GI_50_ values, whereas for the “Large” group, H-Telo stabilisation had more of a relationship. These results suggested that the overall size impacted the capacity to stabilise QDNA, with the largest aromatic CMC, **8**, demonstrating the most stabilising impact on QDNA; however, these complexes can induce their cytotoxic effect by other means. Alternatively, the absence of observable correlations could be due to the difference in the occurrence of QDNA between each cell line, and different CMCs stabilised said QDNA with trends different from those of c-MYC and H-telo. Alternatively, there lies the possibility for a CMC to be introduced at various stages of the cell cycle. Thus, in some instances, we could see annealing with CMC effects and in others w/o CMC effects; this could also relate to under what conditions for each cell line and for how long during each cell cycle QDNA is present.

Regarding GI_50_ values against a panel of cell lines, the CMCs displayed activity comparable with that of cisplatin (Table 2). Although, at first glance, it may be concluded that the overall size of the CMC is the determining factor for cytotoxicity, a closer examination shows otherwise. In order to make sense of the data, we grouped the complexes based on their N^N ligand, “BPY”, “PHEN”, and “Large”, after which trends appeared. For the “BPY” CMCs, there were no consistent correlations among the complexes’ length, volume, surface area, or lipophilicity. The “PHEN” complexes had strong correlations between size indicators and cytotoxicity (R^2^ upwards of 0.95 for most conditions) but not at all for lipophilicity (R^2^ less than 0.04). Almost the reverse was apparent for the “Large” complexes, with lipophilicity being the strongest predictor of cytotoxicity (R^2^ upwards of 0.98) and with only mild to weak correlations to volume and surface area but no relationship at all with complex length (which made sense, as bathophenanthroline added aromatic bulk rather than length). Clearly, further investigation into their mechanism of action is required to understand their cytotoxicity. Further derivatisation and fluorescence in vitro studies are planned in future work to elucidate the structure–activity relationship and cytotoxic mechanism.

## 4. Conclusions

Nine novel cyclometallated metal complexes (CMCs) were synthesised in good yield and purity and determined to have good photoluminescent and cytotoxic properties. All CMCs showed strong evidence of QDNA stabilisation using melting experiments for two of the major G-quadruplex structures formed in humans, the c-MYC promoter gene and a human telomeric repeat sequence (H-Telo). QDNA stabilisation was achieved both when a CMC was present and when it was absent at the point of annealing; this could mean that the QDNA binding capacity would not be negated if entering the cell during the phases of the cell cycle at which QDNA is yet to form. The GI_50_ values in human cancer cell lines were promising, with all CMCs having enhanced activity relative to cisplatin in some cell lines and approximately equivalent in others. At the same time, complexes **6**, **7**, and **9** displayed significantly improved cytotoxicity compared with cisplatin in all cell lines tested with moderate selectivity for cancer (selectivity index of 4.5). QDNA stabilisation and cytotoxicity could not be directly correlated. However, some trends were apparent between the stabilisation of either c-MYC of H-Telo and cytotoxicity, which suggests to us that either QDNA is not the cellular target or that the presence of QDNA is inconsistent among cell lines and has stabilisation factors different from the examples of QDNA tested. Future work is to focus on two main goals, broadening the range of structures to tease out the structure–activity relationship and utilising the fluorescence of the complexes to ascertain the mechanism of action, for example, by tracking their accumulation within cells using fluorescent co-localisation studies. We believe that these novel complexes provide a new base complex to be fine-tuned, with their strong photoluminescence providing an advantage over other platinum(II) complexes currently being investigated for cancer therapy.

## 5. Patents

The complexes described in this manuscript are included in Western Sydney University 2022 “Platinum (IV) complexes” 197461PRV.

## Figures and Tables

**Table 1:** Summary of biophysical characterisation of all nine complexes. The correct mass peak was to be identified for each complex using ESI-MS, and no major substructures were seen, suggesting that these complexes are quite stable and pure, which was further demonstrated with HPLC, with each complex achieving HPLC purity above 95% (Supplementary Figures S27–S44).

CMC	Molecular Formula	Yield(%)	ESI-MS (m/z)	Lipophilicity (log kw)	Surface Area VdW/SAS (Å²)	UV/λmax (nm) (ε/mol^−1^·dm^3^·cm^−1^) × 102	Fluorescence		
			[M]+ Calc. (Found)				Ex_max_	Em_max_	ΦF
**1**	C_23_H_16_N_3_Pt	84.05	529.0992 (529.0981)	1.04 ± 0.15	773.9	206 (330 ± 0.18), 233 (341 ± 0.19)	324.02	652.02	0.015 ± 0.121
**2**	C_25_H_20_N_3_Pt	86.02	557.1305 (557.1295)	1.63 ± 0.20	842.9	208 (401 ± 0.32), 232 (282 ± 0.33)	336.00	634.92	0.024 ± 0.175
**3**	C_32_H_31_N_3_Pt	83.59	657.2315 (657.2319)	2.35 ± 0.16	989.4	208 (315 ± 0.14), 232 (252 ± 0.16)	395.07	670.00	0.016 ± 0.187
**4**	C_25_H_16_N_3_Pt	82.49	553.0992 (553.0989)	1.12 ± 0.11	803.9	204 (442 ± 0.16), 265 (163 ± 0.15)	325.05	654.05	0.021 ± 0.236
**5**	C_26_H_18_N_3_Pt	86.23	567.1149 (567.1147)	1.30 ± 0.16	830.8	206 (402 ± 0.11), 274 (153 ± 0.19)	394.24	645.05	0.014 ± 0.222
**6**	C_27_H_20_N_3_Pt	85.56	581.1305 (581.1308)	1.04 ± 0.25	856.2	207 (398 ± 0.15), 284 (130 ± 0.12)	325.97	651.01	0.026 ± 0.155
**7**	C_29_H_24_N_3_Pt	82.89	609.1618 (609.1611)	2.62 ± 0.30	909.8	210 (816 ± 0.12), 280 (359 ± 0.18)	391.96	641.01	0.023 ± 0.136
**8**	C_27_H_16_N_5_Pt	78.65	605.1054 (605.1057)	1.13 ± 0.14	864.1	206 (263 ± 0.26), 254 (121 ± 0.21)	332.00	635.07	0.060 ± 0.172
**9**	C_37_H_24_N_3_Pt	79.89	705.1618 (705.1616)	2.73 ± 0.34	1117.4	229 (104 ± 0.19), 254 (495 ± 0.24)	258.00	699.00	0.064 ± 0.249

**Table 2:** GI_50_ data for each of the nine complexes compared to cisplatin.

Cell Line	Complex									
	1	2	3	4	5	6	7	8	9	Cisplatin
HT29	10 ± 2.3	13 ± 0.3	3.8 ± 0.2	11 ± 0.7	2.1 ± 0.1	2.1 ± 0.1	1.1 ± 0.3	4.2 ± 0.3	0.29 ± 0.01	nd
U87	12 ± 0.3	20 ± 0.9	3.9 ± 0.0	14 ± 0.3	2.5 ± 0.2	2.0 ± 0.1	0.38 ± 0.02	7.5 ± 0.8	0.33 ± 0.03	3.8 ± 1.1
H460	3.5 ± 0.3	12 ± 0.3	3.9 ± 0.4	3.8 ± 0.2	1.3 ± 0.03	0.67 ± 0.14	0.29 ± 0.03	3.5 ± 0.4	0.36 ± 0.03	0.9 ± 0.2
A431	3.8 ± 0.2	14 ± 0.3	5.7 ± 0.5	4.5 ± 0.5	2.2 ± 0.2	1.7 ± 0.2	0.42 ± 0.01	5.7 ± 0.6	0.36 ± 0.02	2.4 ± 0.3
Du145	3.2 ± 0.4	13 ± 0.9	4.4 ± 0.2	4.6 ± 0.7	1.8 ± 0.1	1.4 ± 0.4	0.32 ± 0.04	8.1 ± 1.2	0.57 ± 0.04	1.2 ± 0.1
BE2-C	2.3 ± 0.4	5.1 ± 1.2	2.0 ± 0.3	2.7 ± 0.3	1.1 ± 0.3	0.59 ± 0.28	0.17 ± 0.05	2.5 ± 0.4	0.22 ± 0.03	1.9 ± 0.2
SJ-G2	2.8 ± 0.1	12 ± 0.7	3.1 ± 0.03	3.1 ± 0.09	2.1 ± 0.1	1.4 ± 0.3	0.27 ± 0.01	3.1 ± 0.1	0.26 ± 0.00	0.4 ± 0.1
MIA	13.7 ± 0	19 ± 2	5.1 ± 0.97	14 ± 0	2.3 ± 0.3	2.3 ± 0.2	0.49 ± 0.10	12 ± 1	0.30 ± 0.01	7.5 ± 1.3
ADDP	4.3 ± 1.3	14 ± 2	3.5 ± 1.0	4.9 ± 1.6	1.7 ± 0.6	1.4 ± 0.6	0.49 ± 0.21	3.3 ± 0.9	0.30 ± 0.04	1.2 ± 0.1
MCF-7	2.1 ± 0.09	12 ± 0.3	2.4 ± 0.3	2.9 ± 0.32	1.9 ± 0.29	1.3 ± 0.12	0.82 ± 0.39	2.2 ± 0.2	0.22 ± 0.009	6.5 ± 0.8
MCF10A	3.6 ± 0.4	13 ± 0	7.5 ± 2.2	4.1 ± 0.7	2.6 ± 0.15	2.2 ± 0.38	0.50 ± 0.01	5.0 ± 0.5	1.00 ± 0.23	nd
SI	1.7	1.1	3.1	1.4	1.4	1.7	0.6	2.3	4.5	

SI = GI_50_ for normal (non-cancerous) cells/GI_50_ for cancer cells. SI value > 2 indicated high selectivity.

## Supplementary Materials

The following supporting information can be downloaded at: https://www.mdpi.com/article/10.3390/ijms231810469/s1.

## References

[1] Bray F., Laversanne M., Weiderpass E., Soerjomataram I. (2021). The Ever-Increasing Importance of Cancer as a Leading Cause of Premature Death Worldwide. Cancer.

[2] Sung H., Ferlay J., Siegel R., Laversanne M., Soerjomataram I., Jemal A., Bray F. (2021). Global Cancer Statistics 2020: GLOBOCAN Estimates of Incidence and Mortality Worldwide for 36 Cancers in 185 Countries. CA Cancer J. Clin..

[3] Cancer (IARC); T.I.A. for R. on Global Cancer Observatory. https://gco.iarc.fr/.

[4] Wheate N.J., Walker S., Craig G.E., Oun R. (2010). The Status of Platinum Anticancer Drugs in the Clinic and in Clinical Trials. Dalton Trans..

[5] Siddik Z.H. (2003). Cisplatin: Mode of Cytotoxic Action and Molecular Basis of Resistance. Oncogene.

[6] Kartalou M., Essigmann J.M. (2001). Mechanisms of Resistance to Cisplatin. Mutat. Res. Mol. Mech. Mutagen..

[7] Balasubramanian S., Hurley L.H., Neidle S. (2011). Targeting G-Quadruplexes in Gene Promoters: A Novel Anticancer Strategy?. Nat. Rev. Drug Discov..

[8] Ou T., Lu Y., Tan J., Huang Z., Wong K.-Y., Gu L. (2008). G-Quadruplexes: Targets in Anticancer Drug Design. ChemMedChem.

[9] Düchler M. (2012). G-Quadruplexes: Targets and Tools in Anticancer Drug Design. J. Drug Target..

[10] Monchaud D., Teulade-Fichou M.-P. (2008). A Hitchhiker’s Guide to G-Quadruplex Ligands. Org. Biomol. Chem..

[11] De Cian A., Cristofari G., Reichenbach P., De Lemos E., Monchaud D., Teulade-Fichou M.-P., Shin-ya K., Lacroix L., Lingner J., Mergny J.-L. (2007). Reevaluation of Telomerase Inhibition by Quadruplex Ligands and Their Mechanisms of Action. Proc. Natl. Acad. Sci. USA.

[12] Deo K.M., Ang D.L., McGhie B., Rajamanickam A., Dhiman A., Khoury A., Holland J., Bjelosevic A., Pages B., Gordon C. (2018). Platinum Coordination Compounds with Potent Anticancer Activity. Coord. Chem. Rev..

[13] McGhie B.S., Sakoff J., Gilbert J., Aldrich-Wright J.R. (2019). Synthesis and Characterisation of Platinum(IV) Polypyridyl Complexes with Halide Axial Ligands. Inorg. Chim. Acta.

[14] Khoury A., Deo K.M., Aldrich-Wright J.R. (2020). Recent Advances in Platinum-Based Chemotherapeutics That Exhibit Inhibitory and Targeted Mechanisms of Action. J. Inorg. Biochem..

[15] Deo K.M., Sakoff J., Gilbert J., Zhang Y., Wright J.R.A. (2019). Synthesis, Characterisation and Influence of Lipophilicity on Cellular Accumulation and Cytotoxicity of Unconventional Platinum(IV) Prodrugs as Potent Anticancer Agents. Dalton Trans..

[16] Ang D.L., Harper B.W.J., Cubo L., Mendoza O., Vilar R., Aldrich-Wright J. (2016). Quadruplex DNA-Stabilising Dinuclear Platinum(II) Terpyridine Complexes with Flexible Linkers. Chem.-Eur. J..

[17] Wilson T., Costa P.J., Félix V., Williamson M.P., Thomas J.A. Structural Studies on Dinuclear Ruthenium(II) Complexes That Bind Diastereoselectively to an Antiparallel Folded Human Telomere Sequence. https://pubs.acs.org/doi/full/10.1021/jm401119b.

[18] Holland J. (2017). Synthesis of Platinum(II) Based G-Quadruplex Stabilisers. Master’s Thesis.

[19] Tan C.-P., Zhong Y.-M., Ji L.-N., Mao Z.-W. (2021). Phosphorescent Metal Complexes as Theranostic Anticancer Agents: Combining Imaging and Therapy in a Single Molecule. Chem. Sci..

[20] Qiu K., Chen Y., Rees T.W., Ji L., Chao H. (2019). Organelle-Targeting Metal Complexes: From Molecular Design to Bio-Applications. Coord. Chem. Rev..

[21] Ding S., Qiao X., Suryadi J., Marrs G.S., Kucera G.L., Bierbach U. (2013). Using Fluorescent Post-Labeling To Probe the Subcellular Localization of DNA-Targeted Platinum Anticancer Agents. Angew. Chem. Int. Ed..

[22] Xu G.-X., Mak E.C.-L., Lo K.K.-W. (2021). Photofunctional Transition Metal Complexes as Cellular Probes, Bioimaging Reagents and Phototherapeutics. Inorg. Chem. Front..

[23] Tarleton M., Gilbert J., Robertson M.J., McCluskey A., Sakoff J.A. (2011). Library Synthesis and Cytotoxicity of a Family of 2-Phenylacrylonitriles and Discovery of an Estrogen Dependent Breast Cancer Lead Compound. MedChemComm.

[24] Brouwer A.M. (2011). Standards for Photoluminescence Quantum Yield Measurements in Solution (IUPAC Technical Report). Pure Appl. Chem..

[25] González V., Hurley L.H. (2010). The C-MYC NHE III(1): Function and Regulation. Annu. Rev. Pharmacol. Toxicol..

[26] Ambrus A., Chen D., Dai J., Jones R.A., Yang D. (2005). Solution Structure of the Biologically Relevant G-Quadruplex Element in the Human c-MYC Promoter. Implications for G-Quadruplex Stabilization. Biochemistry.

[27] Wang Y., Patel D.J. (1993). Solution Structure of the Human Telomeric Repeat d [AG3 (T2AG3) 3] G-Tetraplex. Structure.

