# Supplementary material for: Novel Planar Pt(II) Cyclometallated Cytotoxic Complexes with G-Quadruplex Stabilisation and Luminescent Properties
Source: Int J Mol Sci. 2022 Sep 9;23(18):10469. doi: 10.3390/ijms231810469 (PMC9499473; doi:10.3390/ijms231810469)
Supplement: Supplementary file 1 [file ijms-23-10469-s001.zip › ijms-1807690-supplementary.pdf]

Novel Planar Pt(II) Cyclometallated Cytotoxic Complexes with G-Quadraplex Stabilisation and Luminescent Properties

Brondwyn S. McGhie,[a] Jennette Sakoff,[b] Jayne Gilbert,[b] Christopher P. Gordon[a] and Janice R. Aldrich-Wright[a*]

[a] *School of Science, Nanoscale Organisation and Dynamics Group, Western Sydney University, Locked Bag 1797 Penrith South DC, 2751, NSW, Australia*

[b] *Calvary Mater Newcastle, Waratah, NSW 2298, Australia*

**Corresponding author – Email. J.Aldrich-Wright@westernsydney.edu.au, Ph. +61246203218*

Supplementary Data

Characterisation 3
- NMR 3
  - [Pt(Bequ)(PHEN)]+ 5
  - [Pt(Bequ)(BPY)]+ 7
  - [Pt(Bequ)(44BPY)]+ 8
  - [Pt(Bequ)(TertBPY)]+ 9
  - [Pt(Bequ)(5MePHEN)]+ 10
  - [Pt(Bequ)(56MePHEN)]+ 11
  - [Pt(Bequ)(TMP)]+ 12
  - [Pt(Bequ)(DPQ)]+ 13
  - [Pt(Bequ)(BathoPHEN)]+ 14
- UV Spectra 15
  - Ligands 15
  - Complexes 17

Molar Absorption Coefficients 19
  - [Pt(Bequ)(BPY)]+ 19
  - [Pt(Bequ)(44BPY)]+ 20
  - [Pt(Bequ)(TertBPY)]+ 20
  - [Pt(Bequ)(PHEN)]+ 21
  - [Pt(Bequ)(5MePHEN)]+ 21
  - [Pt(Bequ)(56MePHEN)]+ 22
  - [Pt(Bequ)(TMP)]+ 22
  - [Pt(Bequ)(DPQ)]+ 23
  - [Pt(Bequ)(BathoPHEN)]+ 23
- HPLC 24
  - [Pt(Bequ)(BPY)]+ 24
  - [Pt(Bequ)(44BPY)]+ 24
  - [Pt(Bequ)(TertBPY)]+ 25
  - [Pt(Bequ)(PHEN)]+ 25

[Pt(Bequ)(5MePHEN)]$^{+}$ 26
[Pt(Bequ)(56MePHEN)]$^{+}$ 26
[Pt(Bequ)(TMP)]$^{+}$ 27
[Pt(Bequ)(DPQ)]$^{+}$ 27
[Pt(Bequ)(BathoPHEN)]$^{+}$ 28
ESI MS 29
[Pt(Bequ)(BPY)]$^{+}$ 29
[Pt(Bequ)(44BPY)]$^{+}$ 30
[Pt(Bequ)(TertBPY)]$^{+}$ 31
[Pt(Bequ)(PHEN)]$^{+}$ 32
[Pt(Bequ)(5MePHEN)]$^{+}$ 33
[Pt(Bequ)(56MePHEN)]$^{+}$ 34
[Pt(Bequ)(TMP)]$^{+}$ 35
[Pt(Bequ)(DPQ)]$^{+}$ 36
[Pt(Bequ)(BathoPHEN)]$^{+}$ 37
Fluorescence 38
QY 38
Fluorescent Titrations 38
[Pt(Bequ)(44BPY)]$^{+}$ 39
[Pt(Bequ)(TertBPY)]$^{+}$ 40
[Pt(Bequ)(PHEN)]$^{+}$ 41
[Pt(Bequ)(5MePHEN)]$^{+}$ 43
[Pt(Bequ)(56MePHEN)]$^{+}$ 44
[Pt(Bequ)(TMP)]$^{+}$ 45
[Pt(Bequ)(DPQ)]$^{+}$ 46
[Pt(Bequ)(BathoPHEN)]$^{+}$ 47
DNA Melts 48
Lipophilicity 49
Correlation analysis 50
GI_{50} 54

Characterisation

NMR

NMR Spectral data were obtained using a 400 MHz Bruker Avance spectrometer at 298 K, using 10 mm samples prepared in D_2O. ^{1}H NMR spectra were obtained using a spectral width of 8250 Hz and 65536 data points, while ^{195}Pt NMR spectra were acquired using a spectral width of 85470 Hz and 674 data points. ^{1}H-^{195}Pt HMQC spectra were recorded using a spectral width of 214436 Hz and 256 data points for the ^{195}Pt nucleus (F1 dimension) and a spectral width of 4808 Hz with 2048 data points for the ^{1}H nucleus (F2 dimension). Chemical shifts are reported in parts per million (ppm) with J coupling reported in Hz.

Figure S2 is an example of how the ^{1}H peaks were identified and assigned showing integration in red. We also utilizes the COSY of the Benzo[h]quinoline to help assign peaks Figure 2b.

Firstly, the peaks were integrated as shown in red, as we would expect the Phenanthroline peaks have a value of two as the ligand is symmetrical whereas the benzoquinoline ligand is asymmetric, meaning that each peak will have an integration of one. Next, we looked at the J coupling, the duplex peak assigned to P2/9 had a larger j coupling than duplex the peak assigned to the P4/7 confirming our assignment. We see that these peaks can be found further upstream or downstream to each other depending on the concentration of the sample which we believe is due to π- π stacking. Next, we identified the 5/6 peaks for both ligands, these could easily be identified in the COSY as only coupling to each other, and we see that the 5/6 peak for the asymmetric ligand is split unlike for the symmetric ligand. Next the 3/8 protons were assigned, we did this again using the cosy, they were identified as the protons who were coupling with two other peaks, (which we can thus identify is the 9/2 and 4/7 peaks). This left us to identify the remaining 2/9 peaks as those coupling to the corresponding B or P 3 and 8 peaks. This logic was followed for the remaining complexes. Where the assignment was less clean, we incorporated the knowledge gained from the HMQC which tells us which protons are closely coupled to the Pt centre. All proton assignments are summarised in the table below.

Proton	Complex								
	1	2	3	4	5	6	7	8	9
B2	δ 7.25(d, J= 4.95 Hz, H1)	δ 7.15 (d, J= 5.02 Hz, H1)	δ 8.38 (merged)	δ 8.39 (d, J= 7.55 Hz, H1)	δ 5.09 (H1)	δ 4.66 (H1)	δ9.69 (d, J= 5.04 Hz, H1)	δ 6.85 (d, J= 8.59 Hz, H1)	δ6.74 (d, J= 10.3 Hz, H1)
B3	δ 6.95 (d, J= 6.17 Hz, H1)	δ 7.03 (d, J= 8.06 Hz, H1)	δ 7.85 (t, J= 9.09 Hz, H1)	δ7.91 (merged, ~H1)	δ 6.16(H1)	δ 5.41 (H1)	δ 7.91 (t, J= 6.72 Hz, H1)	δ 8. 13 (d, J= 7.71 Hz, H1)	δ7.00(d, J= 4.22 Hz, H1)
B4	δ 8.47 (d, J= 5.65 Hz, H1)	δ 7.53 (d, J= 5.02 Hz, H1)	δ9.70 (d, J= 4.96 Hz, H1)	δ9.70 (d, J= 5.05 Hz, H1)	δ 7.36 (H1)	δ 6.69 (H1)	δ 8.39 (d, J= 7.56 Hz, H1)	δ 9.41 (d, J= 9.45 Hz, H1)	Δ9.15 (d, J= 6.76 Hz, H1)
B5	δ 6.62 (s, H2)	δ 6.00 (s,(merged ~H2))	δ 8.41 (s, H2)	δ 7.78 (merged, ~H2)	δ 5.39 (H1)	δ 4.55 (H2)	δ 7.79 (d, J= 9.24 Hz, H2)	δ 7.09(s, H1)	δ6.82 (d, J= 10.3 Hz, H2)
B6								δ 7.02(s, H1)	
B7	δ 8.33 (d, J= 5.65 Hz, H1)	δ 7.37 (d, J= 5.02 Hz, H1)	δ 8.75 (d, J= 6.61 Hz, H1	δ 8.76(d, J= 5.08 Hz, H1)	δ 6.94 (H1)	δ 6.31 (H1)	δ 8.75 (d, J= 8.40 Hz, H1)	δ 9.03 (d, J= 8.59 Hz, H1)	δ8.17(d, J= 5.91 Hz, H1)
B8	δ 6.18 (d, J= 6.17 Hz, H1)	δ 6.31 (d, J= 8.05 Hz, H1)	δ 7.97 (t, J= 9.09 Hz, H1)	δ 7.90 (t, J= 6.06 Hz, H1)	δ 6.37 (H1)	δ 5.69 (H1)	δ 7.74 (t, J= 6.72 Hz, H1)	δ 8.41 (d, J= 7.71 Hz, H1)	Δ8.31(d, J= 4.22 Hz, H1)
B9	δ 7.25(d, J= 7.54 Hz, H1)	δ 5.73 (d, J= 5.02 Hz, H1)	δ 7.48 (d, J= 4.13 Hz, H1)	δ 8.39 (d, J= 7.07Hz, H1)	δ 5.44 (H1)	δ 4.83 (H1)	δ 8.57 (d, J= 8.40 Hz, H1)	δ 7.18 (d, J= 8.59 Hz, H1)	δ7.26 (d, J= 5.07 Hz, H1)
P2	δ 6.38 (d, J= 9.96 Hz, H1)	δ 6.53 (d, J= 5.54 Hz, H1)	δ 8.61 (d, J= 5.79 Hz, H2) (P2/9)	δ 8.51 (d, J= 8.08 Hz, H2) (P2/9)	δ 5.22 (H1)	δ 4.74 (H1)	δ7.86 (s, H1)	δ 6.94 (d, J= 10.31 Hz, H1)	δ6.90 (d, J= 10.13 Hz, H1)
P3	δ 6.76 (d, J= 6.33 Hz, H1)	δ 6.06(d, J= 7.54 Hz, H1)	δ7.90(d, J= 5.79 Hz, H1)	δ7.85 (d, J= 8.08 Hz, H1)	δ 6.66 (H1)	δ 5.94 (H1)	δ 2.75 (s, H4) (P3,4,7,8)	δ 8.81 (d, J= 8.59 Hz, H1)	δ 8.5(d, J= 5.07 Hz, H1)
P4	δ 7.51 (d, J= 6.91 Hz, H1)	δ 1.60 (s, H6) (P4/P7)		δ 9.11 (d, J= 3.03 Hz, H2) (P2/9)	δ 5.22 (H1)	δ 4.91 (H1)		δ 8.53 (d, J= 19.7 Hz, H1)	
P5	δ 5.99 (Merged)	δ 5.87 (s, H1)	δ 7.71 (s, H1)	δ 8.02 (s, H2)	δ 1.05 (s, H1)	δ 0.99 (s, H2)	δ 9.11 (d, J= 4.20, Hz H1)	δ 8.29 (s, H2)	δ7.32(d, J= 3.88 Hz, H2)
P6		δ 5.83 (s, H1)	δ 7.77 (s, H1)		δ 5.15 (H1)		δ 9.03 (d, J= 4.20, Hz H1)		
P7	δ 7.43 (d, J= 6.91 Hz, H1)	δ 1.60 (s, H6) (P4/P7)		δ 9.11 (d, J= 3.03 Hz, H2) (P2/9)	δ 5.11 (H1)	δ 4.73 (H1)	δ 2.75 (s, H4) (P3,4,7,8)	δ 7.13(d, J= 19.7 Hz, H1)	
P8	δ 6.87 (d, J= 6.93 Hz, H1)	δ 6.00 (merged ~H1)	δ7.55(d, J= 5.79 Hz, H1)	δ7.97 (d, J= 8.08 Hz, H1)	δ 6.00 (H1)			δ 7.57 (d, J= 8.59 Hz, H1)	δ7.63(d, J= 7.60 Hz, H1)
P9	δ 7.31 (d, J= 10.05 Hz, H1)	δ 6.68 (d, J= 5.54 Hz, H1)	δ 8.61 (d, J= 5.79 Hz, H2) (P2/9)	δ 8.51 (d, J= 8.08 Hz, H2) (P2/9)	δ 5.64 (H1)	δ 4.91 (H1)	δ7.96 (s, H1)	δ 7.34 (d, J= 10.31 Hz, H1)	δ7.11(d, J= 6.76 Hz, H1)
T1,T2,T3			δ 1.35 (s, H18)						
P’1, P’2, P’3									δ 6.96 (m, H10)

[Pt(Bequ)(PHEN)]$^+$

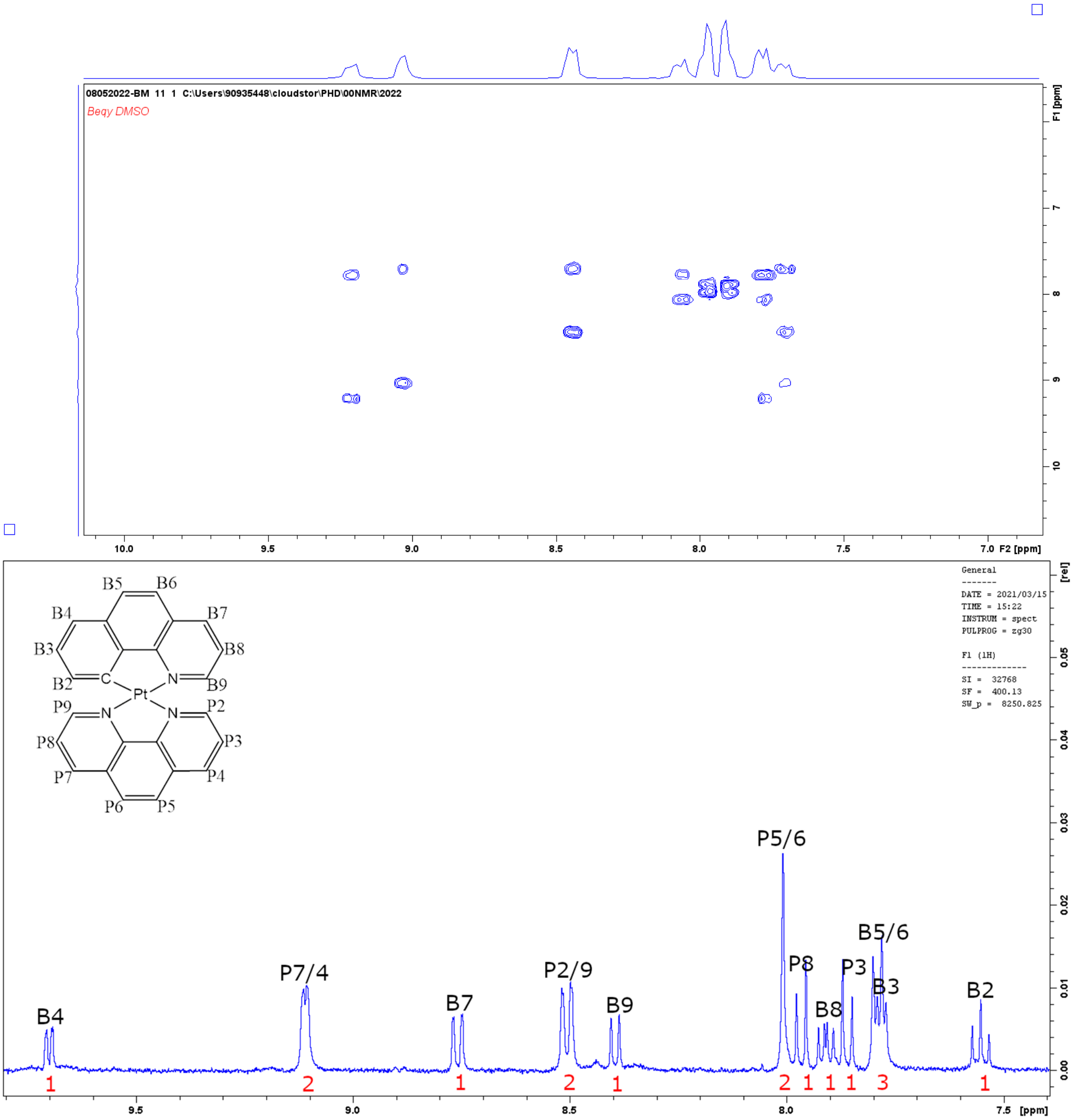
08052022-BM 11 1 C:\Users\90935448\cloudstor\PHD\00NMR\2022
Beqy DMSO
F1 [ppm]
F2 [ppm]
General
DATE = 2021/03/15
TIME = 15:22
INSTRUM = spect
PULPROG = zg30
F1 (1H)
SI = 32768
SF = 400.13
SW_p = 8250.825
[rel]
B4
P7/4
B7
P2/9
B9
P5/6
P8
B8
P3
B5/6
B3
B2
1 2 1 2 1 2 1 1 1 3 1
[ppm]

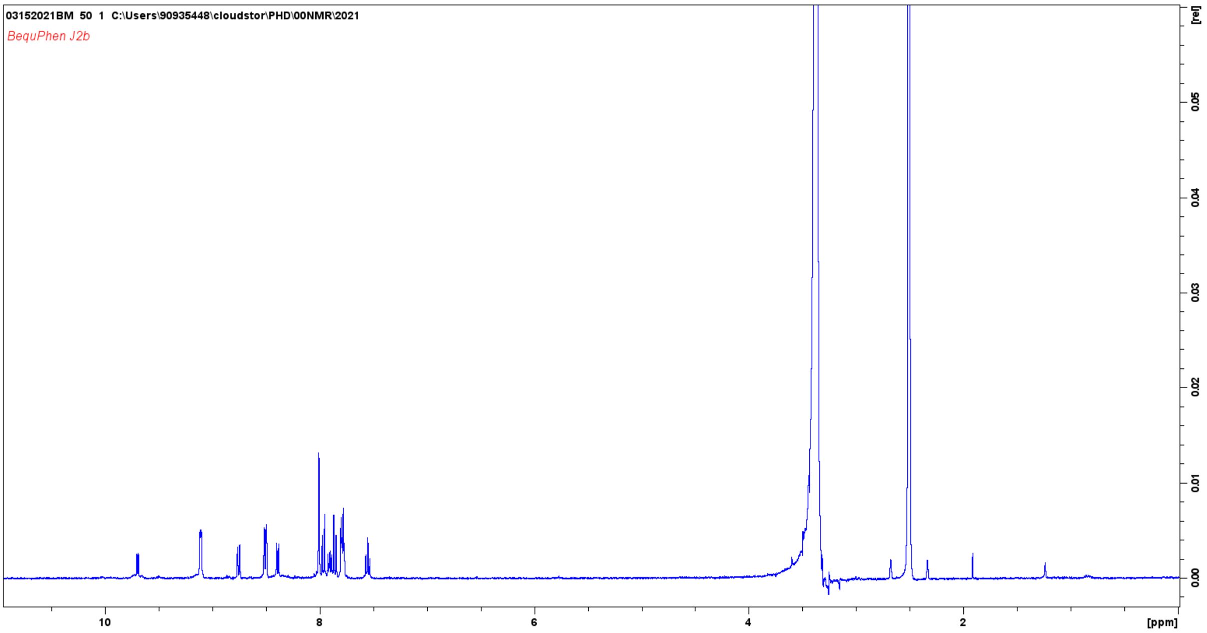

Figure S2. COSY spectra of Benzo[h]quinoline above and ^{1}H NMR spectra of Pt(II) $[Pt(Bequ)(PHEN)]^{+}$ in D_2O below.

[Pt(Bequ)(BPY)]$^+$

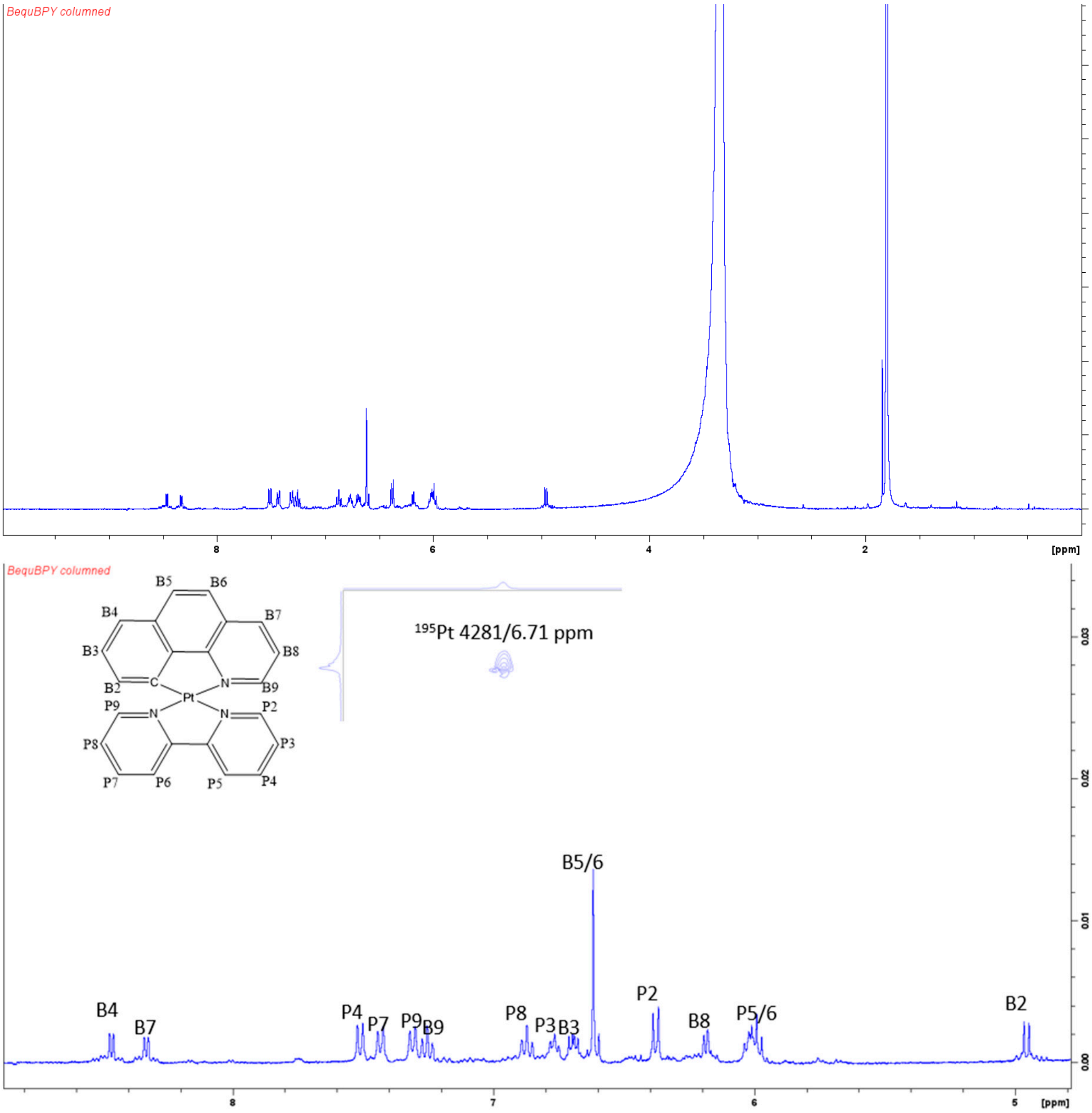

Figure S3. ^{1}H NMR spectra of Pt(II) [Pt(Bequ)(BPY)]$^+$ in D_2O annotated according to numbered structure top left with HMQC in D_2O top centre showing ^{195}Pt peak.

[Pt(Bequ)(44BPY)]$^+$

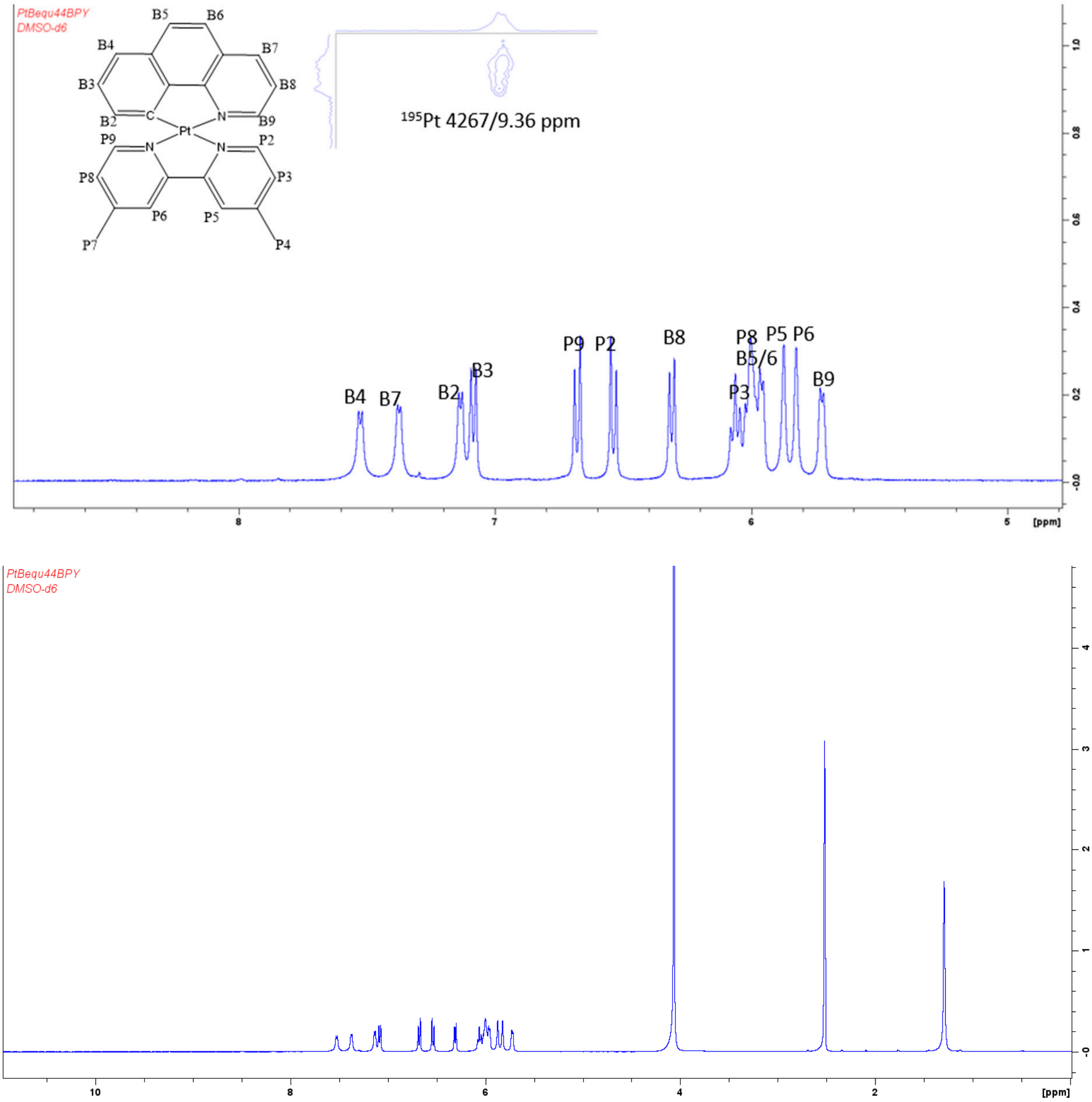

Figure S4. ^{1}H NMR spectra of Pt(II) [Pt(Bequ)(44BPY)]$^+$ in D_2O annotated according to numbered structure top left with HMQC in D_2O top centre showing ^{195}Pt peak.

$[Pt(Bequ)(TertBPY)]^+$

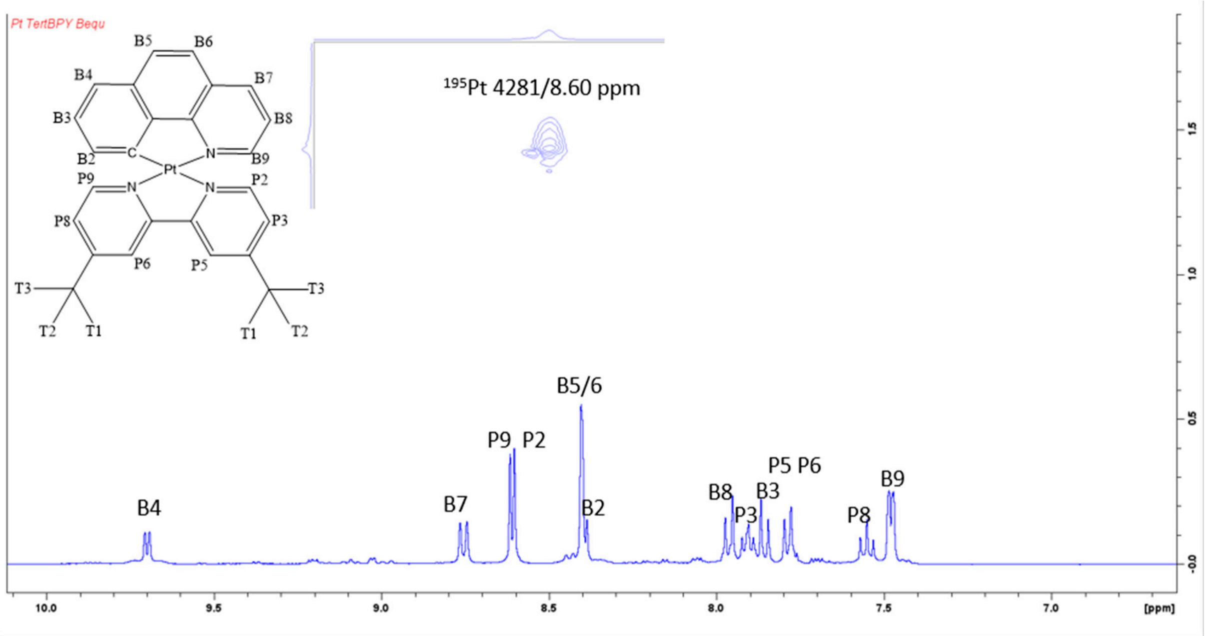

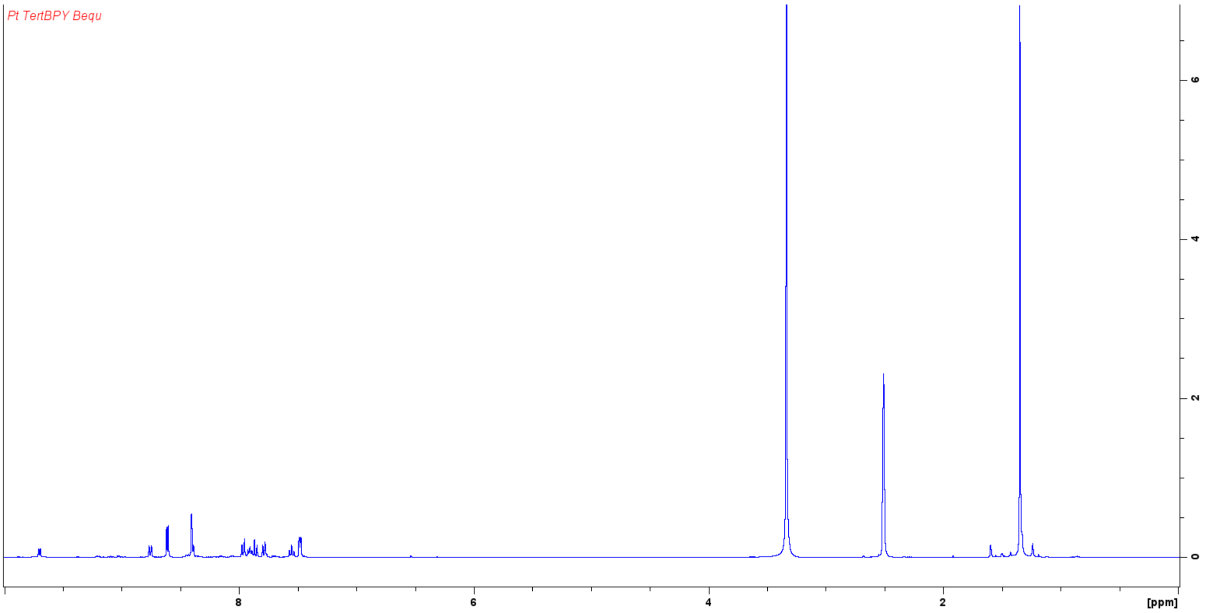

Figure S5. ^{1}H NMR spectra of Pt(II) $[Pt(Bequ)(TertBPY)]^+$ in D_2O annotated according to numbered structure top left with HMQC in D_2O top centre showing ^{195}Pt peak.

[Pt(Bequ)(5MePHEN)]$^{+}$

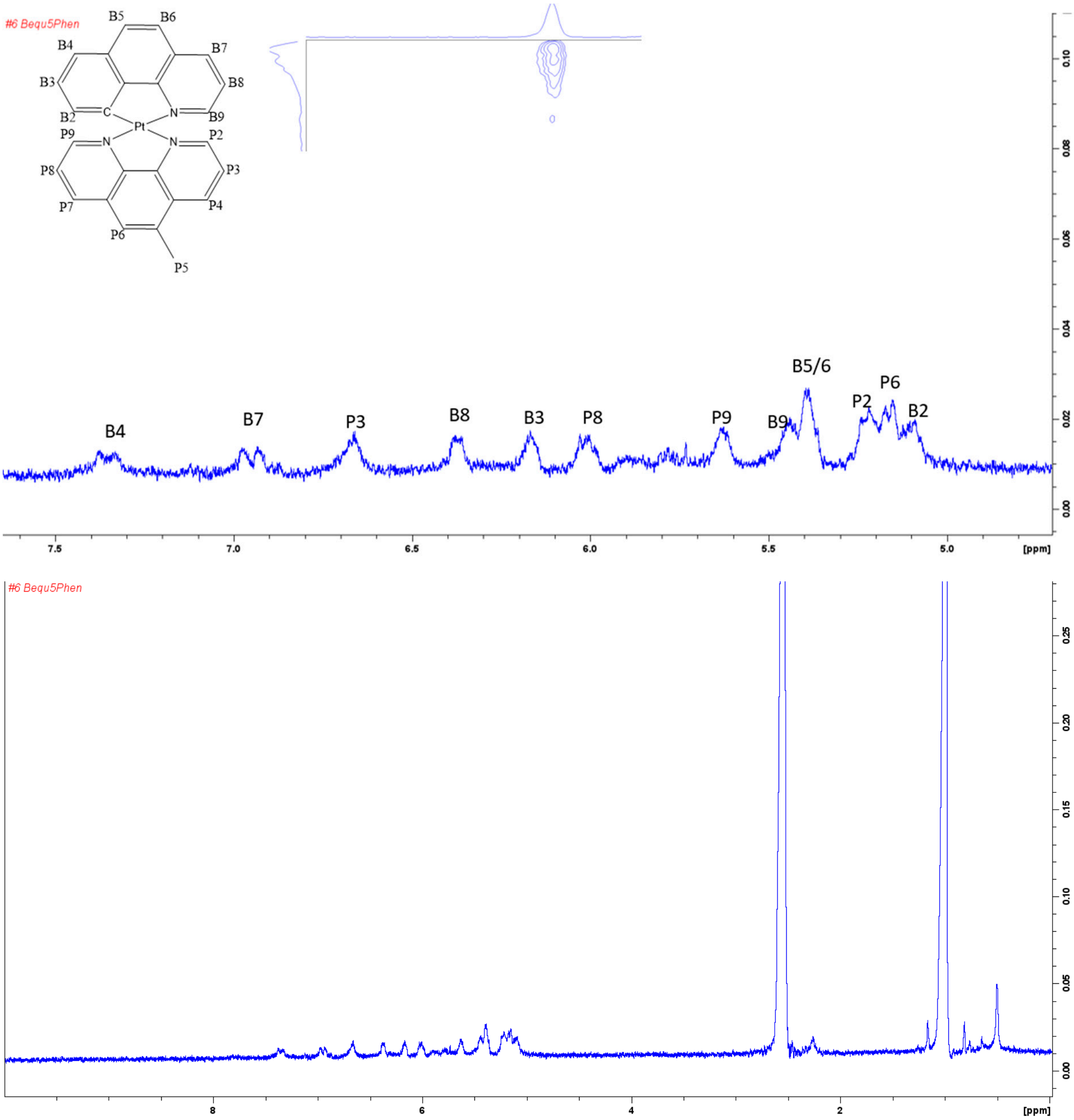

Figure S6. ^{1}H NMR spectra of Pt(II) [Pt(Bequ)(5MePHEN)]$^{+}$ in D_2O annotated according to numbered structure top left with HMQC in D_2O top centre showing ^{195}Pt peak.

[Pt(Bequ)(56MePHEN)]$^+$

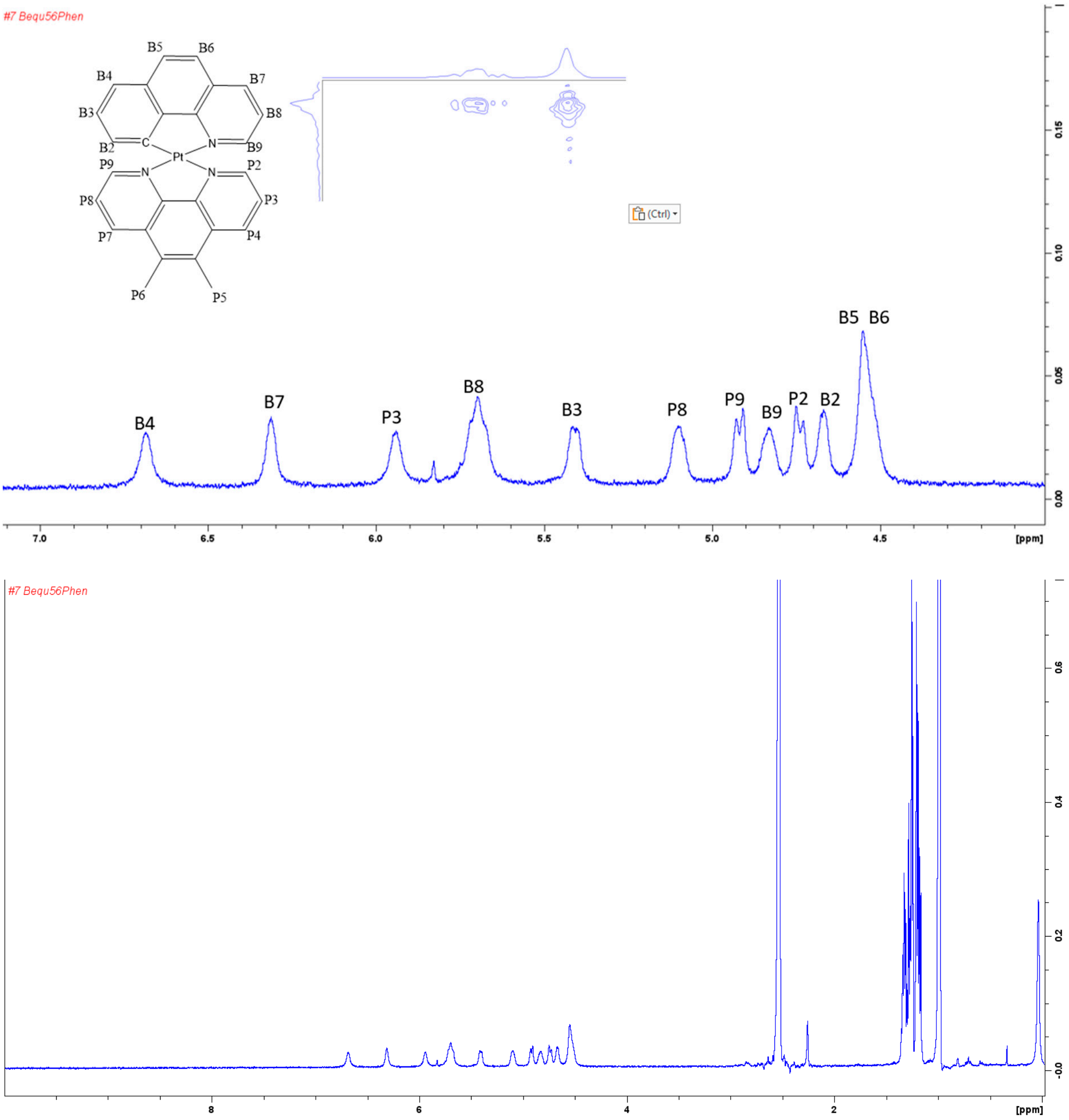

Figure S7. ^{1}H NMR spectra of Pt(II) [Pt(Bequ)(56MePHEN)]$^+$ in D_2O annotated according to numbered structure top left with HMQC in D_2O top centre showing ^{195}Pt peak.

[Pt(Bequ)(TMP)]+

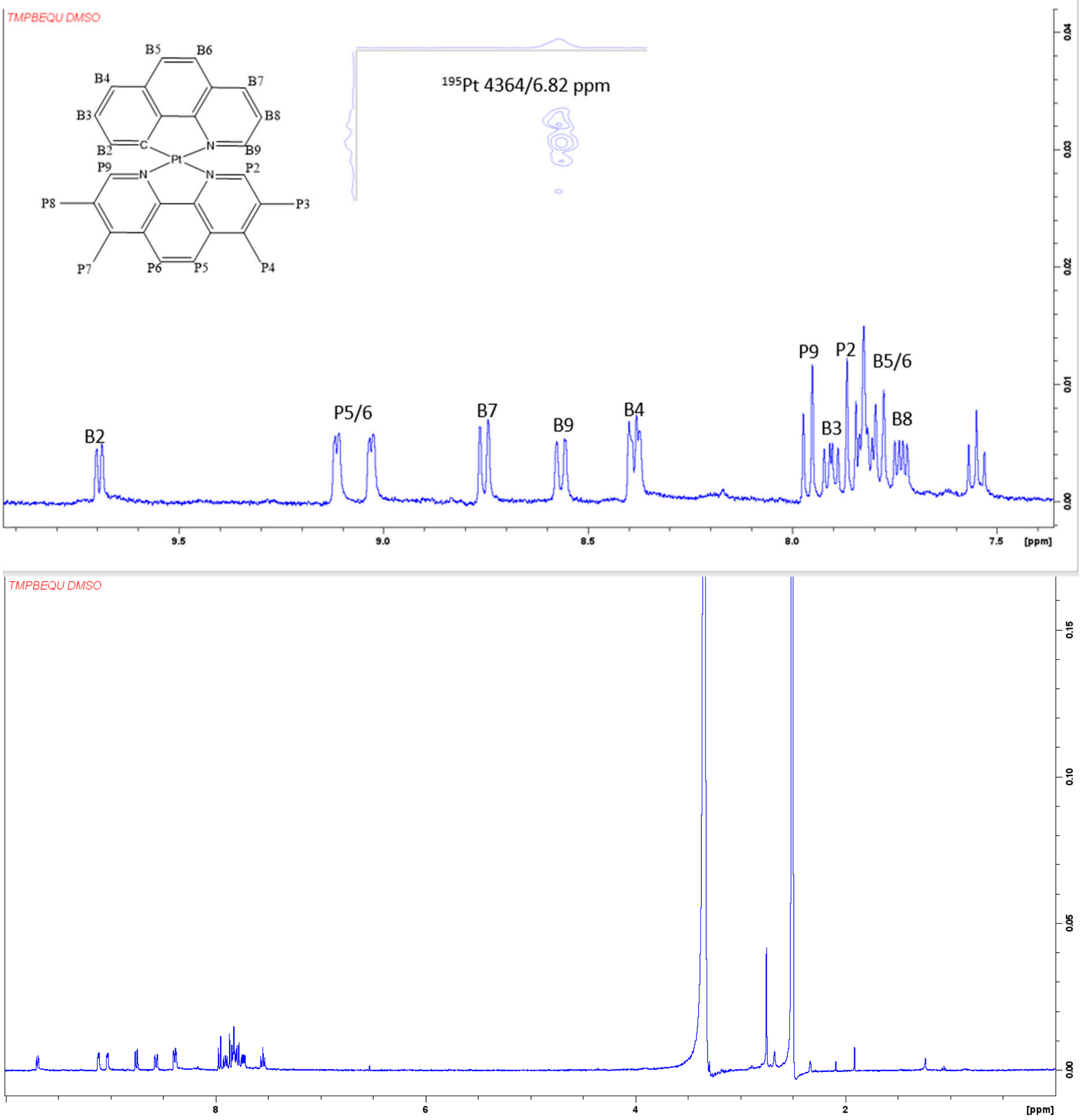

Figure S8. ^{1}H NMR spectra of Pt(II) [Pt(Bequ)(TMP)]+ in D_2O annotated according to numbered structure top left with HMQC in D_2O top centre showing ^{195}Pt peak.

[Pt(Bequ)(DPQ)]$^+$

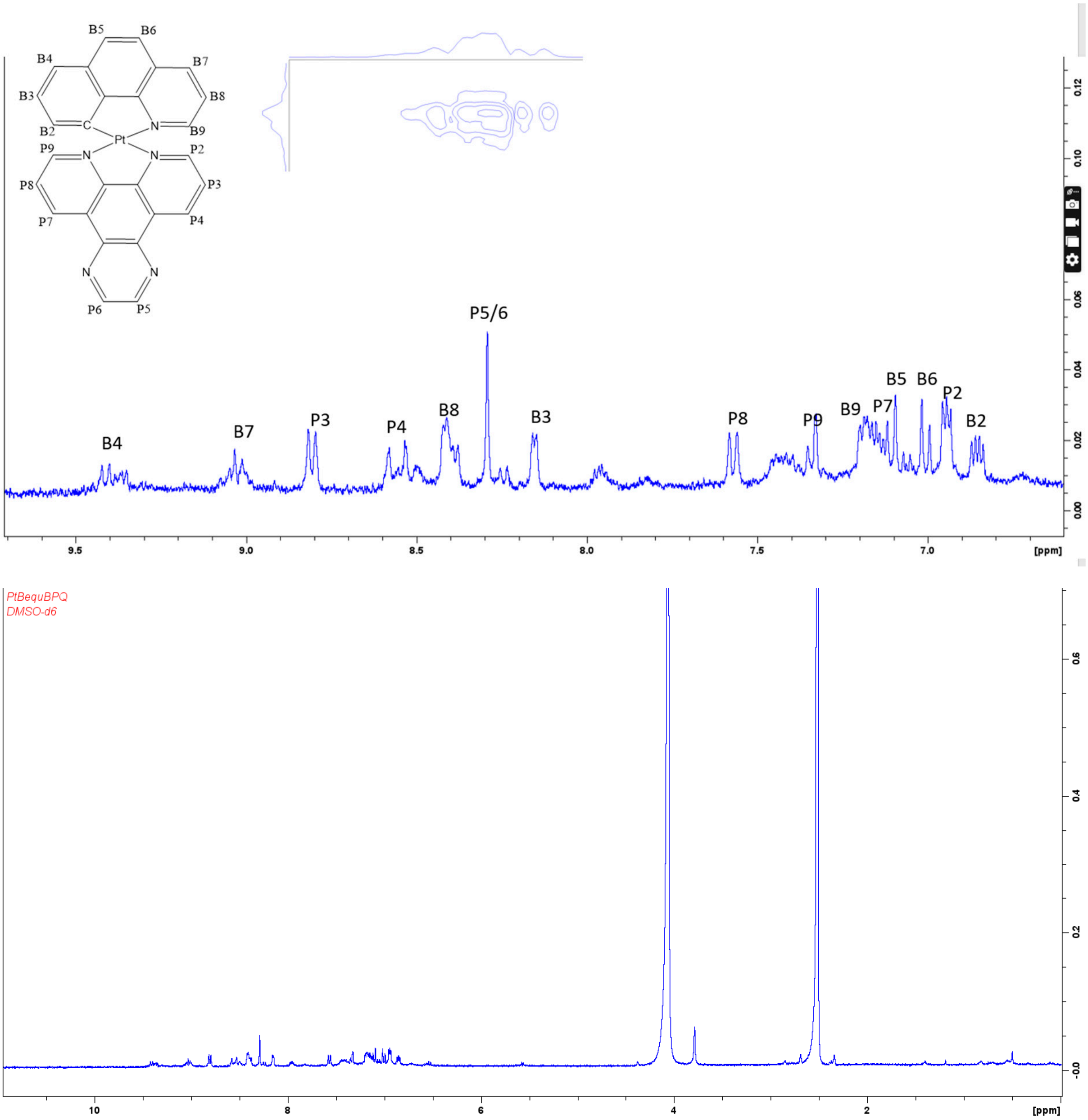

Figure S9. ^{1}H NMR spectra of Pt(II) [Pt(Bequ)(DPQ)]$^+$ in D_2O annotated according to numbered structure top left with HMQC in D_2O top centre showing ^{195}Pt peak.

[Pt(Bequ)(BathoPHEN)]$^+$

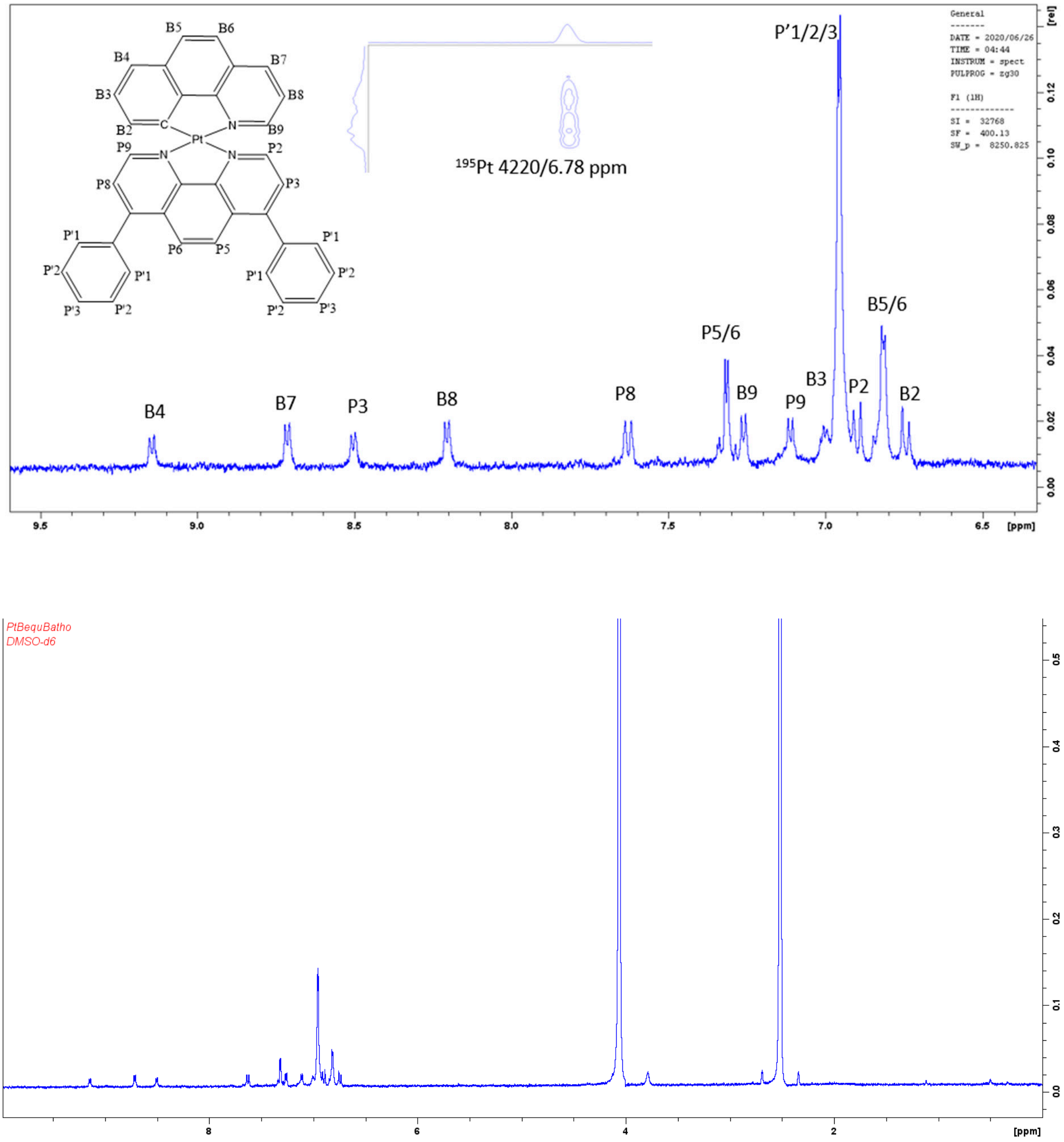

Figure S10. ^{1}H NMR spectra of Pt(II) [Pt(Bequ)(BathoPHEN)]$^+$ in D_2O annotated according to numbered structure top left with HMQC in D_2O top centre showing ^{195}Pt peak.

UV Spectra

Ligands

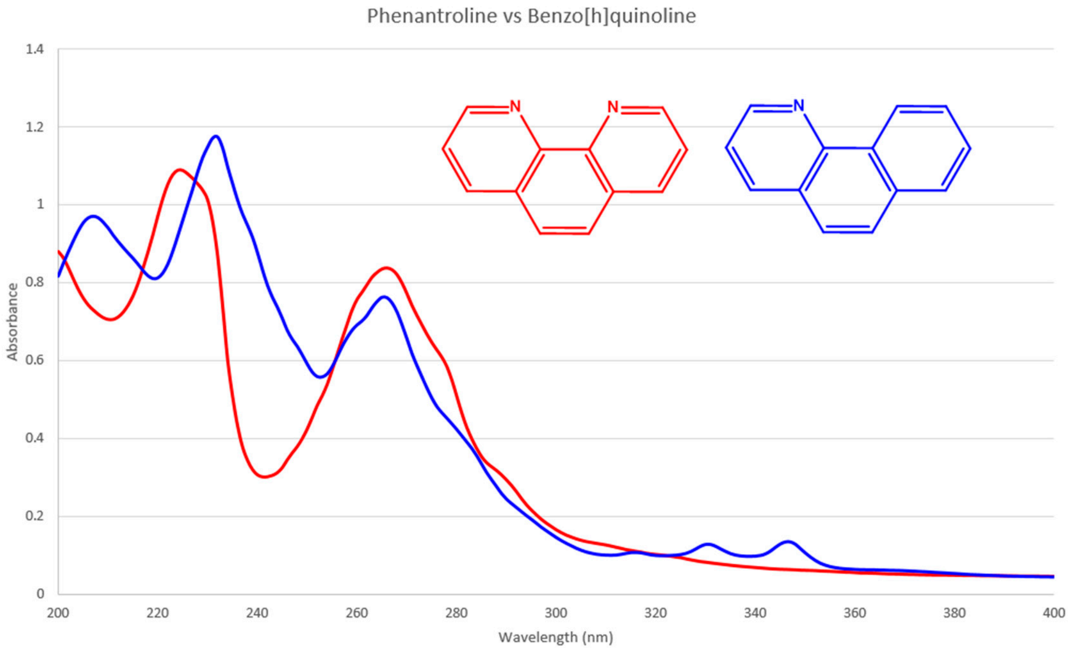

Figure S11. absorbance of phenathroline (red) and Benzo[h]quinolone (blue) at the same concentration in ethanol and water solution.

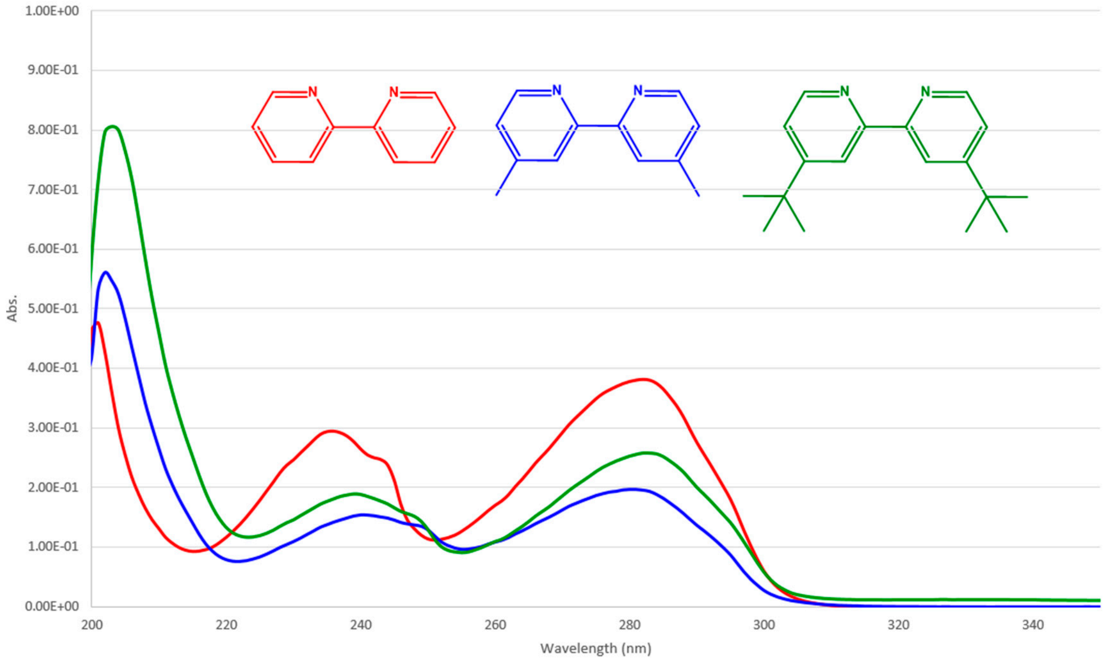

Figure S12. absorbance of BPY (red), 44BPY (blue), and TertBPY (green) at the same concentration in ethanol.

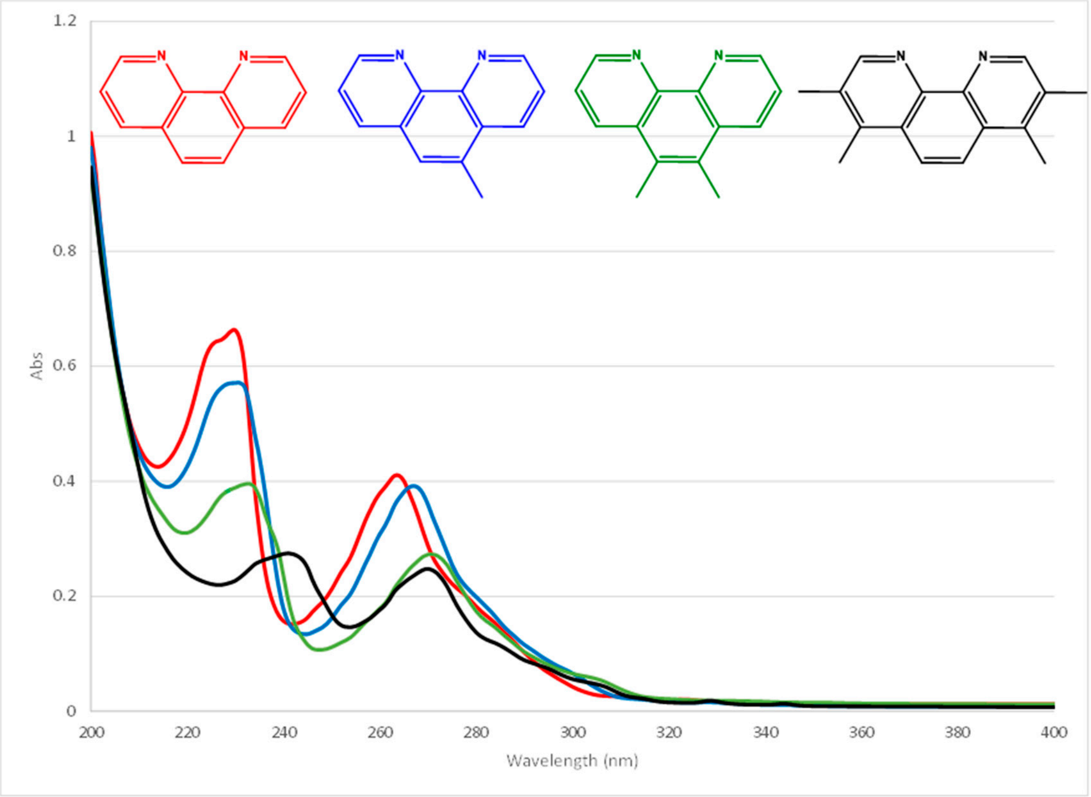

Figure S13. absorbance of PHEN (red), 5MePHEN (blue), 56MePHEN (green), and TMP (black) at the same concentration in ethanol.

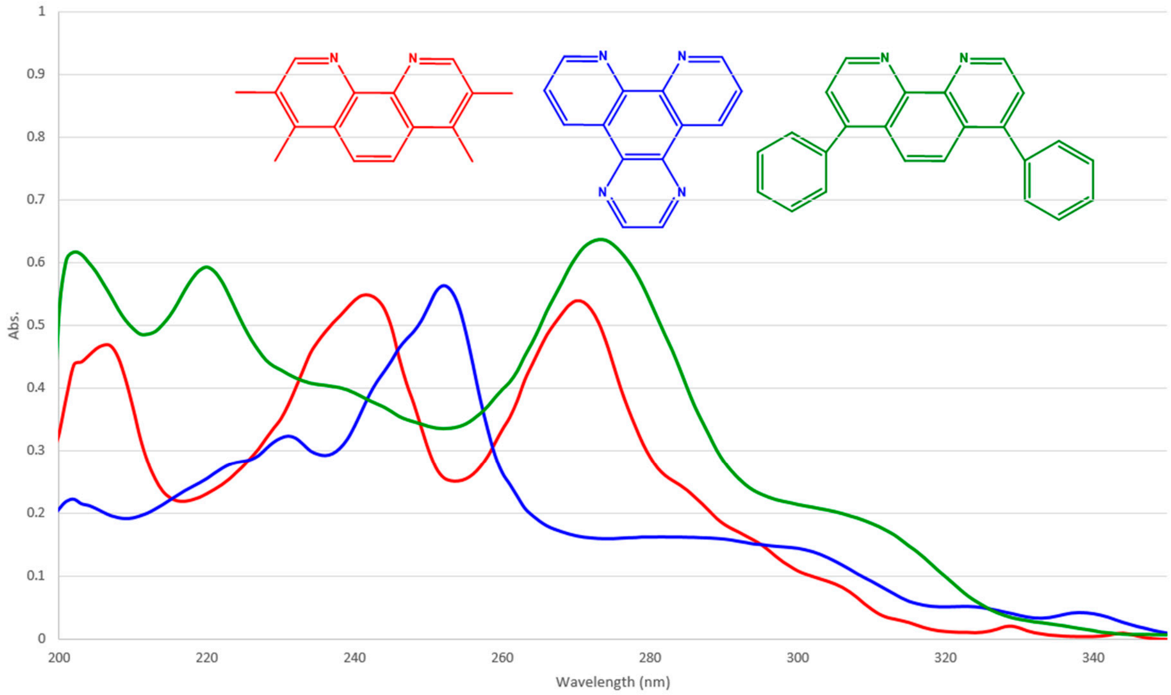

Figure S14. absorbance of TMP (red), DPQ (blue), and BathoPHEN (green) at the same concentration in ethanol.

Complexes

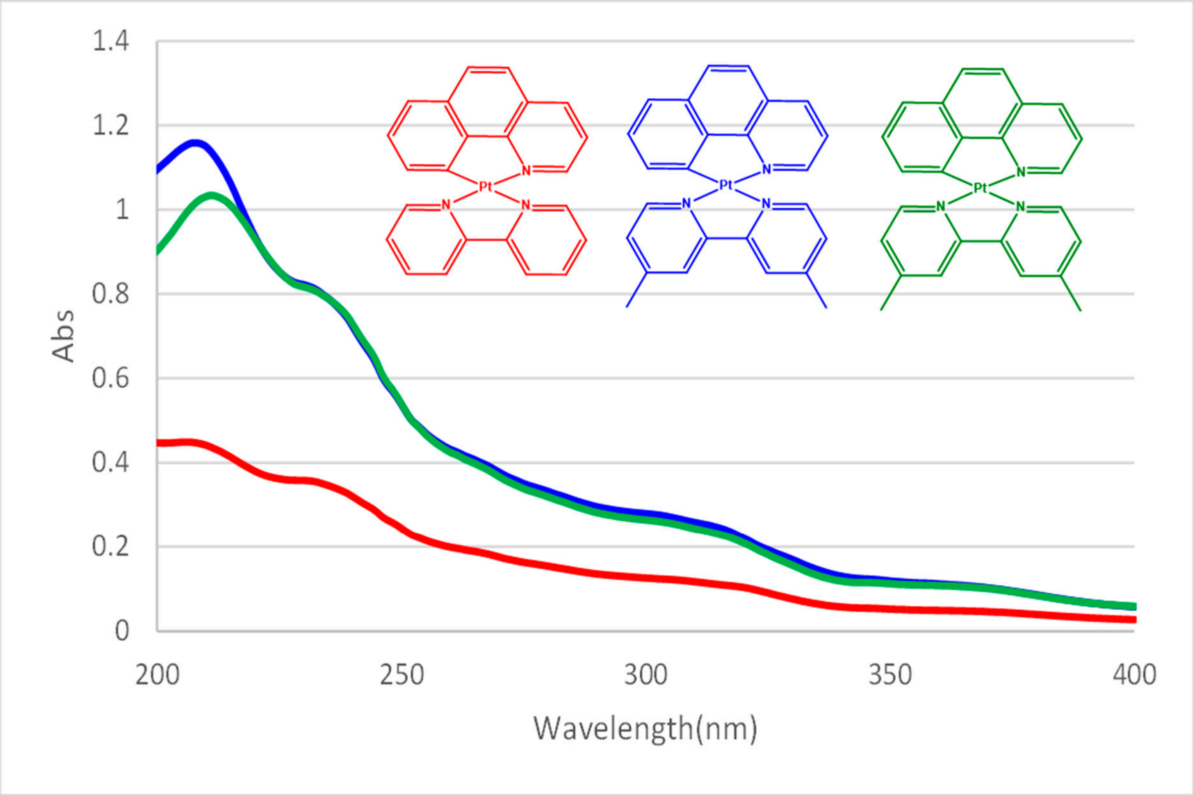

Figure S15. absorbance of [Pt(Bequ)(BPY)]$^+$ (red), [Pt(Bequ)(44BPY)]$^+$ (blue), and [Pt(Bequ)(TertBPY)]$^+$ (green) at the same concentration in water.

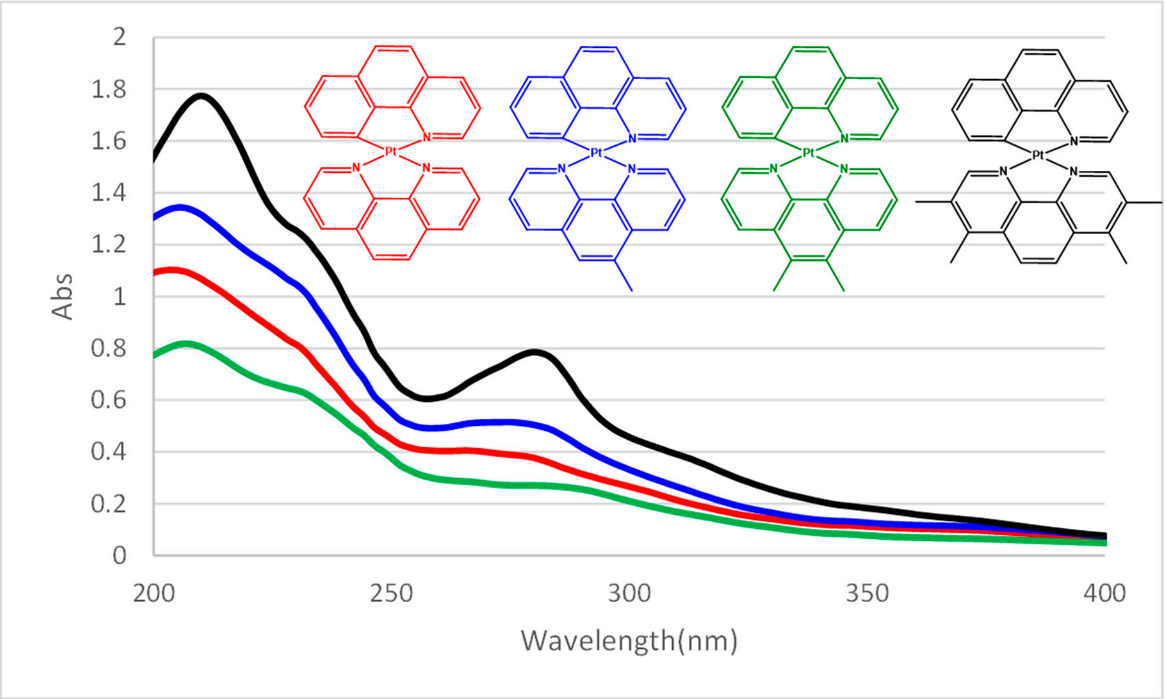

Figure S16. absorbance of [Pt(Bequ)(PHEN)]$^+$ (red), [Pt(Bequ)(5MePHEN)]$^+$ (blue), [Pt(Bequ)(56MePHEN)]$^+$ (green) and [Pt(Bequ)(TMP)]$^+$ (black), at the same concentration in water.

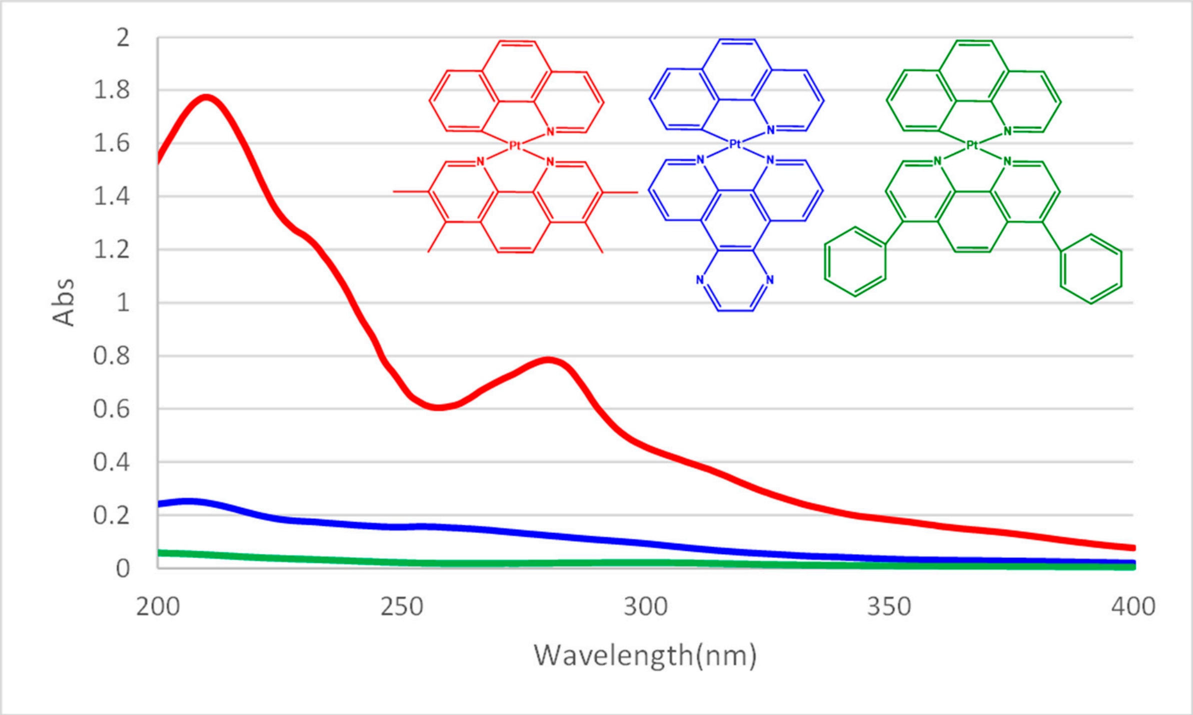

Figure S17. absorbance of [Pt(Bequ)(TMP)]$^+$ (red), [Pt(Bequ)(DPQ)]$^+$ (blue), and [Pt(Bequ)(BathoPHEN)]$^+$ (green) concentration in water (complexes 8 and 9 are not very soluble in water).

Table S2. extinction coefficients of ligands in EtOH. (EtOH used as not all complexes were soluble in water)

Ligand	UV / λ_{max} (nm) (ε/mol^{-1}.dm^{3}cm^{-1}) × 10^2
BPY	282(270.25) 236(341.07)
44BPY	201(367.53),281(145.85)
TertBPY	239(340.60),284(519.80)
PHEN	230(551.80),264(719.21)
5MePHEN	231(682.55),269(766.56)
56MePHEN	233(489.93),271(508.90)
TMP	278(473.18), 226(529.19)
DPQ	231(823.72),252(1621.09)
BathoPHEN	206(1502.47), 278(1760.42)
Bequ	207(1185.96),265(1156.51)

Molar Absorption Coefficients

[Pt(Bequ)(BPY)]$^+$

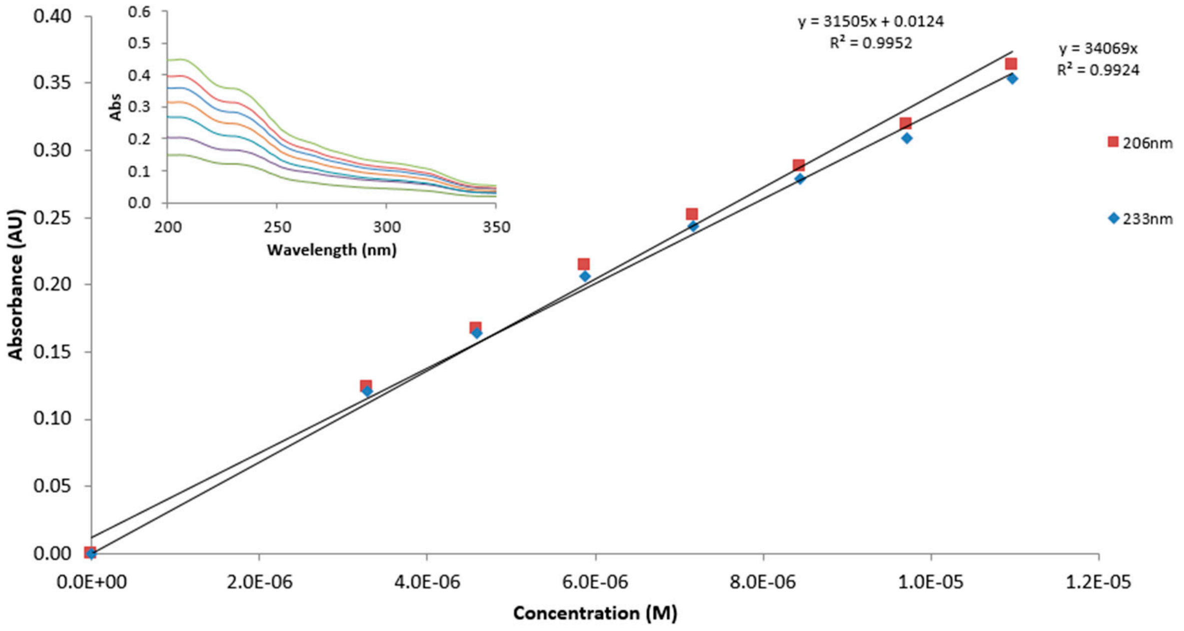

Figure S18. Titration of a stock solution of [Pt(Bequ)(BPY)]$^+$ into a known concentration in H_2O and the resulting extinction coefficient calculated based on the two main peaks at 206 nm (red) and 233 nm (blue).

[Pt(Bequ)(44BPY)]$^{+}$

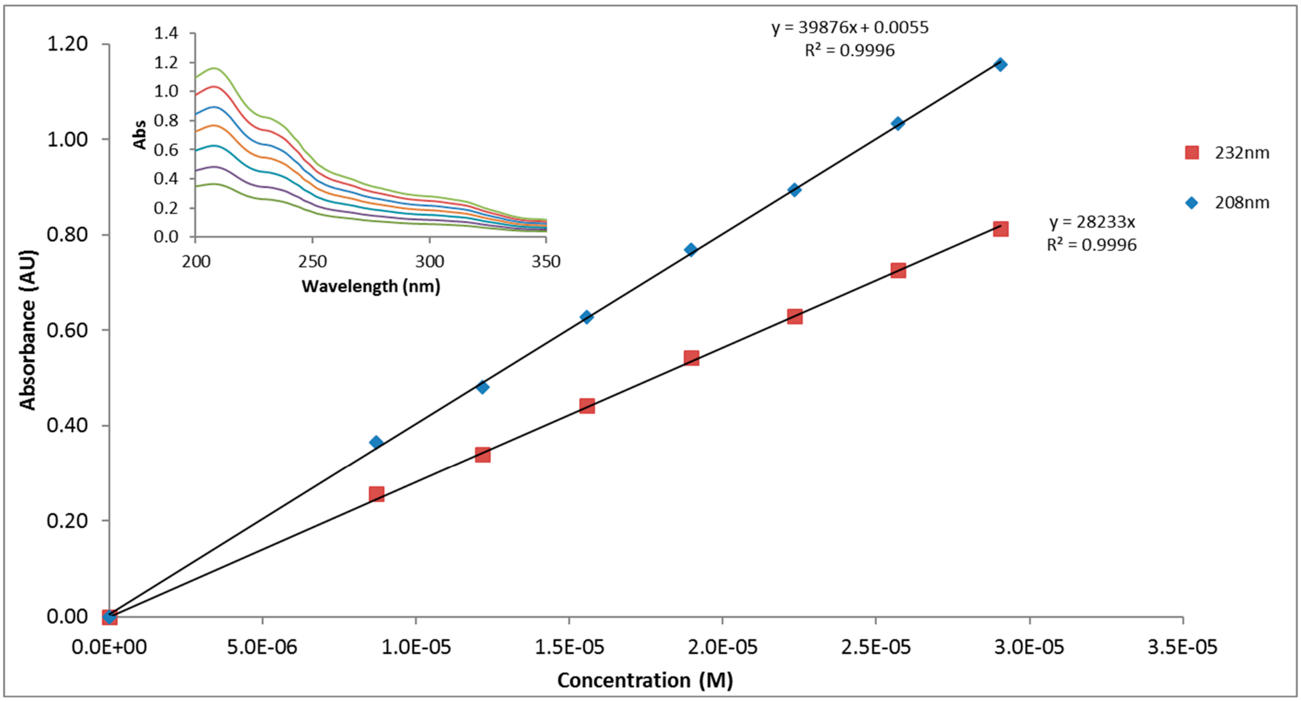

Figure S19. Titration of a stock solution of [Pt(Bequ)(44BPY)]$^{+}$ into a known concentration in H_2O and the resulting extinction coefficient calculated based on the two main peaks at 232 nm (red) and 208 nm (blue).

[Pt(Bequ)(TertBPY)]$^{+}$

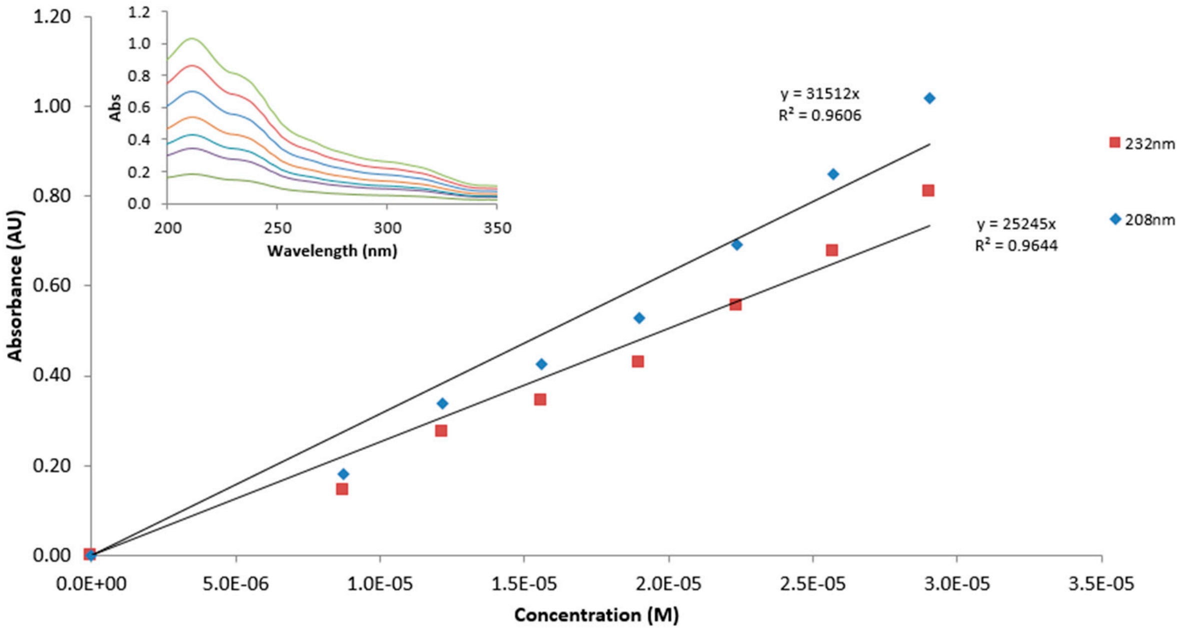

Figure S20. Titration of a stock solution of [Pt(Bequ)(tertBPY)]$^{+}$ into a known concentration in H_2O and the resulting extinction coefficient calculated based on the two main peaks at 232 nm (red) and 208 nm (blue).

[Pt(Bequ)(PHEN)]$^+$

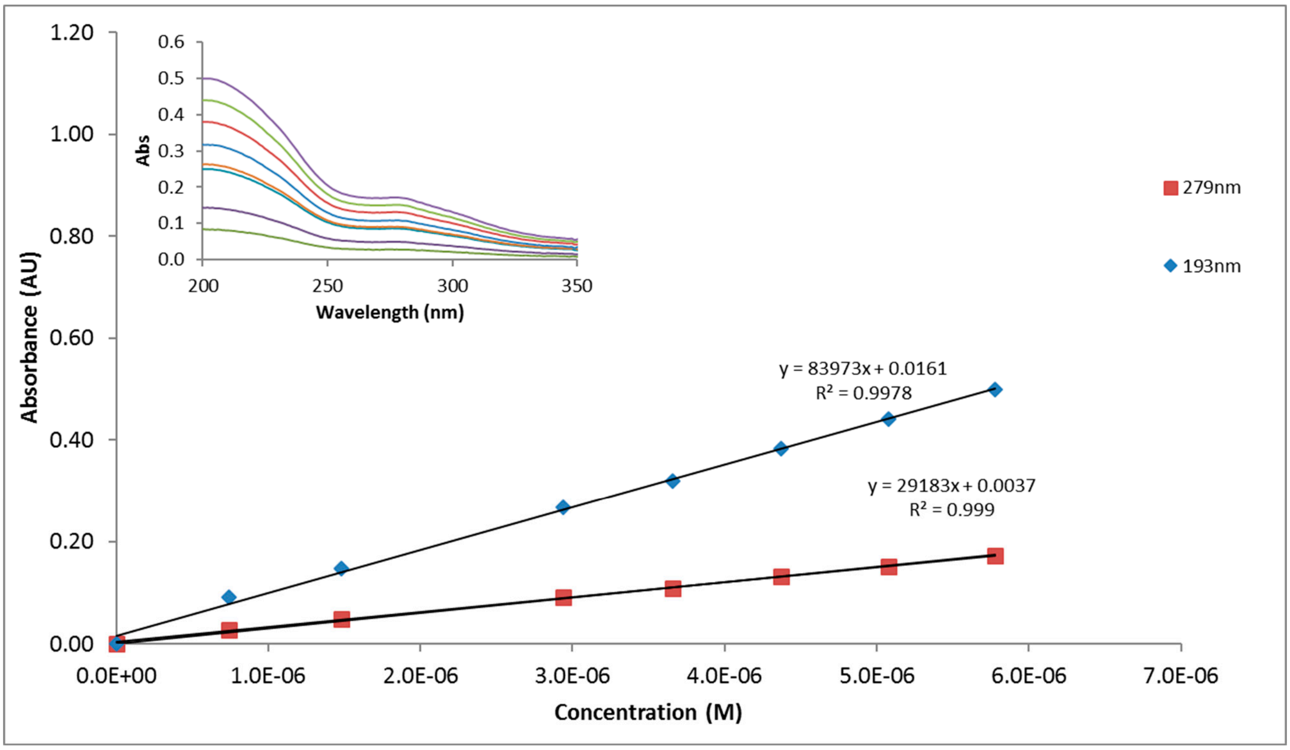

Figure S21. Titration of a stock solution of [Pt(Bequ)(Phen)]$^+$ into a known concentration in H_2O and the resulting extinction coefficient calculated based on the two main peaks at 279 nm (red) and 293 nm (blue).

[Pt(Bequ)(5MePHEN)]$^+$

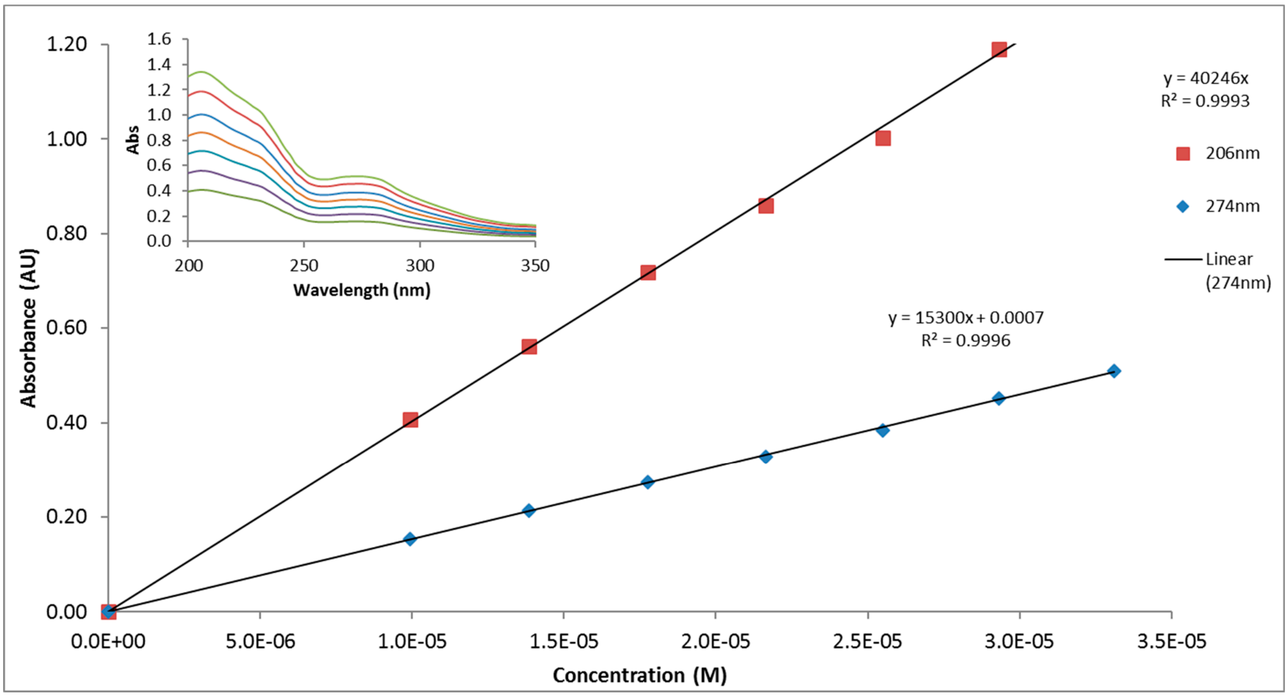

Figure S22. Titration of a stock solution of [Pt(Bequ)(5MePhen)]$^+$ into a known concentration in H_2O and the resulting extinction coefficient calculated based on the two main peaks at 206 nm (red) and 274 nm (blue).

[Pt(Bequ)(56MePHEN)]$^+$

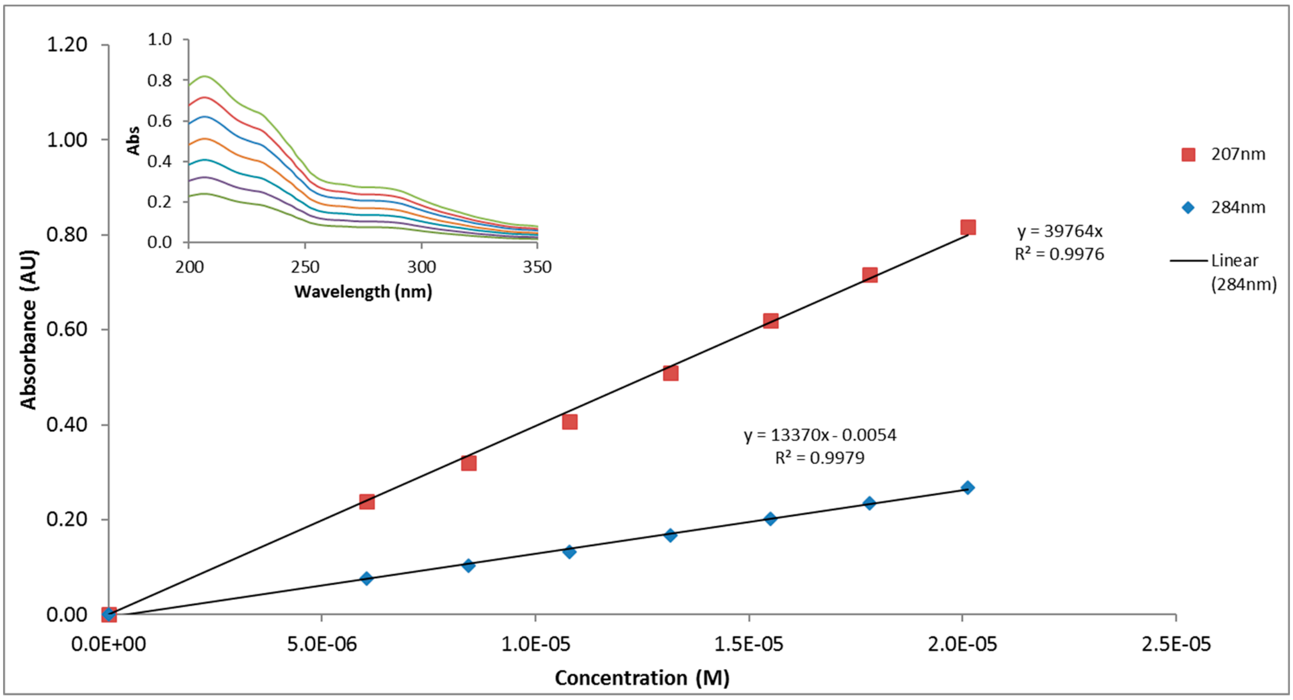

Figure S23. Titration of a stock solution of [Pt(Bequ)(56Me_2Phen)]$^+$ into a known concentration in H_2O and the resulting extinction coefficient calculated based on the two main peaks at 207 nm (red) and 284 nm (blue).

[Pt(Bequ)(TMP)]$^+$

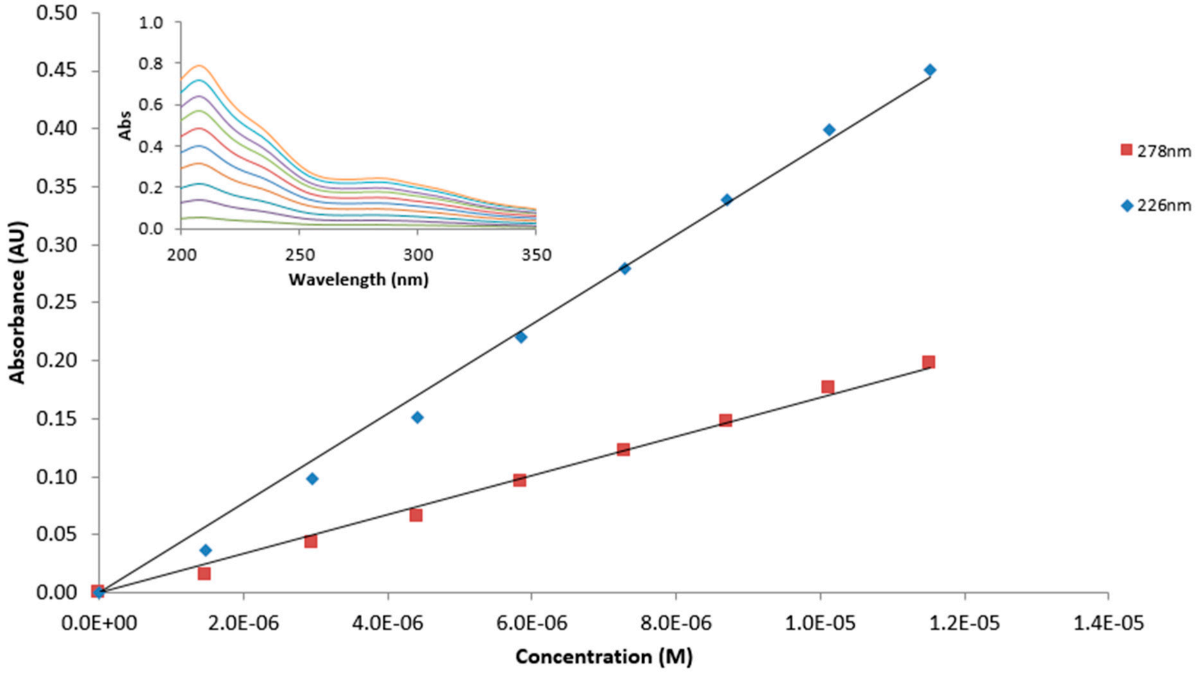

Figure S24. Titration of a stock solution of [Pt(Bequ)(TMP)]$^+$ into a known concentration in H_2O and the resulting extinction coefficient calculated based on the two main peaks at 278 nm (red) and 226 nm (blue).

[Pt(Bequ)(DPQ)]+

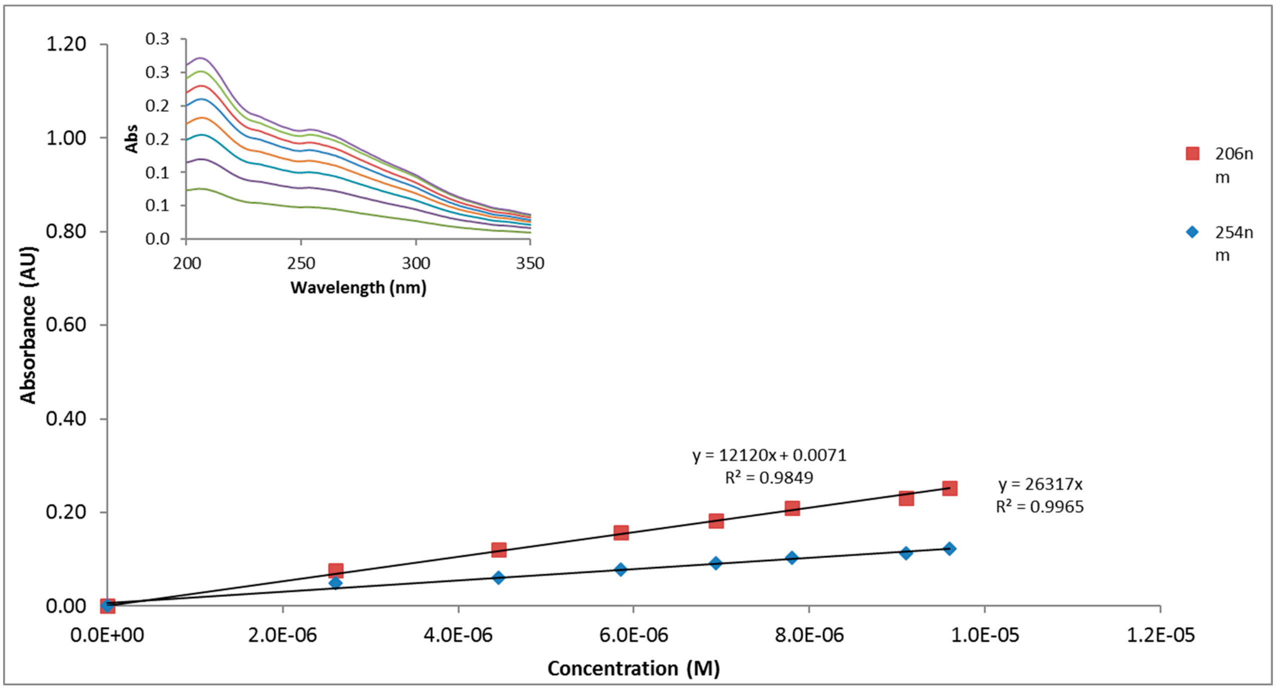

Figure S25. Titration of a stock solution of [Pt(Bequ)(DQP)]+ into a known concentration in H_2O and the resulting extinction coefficient calculated based on the two main peaks at 206 nm (red) and 254 nm (blue).

[Pt(Bequ)(BathoPHEN)]+

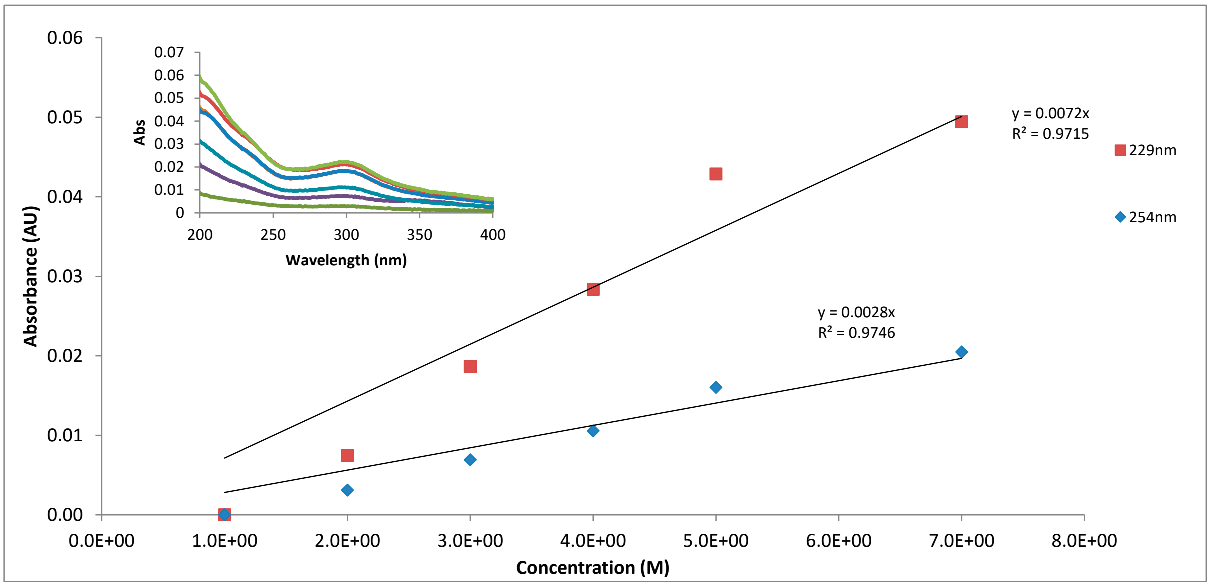

Figure S26. Titration of a stock solution of [Pt(Bequ)(Batho)]+ into a known concentration in H_2O and the resulting extinction coefficient calculated based on the two main peaks at 254 nm (red) and 229 nm (blue).

HPLC

All HPLC's show purity over 95%

[Pt(Bequ)(BPY)]$^+$

Acq. Operator : SYSTEM Seq. Line : 1
Acq. Instrument : LC1260 Location : Vial 61
Injection Date : 11/12/2020 11:59:19 AM Inj : 1
Inj Volume : 10.000 µl
Method : D:\BRONDWYN\DATA\20201204_BEQU COLUMNED 2020-11-12 11-58-00\10 TO 100 OVER 15 MINS 10UL.M (Sequence Method)
Last changed : 11/12/2020 11:58:00 AM by SYSTEM

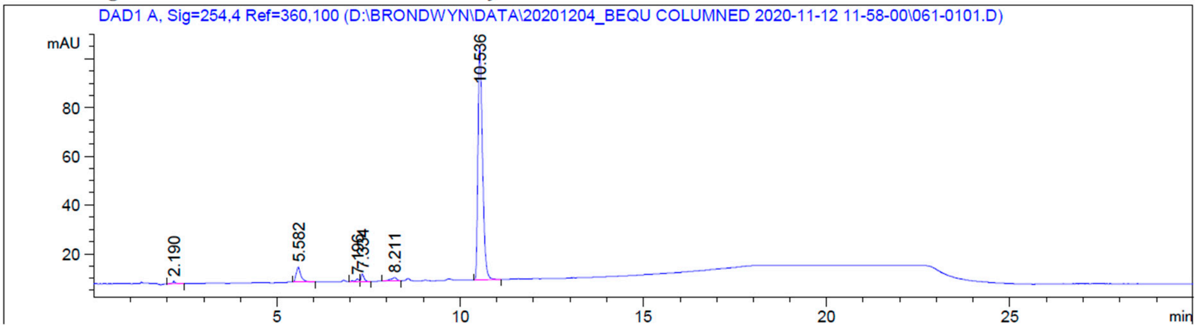

Figure S27. HPLC spectrum of [Pt(Bequ)(BPY)]$^+$ measured on a Agilent Technologies 1260 Infinity machine equipped with a Phenomenex Onyx™ Monolithic C18 reverse phase column (100 × 4.6 mm, 130 Å). Sample solutions were made up in H_2O and injected at a 0-100 gradient A to B over 15 minutes with a 15 minute flush in-between samples.

[Pt(Bequ)(44BPY)]$^+$

Acq. Operator : SYSTEM Seq. Line : 2
Acq. Instrument : LC1260 Location : Vial 62
Injection Date : 11/12/2020 12:30:43 PM Inj : 1
Inj Volume : 10.000 µl
Method : D:\BRONDWYN\DATA\20201204_BEQU COLUMNED 2020-11-12 11-58-00\10 TO 100 OVER 15 MINS 10UL.M (Sequence Method)
Last changed : 11/12/2020 11:58:00 AM by SYSTEM

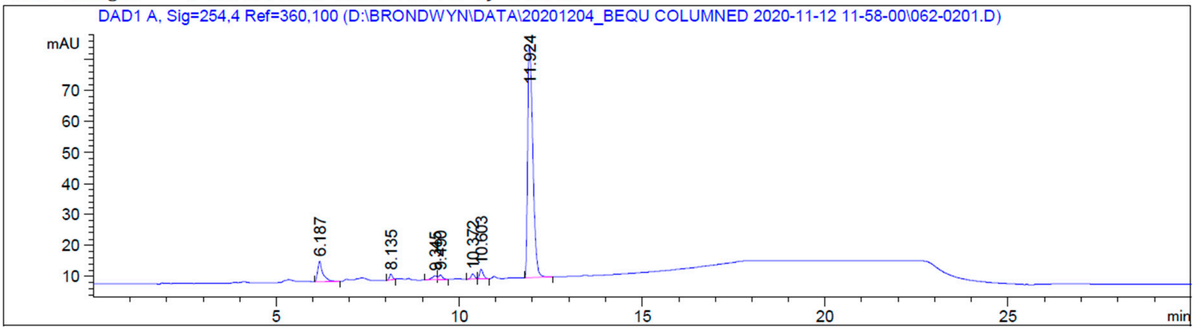

Figure S28. ESIMS spectrum of [Pt(Bequ)(44BPY)]$^+$ measured on a Agilent Technologies 1260 Infinity machine equipped with a Phenomenex Onyx™ Monolithic C18 reverse phase column (100 × 4.6 mm, 130 Å). Sample solutions were made up in H_2O and injected at a 0-100 gradient A to B over 15 minutes with a 15 minute flush in-between samples.

[Pt(Bequ)(TertBPY)]+

Acq. Operator : SYSTEM   Seq. Line : 3
Acq. Instrument : LC1260   Location : Vial 23
Injection Date : 11/5/2020 12:52:54 PM   Inj : 1
Inj Volume : 5.000 µl
Method : C:\CHEM32\1\DATA\BM 11052020 2020-11-05 11-48-44\10 TO 100 OVER 15 MINS 5UL .M (Sequence Method)
Last changed : 11/5/2020 11:48:44 AM by SYSTEM

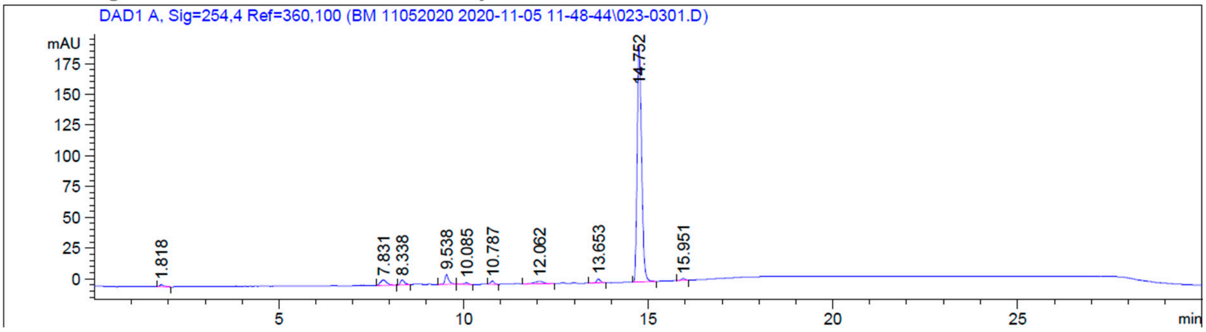

Figure S29. ESIMS spectrum of [Pt(Bequ)(TertBPY)]+ measured on a Agilent Technologies 1260 Infinity machine equipped with a Phenomenex Onyx™ Monolithic C18 reverse phase column (100 × 4.6 mm, 130 Å). Sample solutions were made up in H_2O and injected at a 0-100 gradient A to B over 15 minutes with a 15 minute flush in-between samples.

[Pt(Bequ)(PHEN)]+

Acq. Operator : SYSTEM   Seq. Line : 4
Acq. Instrument : LC1260   Location : Vial 64
Injection Date : 11/12/2020 1:33:30 PM   Inj : 1
Inj Volume : 10.000 µl
Method : D:\BRONDWYN\DATA\20201204_BEQU COLUMNED 2020-11-12 11-58-00\10 TO 100 OVER 15 MINS 10UL.M (Sequence Method)
Last changed : 11/12/2020 11:58:00 AM by SYSTEM

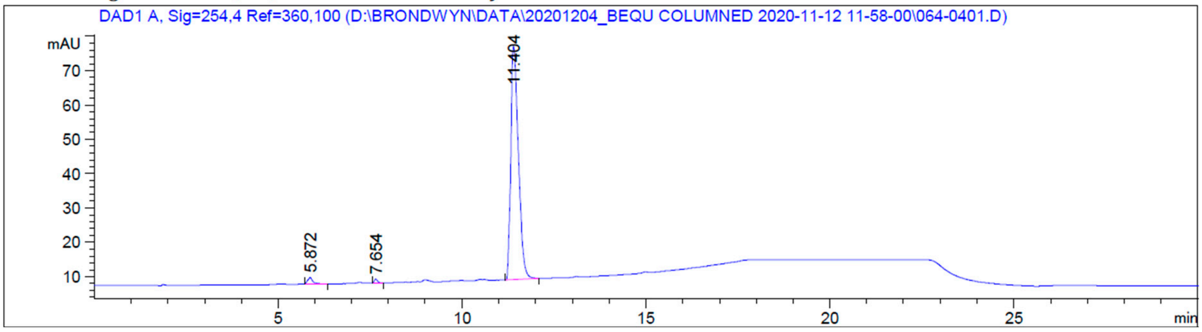

Figure S30. ESIMS spectrum of [Pt(Bequ)(PHEN)]+ measured on a Agilent Technologies 1260 Infinity machine equipped with a Phenomenex Onyx™ Monolithic C18 reverse phase column (100 × 4.6 mm, 130 Å). Sample solutions were made up in H_2O and injected at a 0-100 gradient A to B over 15 minutes with a 15 minute flush in-between samples.

[Pt(Bequ)(5MePHEN)]$^+$

```
Acq. Operator    : SYSTEM                          Seq. Line :   5
Acq. Instrument  : LC1260                           Location : Vial 65
Injection Date   : 11/12/2020 2:04:55 PM                 Inj :   1
                                                  Inj Volume : 10.000 µl
Method           : D:\BRONDWYN\DATA\20201204_BEQU COLUMNED 2020-11-12 11-58-00\10 TO 100 OVER
                   15 MINS 10UL.M (Sequence Method)
Last changed     : 11/12/2020 11:58:00 AM by SYSTEM
```

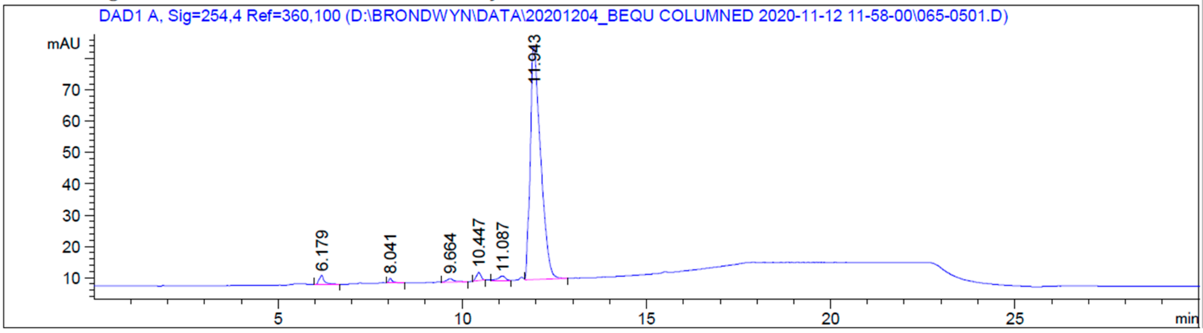

Figure S31. ESIMS spectrum of [Pt(Bequ)(5MePHEN)]$^+$ measured on a Agilent Technologies 1260 Infinity machine equipped with a Phenomenex Onyx™ Monolithic C18 reverse phase column (100 × 4.6 mm, 130 Å). Sample solutions were made up in H_2O and injected at a 0-100 gradient A to B over 15 minutes with a 15 minute flush in-between samples.

[Pt(Bequ)(56MePHEN)]$^+$

```
Acq. Operator    : SYSTEM                          Seq. Line :   6
Acq. Instrument  : LC1260                           Location : Vial 66
Injection Date   : 11/12/2020 2:36:17 PM                 Inj :   1
                                                  Inj Volume : 10.000 µl
Method           : D:\BRONDWYN\DATA\20201204_BEQU COLUMNED 2020-11-12 11-58-00\10 TO 100 OVER
                   15 MINS 10UL.M (Sequence Method)
Last changed     : 11/12/2020 11:58:00 AM by SYSTEM
```

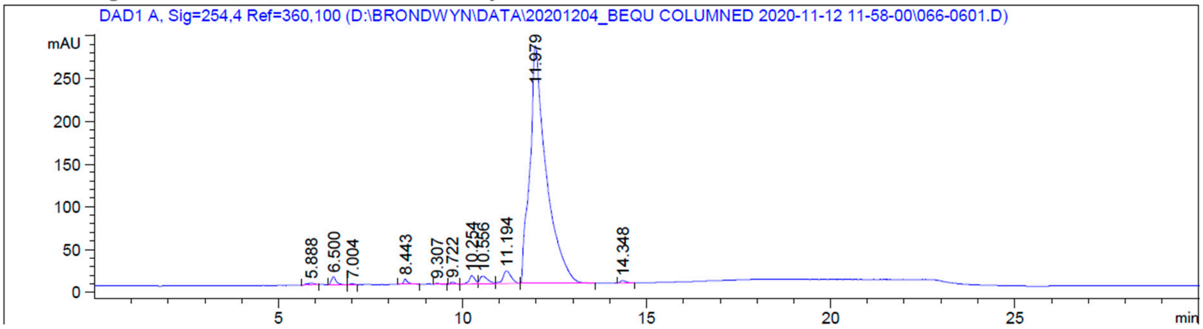

Figure S32. ESIMS spectrum of [Pt(Bequ)(56Me$_2$PHEN)]$^+$ measured on a Agilent Technologies 1260 Infinity machine equipped with a Phenomenex Onyx™ Monolithic C18 reverse phase column (100 × 4.6 mm, 130 Å). Sample solutions were made up in H_2O and injected at a 0-100 gradient A to B over 15 minutes with a 15 minute flush in-between samples.

[Pt(Bequ)(TMP)]$^+$

```
Acq. Operator    : SYSTEM                          Seq. Line :   7
Acq. Instrument  : LC1260                           Location : Vial 27
Injection Date   : 11/5/2020 2:58:04 PM                  Inj :   1
                                                  Inj Volume : 5.000 µl
Method           : C:\CHEM32\1\DATA\BM 11052020 2020-11-05 11-48-44\10 TO 100 OVER 15 MINS 5UL
                   .M (Sequence Method)
Last changed     : 11/5/2020 11:48:44 AM by SYSTEM
```

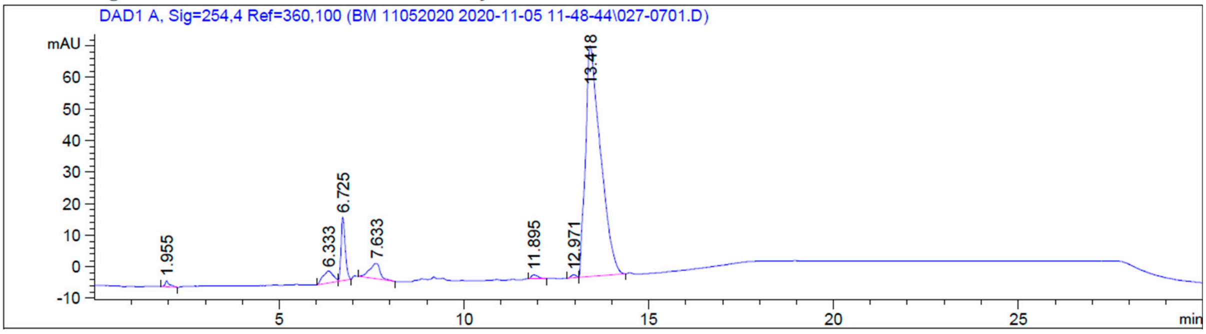

Figure S33. ESIMS spectrum of [Pt(Bequ)(TMP)]$^+$ measured on a Agilent Technologies 1260 Infinity machine equipped with a Phenomenex Onyx™ Monolithic C18 reverse phase column (100 × 4.6 mm, 130 Å). Sample solutions were made up in H_2O and injected at a 0-100 gradient A to B over 15 minutes with a 15 minute flush in-between samples.

[Pt(Bequ)(DPQ)]$^+$

```
Acq. Operator    : SYSTEM                          Seq. Line :   2
Acq. Instrument  : LC1260                           Location : Vial 72
Injection Date   : 11/25/2020 2:05:50 PM                 Inj :   1
                                                  Inj Volume : 10.000 µl
Method           : D:\BRONDWYN\DATA\251120V2_REDO 2020-11-25 13-33-08\10 TO 100 OVER 15 MINS
                   10UL.M (Sequence Method)
Last changed     : 11/25/2020 1:33:08 PM by SYSTEM
```

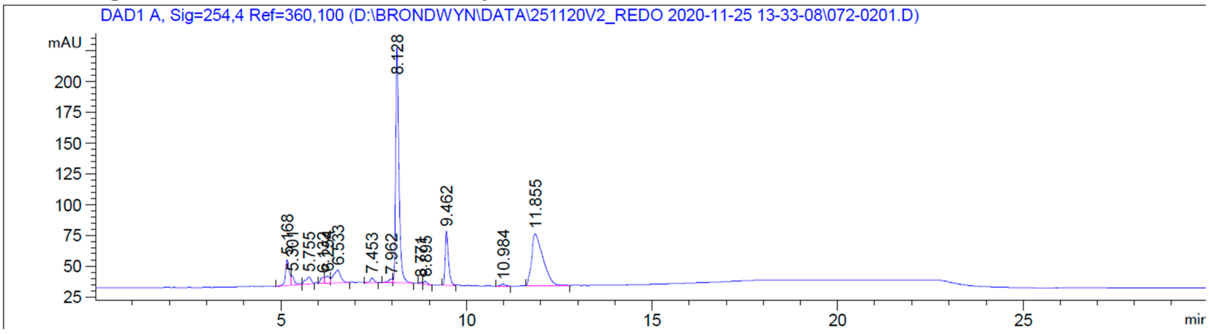

Figure S34. ESIMS spectrum of [Pt(Bequ)(DPQ)]$^+$ measured on a Agilent Technologies 1260 Infinity machine equipped with a Phenomenex Onyx™ Monolithic C18 reverse phase column (100 × 4.6 mm, 130 Å). Sample solutions were made up in H_2O and injected at a 0-100 gradient A to B over 15 minutes with a 15 minute flush in-between samples.

[Pt(Bequ)(BathoPHEN)]$^+$

```
Acq. Operator    : SYSTEM                        Seq. Line :    4
Acq. Instrument  : LC1260                         Location : Vial 74
Injection Date   : 11/25/2020 3:08:39 PM               Inj :    1
                                                Inj Volume : 10.000 µl
Method           : D:\BRONDWYN\DATA\251120V2_REDO 2020-11-25 13-33-08\10 TO 100 OVER 15 MINS
                   10UL.M (Sequence Method)
Last changed     : 11/25/2020 1:33:08 PM by SYSTEM
```

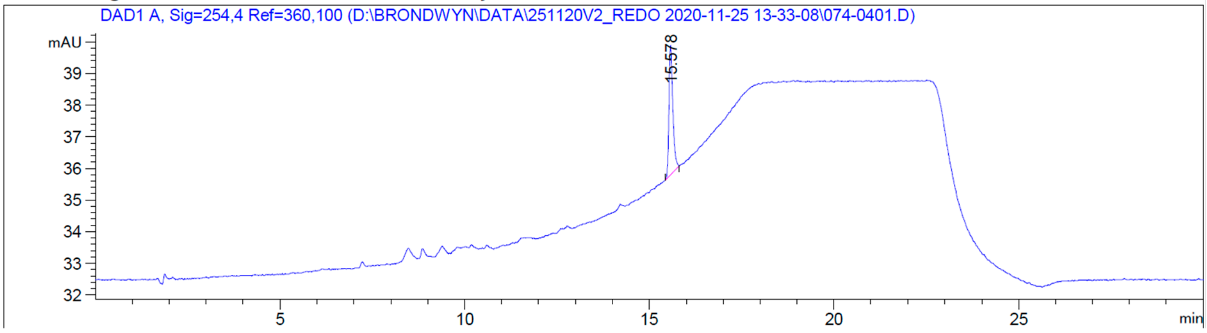

Figure S35. ESIMS spectrum of [Pt(Bequ)(BathoPHEN)]$^+$ measured on a Agilent Technologies 1260 Infinity machine equipped with a Phenomenex Onyx™ Monolithic C18 reverse phase column (100 × 4.6 mm, 130 Å). Sample solutions were made up in H_2O and injected at a 0-100 gradient A to B over 15 minutes with a 15 minute flush in-between samples.

ESI MS

[Pt(Bequ)(BPY)]$^+$

Single Mass Analysis
Tolerance = 10.0 PPM / DBE: min = -1.5, max = 50.0
Element prediction: Off
Number of isotope peaks used for i-FIT = 3

Monoisotopic Mass, Even Electron Ions
6 formula(e) evaluated with 1 results within limits (up to 50 closest results for each mass)
Elements Used:
C: 1-37 H: 1-24 N: 1-3 Pt: 1-1

Sample01
201105_Sample01 49 (0.984) AM2 (Ar,20000.0,556.28,0.00,LS 3); Cm (31:49)
1: TOF MS ES+
1.21e+006

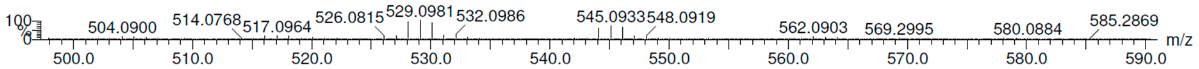

Minimum:				-1.5				
Maximum:		5.0	10.0	50.0				
Mass	Calc. Mass	mDa	PPM	DBE	i-FIT	Norm	Conf(%)	Formula
529.0981	529.0992	-1.1	-2.1	18.5	528.5	n/a	n/a	C23 H16 N3 Pt

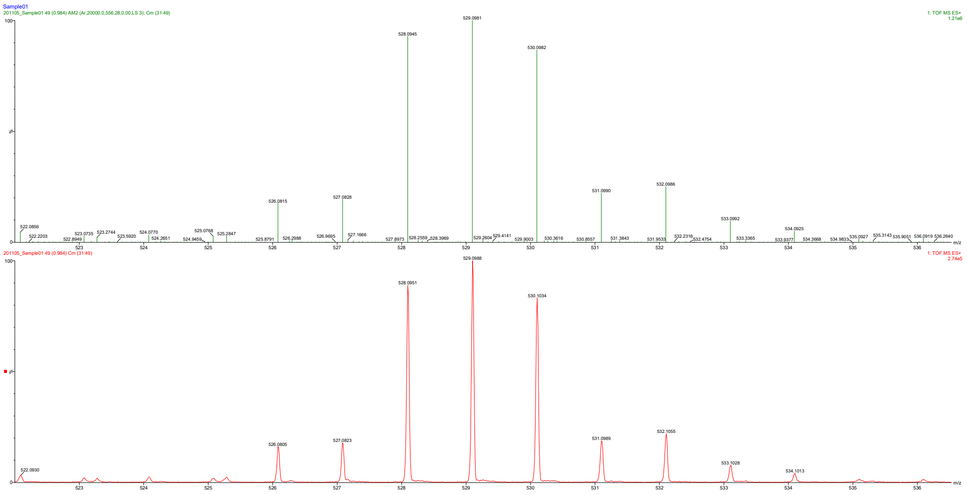

Figure S36. ESIMS spectrum of [Pt(Bequ)(BPY)]$^+$ measured on a Waters TQ-MS triple quadrupole mass spectrometer. Sample solutions were made up to 0.5 mM in H_2O and flowed at 0.1 mL/min.

[Pt(Bequ)(44BPY)]$^+$

Elemental Composition Report

Page 1

Single Mass Analysis

Tolerance = 10.0 PPM / DBE: min = -1.5, max = 50.0
Element prediction: Off
Number of isotope peaks used for i-FIT = 3

Monoisotopic Mass, Even Electron Ions
6 formula(e) evaluated with 1 results within limits (up to 50 closest results for each mass)
Elements Used:
C: 1-37 H: 1-24 N: 1-3 Pt: 1-1

Sample02
201105_Sample02 41 (0.829) AM2 (Ar,20000.0,556.28,0.00,LS 3); Cm (13:49)

1: TOF MS ES+
2.39e+006

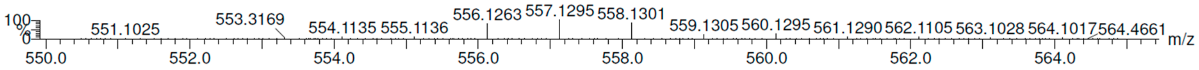

Minimum:				-1.5				
Maximum:		5.0	10.0	50.0				
Mass	Calc. Mass	mDa	PPM	DBE	i-FIT	Norm	Conf(%)	Formula
557.1295	557.1305	-1.0	-1.8	18.5	477.6	n/a	n/a	C25 H20 N3 Pt

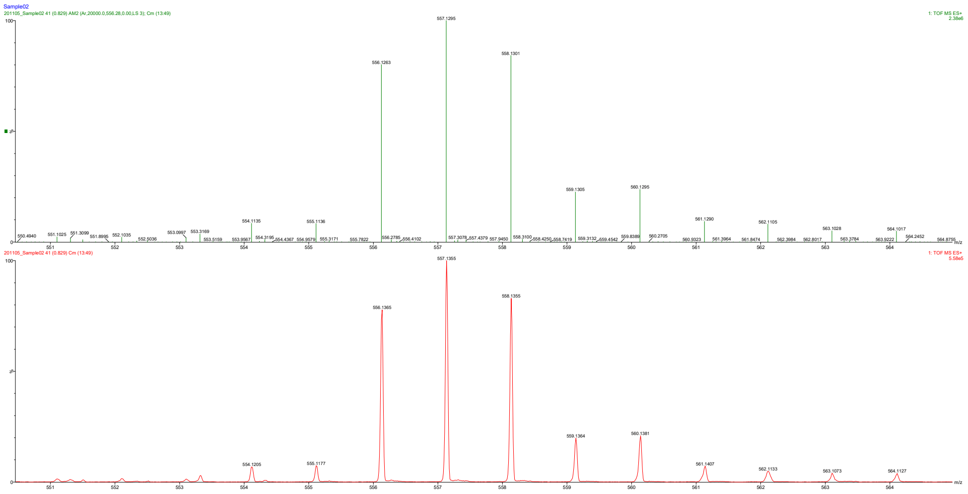

Figure S37. ESIMS spectrum of [Pt(Bequ)(44BPY)]$^+$ measured on a Waters TQ-MS triple quadrupole mass spectrometer. Sample solutions were made up to 0.5 mM in H_2O and flowed at 0.1 mL/min.

[Pt(Bequ)(TertBPY)]+

Elemental Composition Report

Page 1

Single Mass Analysis

Tolerance = 10.0 PPM / DBE: min = -1.5, max = 50.0
Element prediction: Off
Number of isotope peaks used for i-FIT = 3

Monoisotopic Mass, Even Electron Ions
25 formula(e) evaluated with 1 results within limits (up to 50 best isotopic matches for each mass)
Elements Used:
C: 1-37 H: 1-50 N: 1-3 O: 0-1 Pt: 1-1

Sample03
201105_Sample03 41 (0.829) AM2 (Ar,20000.0,556.28,0.00,LS 3); Cm (28:49)

1: TOF MS ES+
5.91e+005

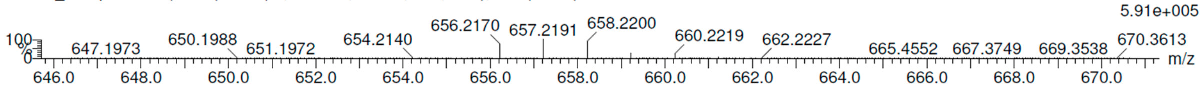

Minimum:				-1.5				
Maximum:		5.0	10.0	50.0				
Mass	Calc. Mass	mDa	PPM	DBE	i-FIT	Norm	Conf(%)	Formula
657.2191	657.2193	-0.2	-0.3	18.5	425.0	n/a	n/a	C31 H32 N3 O Pt

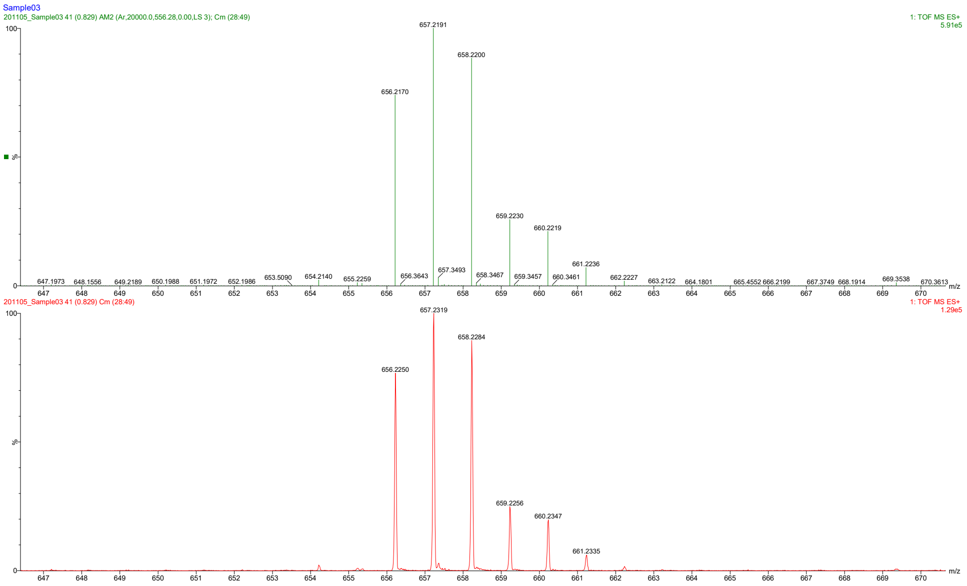

Figure S38. ESIMS spectrum of [Pt(Bequ)(tertBPY)]+ measured on a Waters TQ-MS triple quadrupole mass spectrometer. Sample solutions were made up to 0.5 mM in H_2O and flowed at 0.1 mL/min.

[Pt(Bequ)(PHEN)]+

Elemental Composition Report

Single Mass Analysis

Tolerance = 10.0 PPM / DBE: min = -1.5, max = 50.0
Element prediction: Off
Number of isotope peaks used for i-FIT = 3

Monoisotopic Mass, Even Electron Ions
12 formula(e) evaluated with 1 results within limits (up to 50 best isotopic matches for each mass)
Elements Used:
C: 1-37 H: 1-50 N: 1-3 Pt: 1-1

Sample04
201105_Sample04 5 (0.121) AM2 (Ar,20000.0,556.28,0.00,LS 3); Cm (2:49) 1: TOF MS ES+ 2.24e+006

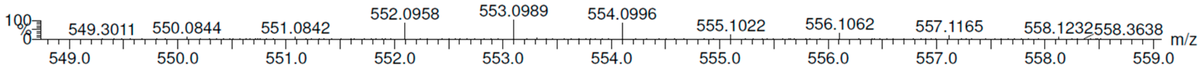

Minimum:				-1.5				
Maximum:		5.0	10.0	50.0				
Mass	Calc. Mass	mDa	PPM	DBE	i-FIT	Norm	Conf(%)	Formula
553.0989	553.0992	-0.3	-0.5	20.5	521.1	n/a	n/a	C25 H16 N3 Pt

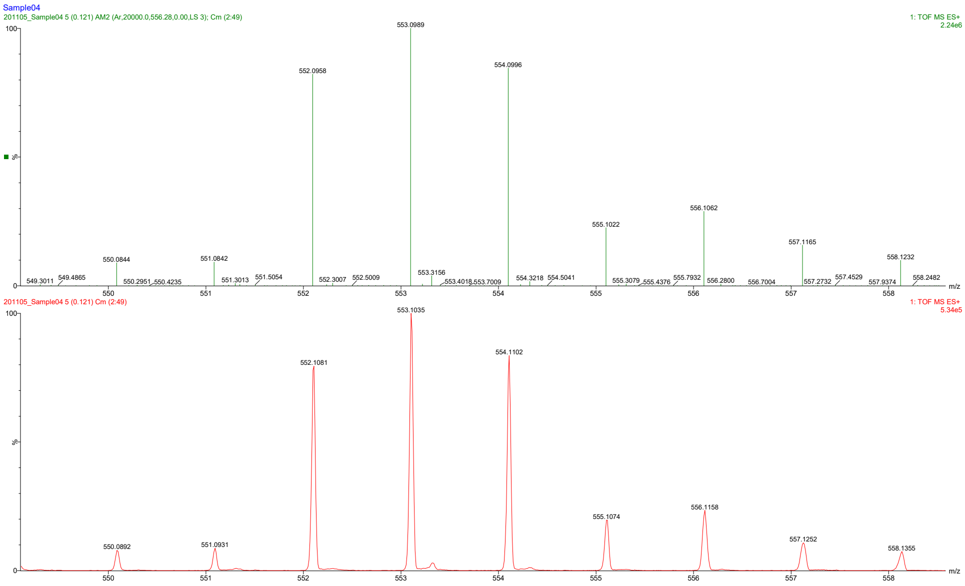

Figure S39. ESIMS spectrum of [Pt(Bequ)(PHEN)]+ measured on a Waters TQ-MS triple quadrupole mass spectrometer. Sample solutions were made up to 0.5 mM in H_2O and flowed at 0.1 mL/min.

[Pt(Bequ)(5MePHEN)]$^+$

Elemental Composition Report **Page 1**

Single Mass Analysis
Tolerance = 10.0 PPM / DBE: min = -1.5, max = 50.0
Element prediction: Off
Number of isotope peaks used for i-FIT = 3

Monoisotopic Mass, Even Electron Ions
25 formula(e) evaluated with 1 results within limits (up to 50 best isotopic matches for each mass)
Elements Used:
C: 1-37 H: 1-50 N: 1-3 O: 0-1 Pt: 1-1

Sample05
201105_Sample05 42 (0.846) AM2 (Ar,20000.0,556.28,0.00,LS 3); Cm (25:49) 1: TOF MS ES+ 1.35e+006

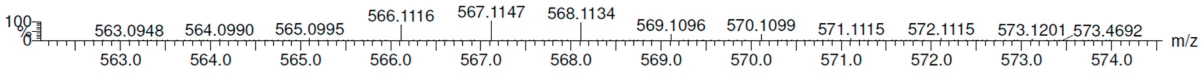

Minimum:				-1.5				
Maximum:		5.0	10.0	50.0				
Mass	Calc. Mass	mDa	PPM	DBE	i-FIT	Norm	Conf(%)	Formula
567.1147	567.1148	-0.1	-0.2	20.5	523.2	n/a	n/a	C26 H18 N3 Pt

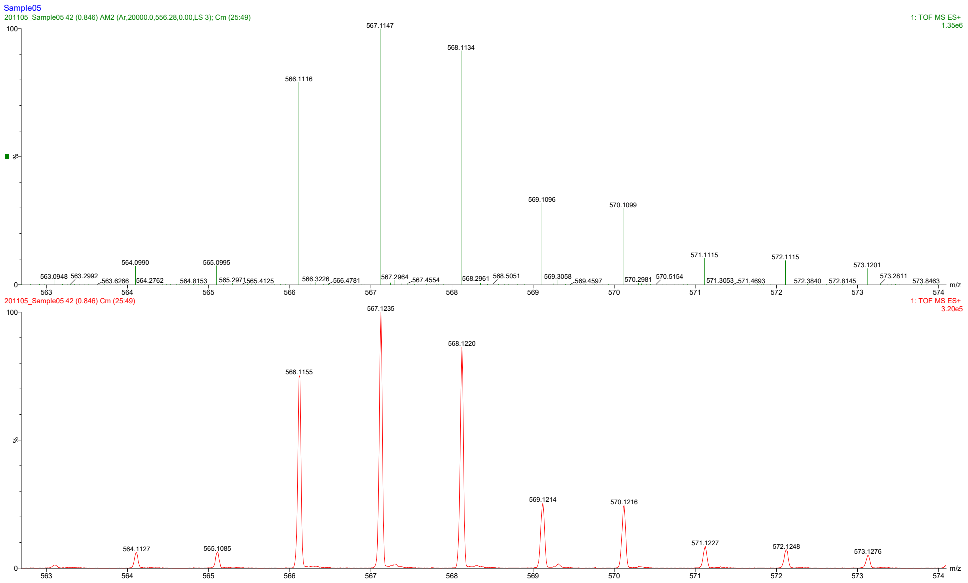

Figure S40. ESIMS spectrum of [Pt(Bequ)(5MePHEN)]$^+$ measured on a Waters TQ-MS triple quadrupole mass spectrometer. Sample solutions were made up to 0.5 mM in H_2O and flowed at 0.1 mL/min.

[Pt(Bequ)(56MePHEN)]$^+$

Elemental Composition Report **Page 1**

Single Mass Analysis
Tolerance = 10.0 PPM / DBE: min = -1.5, max = 50.0
Element prediction: Off
Number of isotope peaks used for i-FIT = 3

Monoisotopic Mass, Even Electron Ions
1 formula(e) evaluated with 1 results within limits (up to 50 best isotopic matches for each mass)
Elements Used:
C: 1-27 H: 1-22 N: 1-3 Pt: 1-1

Sample06a
201105_Sample06a 41 (0.829) AM2 (Ar,20000.0,556.28,0.00,LS 3); Cm (9:44) 1: TOF MS ES+ 4.56e+006

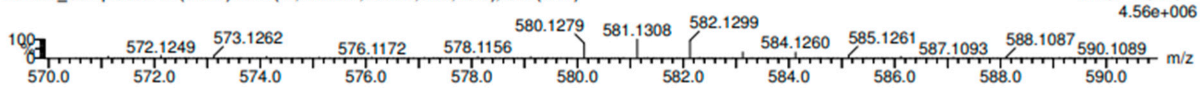

Minimum:				-1.5				
Maximum:		5.0	10.0	50.0				
Mass	Calc. Mass	mDa	PPM	DBE	i-FIT	Norm	Conf(%)	Formula
581.1308	581.1305	0.3	0.5	20.5	553.1	n/a	n/a	C27 H20 N3 Pt

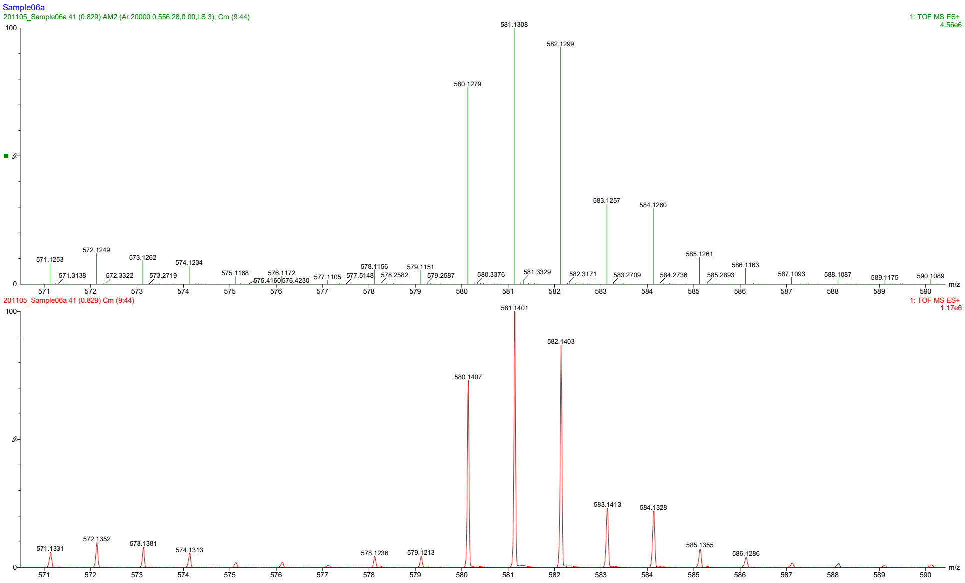

Figure S41. ESIMS spectrum of [Pt(Bequ)(56Me$_2$PHEN)]$^+$ measured on a Waters TQ-MS triple quadrupole mass spectrometer. Sample solutions were made up to 0.5 mM in H_2O and flowed at 0.1 mL/min.

[Pt(Bequ)(TMP)]$^+$

Elemental Composition Report

Single Mass Analysis

Tolerance = 10.0 PPM / DBE: min = -1.5, max = 50.0
Element prediction: Off
Number of isotope peaks used for i-FIT = 3

Monoisotopic Mass, Even Electron Ions
25 formula(e) evaluated with 1 results within limits (up to 50 best isotopic matches for each mass)
Elements Used:
C: 1-37 H: 1-50 N: 1-3 O: 0-1 Pt: 1-1

Sample07
201105_Sample07 33 (0.674) AM2 (Ar,20000.0,556.28,0.00,LS 3); Cm (23:49)
1: TOF MS ES+
1.27e+006

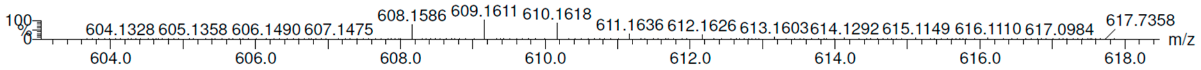

Minimum:				-1.5				
Maximum:		5.0	10.0	50.0				

Mass	Calc. Mass	mDa	PPM	DBE	i-FIT	Norm	Conf(%)	Formula
609.1611	609.1618	-0.7	-1.1	20.5	456.2	n/a	n/a	C29 H24 N3 Pt

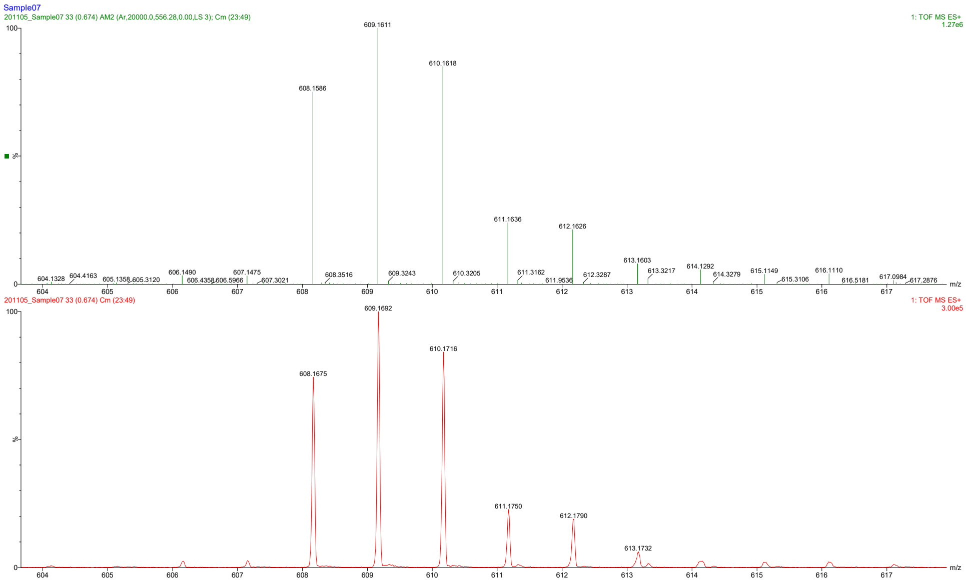

Figure S42. ESIMS spectrum of [Pt(Bequ)(TMP)]$^+$ measured on a Waters TQ-MS triple quadrupole mass spectrometer. Sample solutions were made up to 0.5 mM in H_2O and flowed at 0.1 mL/min.

[Pt(Bequ)(DPQ)]$^{+}$

Elemental Composition Report

Page 1

Multiple Mass Analysis: 2 mass(es) processed

Tolerance = 10.0 PPM / DBE: min = -1.5, max = 50.0
Element prediction: Off
Number of isotope peaks used for i-FIT = 3

Monoisotopic Mass, Odd and Even Electron Ions
11 formula(e) evaluated with 2 results within limits (up to 50 closest results for each mass)
Elements Used:
C: 1-30 H: 1-20 N: 1-5 Pt: 1-1

Sample08
201105_Sample08 45 (0.896) AM2 (Ar,20000.0,556.29,0.00,LS 3); Cm (4:46)

1: TOF MS ES+
3.16e+006

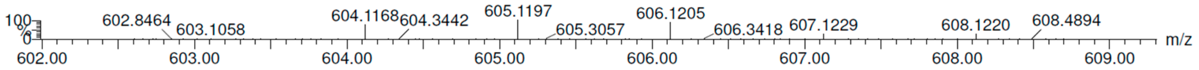

Minimum:	80.00				-1.5				
Maximum:	100.00		5.0	10.0	50.0				
Mass	RA	Calc. Mass	mDa	PPM	DBE	i-FIT	Norm	Conf(%)	Formula
605.1197	100.00	605.1179	1.8	3.0	23.0	562.4	n/a	n/a	C28 H18 N4 Pt
606.1205	86.70	606.1257	-5.2	-8.6	22.5	566.6	n/a	n/a	C28 H19 N4 Pt

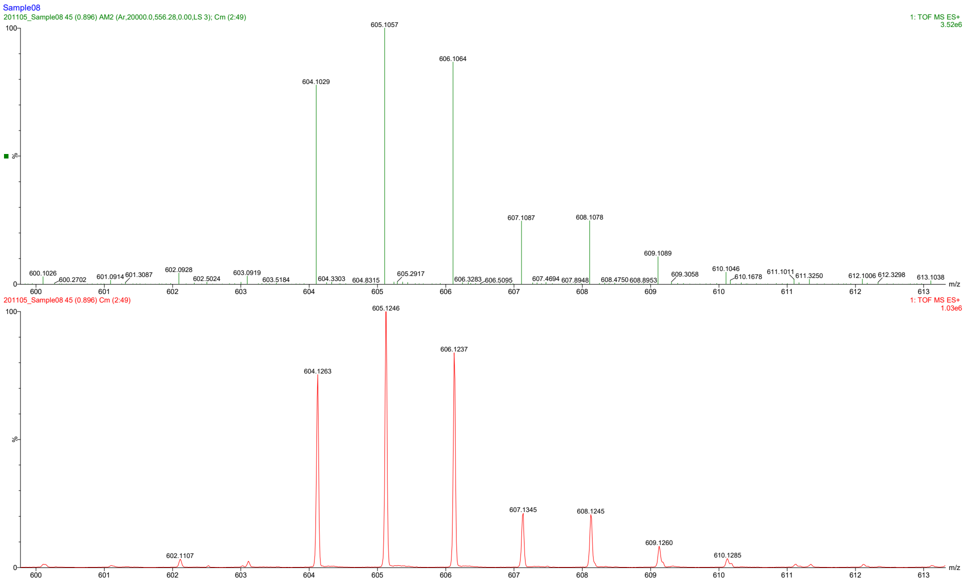

Figure S43. ESIMS spectrum of [Pt(Bequ)(DPQ)]$^{+}$ measured on a Waters TQ-MS triple quadrupole mass spectrometer. Sample solutions were made up to 0.5 mM in H_2O and flowed at 0.1 mL/min.

[Pt(Bequ)(BathoPHEN)]$^{+}$

Elemental Composition Report

Multiple Mass Analysis: 2 mass(es) processed

Tolerance = 10.0 PPM / DBE: min = -1.5, max = 50.0
Element prediction: Off
Number of isotope peaks used for i-FIT = 3

Monoisotopic Mass, Odd and Even Electron Ions
1 formula(e) evaluated with 0 results within limits (up to 50 closest results for each mass)
Elements Used:
C: 1-37 H: 1-24 N: 1-3 Pt: 1-1

Sample09
201105_Sample09 38 (0.758) AM2 (Ar,20000.0,556.29,0.00,LS 3); Cm (4:40)
1: TOF MS ES+
4.93e+005

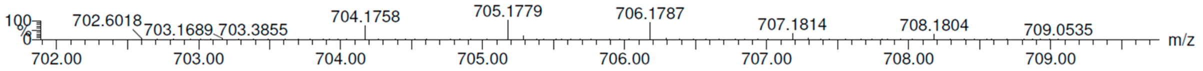

Minimum:	80.00				-1.5				
Maximum:	100.00		5.0	10.0	50.0				
Mass	RA	Calc. Mass	mDa	PPM	DBE	i-FIT	Norm	Conf(%)	Formula
705.1779	100.00	---							
706.1787	89.53	---							

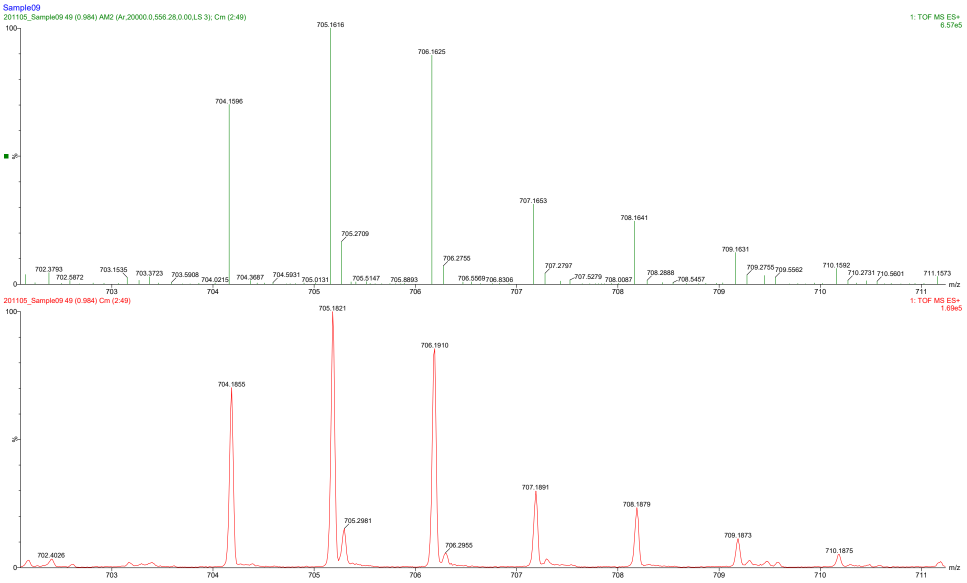

Figure S44. ESIMS spectrum of [Pt(Bequ)(BathoPHEN)]$^{+}$ measured on a Waters TQ-MS triple quadrupole mass spectrometer. Sample solutions were made up to 0.5 mM in H_2O and flowed at 0.1 mL/min.

Fluorescence

QY

Quantum yields were obtained by following the IUPAC technical report on standards for photoluminescence quantum yield measurements in solution.[1] $[Ru(bpy)_3]^{2+}$ was chosen as the best standard as per these guidelines and the quantum yield of the 9 complexes was calculated using Equation 3 following the steps from the technical report.

Equation S3

$$\Phi_{st} = \Phi_s \frac{slope_{st} n_{st}^2}{slope_s \, n_s^2}$$

Where Φ_{st} and Φ_s is the quantum yield of the standard and the sample respectfully, n_{st} and n_s are the refractive indices of the solvents that the standard and sample are dissolved in respectively, and $slope_{st}$ and $slope_s$ are the slope of the line obtained from the plot of the area of fluorescence vs. absorbance of the standard and the sample respectfully.

1. A. M. Brouwer, *Pure & Applied Chemistry*, 2011, **83**.

Fluorescent Titrations

$[Pt(Bequ)(BPY)]^+$

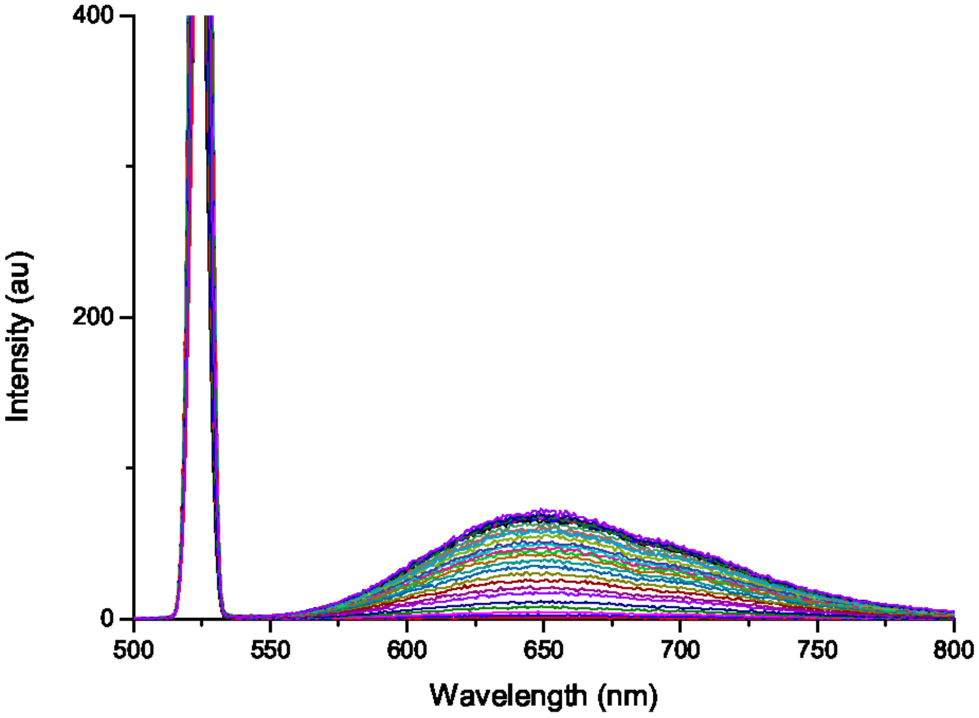

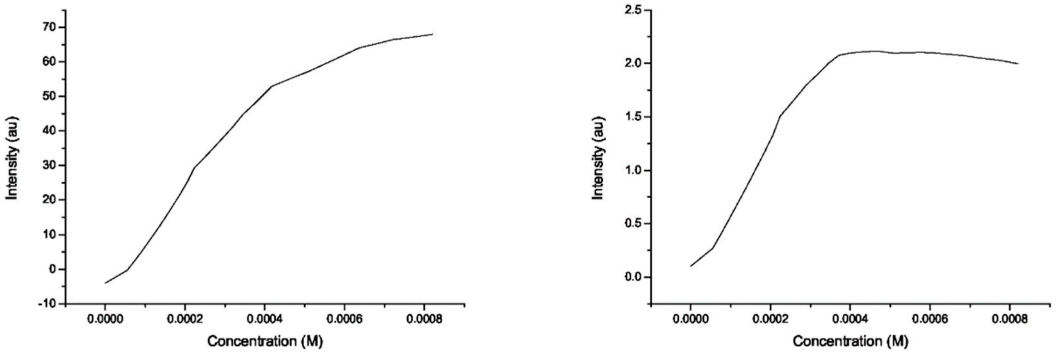

Figure S45. Concentration to fluorescence relationship investigated by titrating 1.1 mM solution of [Pt(Bequ)(BPY)]$^+$ into 400 µL H_2O Excitation at Em_{max} and emission measured between 500-800 nm above, bellow; fluorescence intensity of [Pt(Bequ)(BPY)]$^+$ at 650 (right) and 541 (left) nm as concentration increases.

[Pt(Bequ)(44BPY)]$^+$

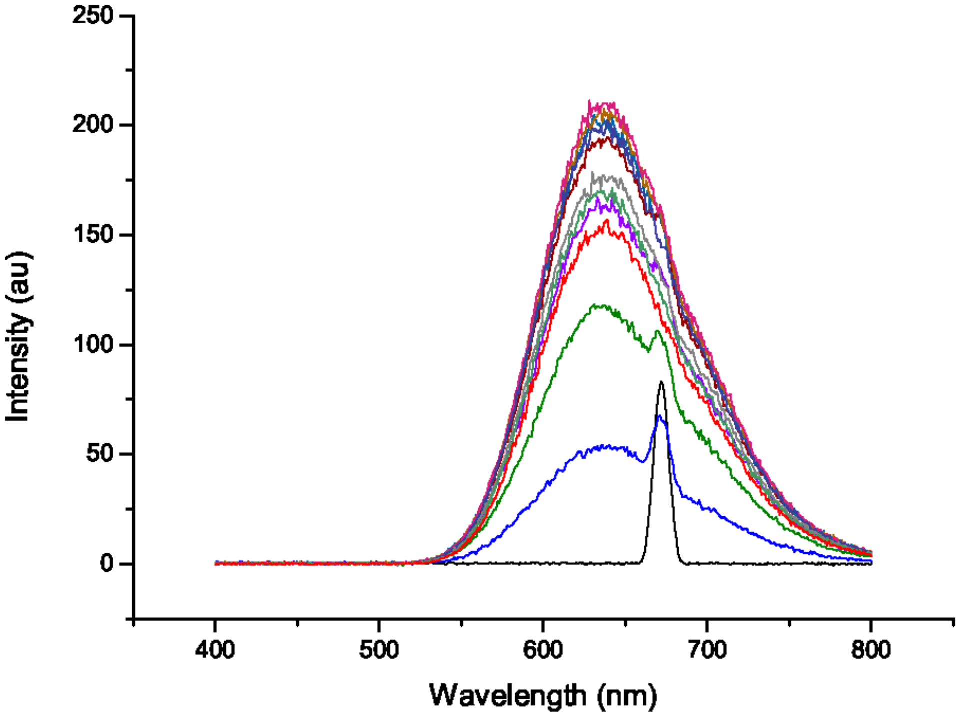

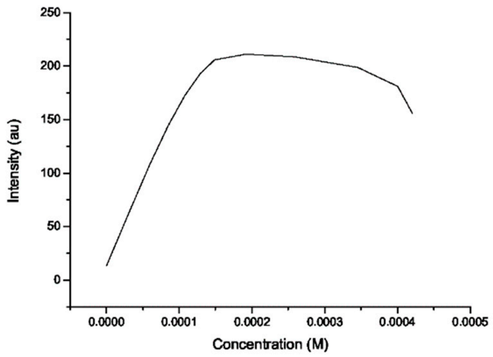

Figure S46. Above; Concentration to fluorescence relationship investigated by titrating 0.64 mM solutions of [Pt(Bequ)(44BPY)]$^+$ into 400 µL H_2O Excitation at Em_{max} and emission measured between 400-800 nm and below; fluorescence intensity of [Pt(Bequ)(44BPY)]$^+$ at 641 nm as concentration increases.

[Pt(Bequ)(TertBPY)]$^+$

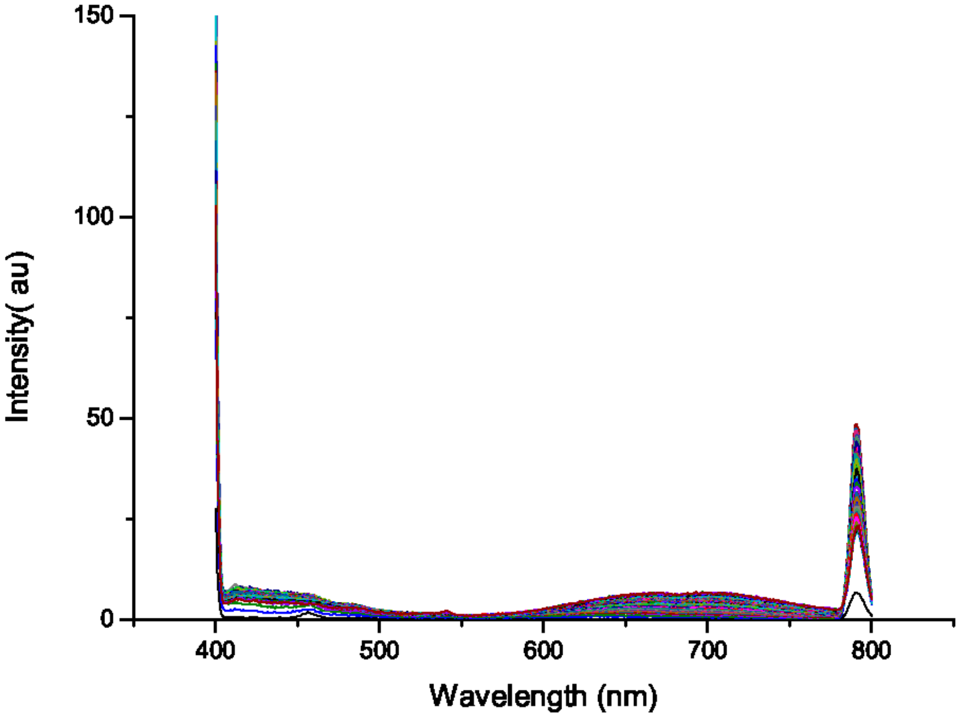

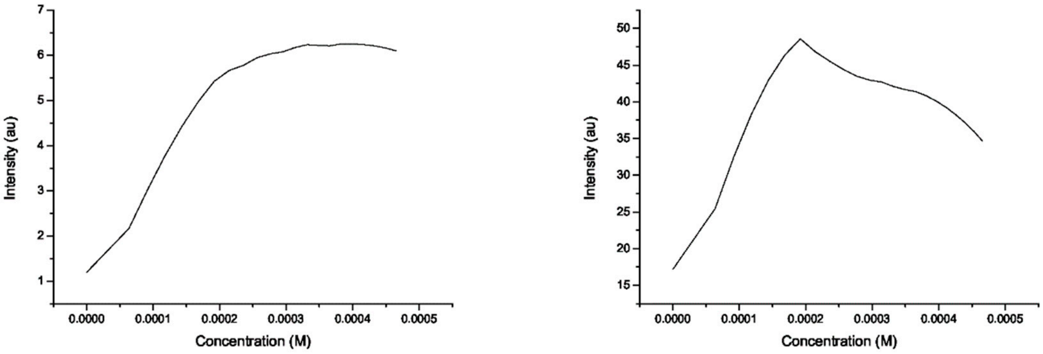

Figure S47. Above, concentration to fluorescence relationship investigated by titrating 1.27 mM solutions of [Pt(Bequ)(44BPY)]$^+$ into 400 µL H_2O Excitation at Em_{max} and emission measured between 400-800 nm and bellow; fluorescence intensity of [Pt(Bequ)(tertBPY)]$^+$ at 465 (right) and 790 (left) nm as concentration increases.

[Pt(Bequ)(PHEN)]$^+$

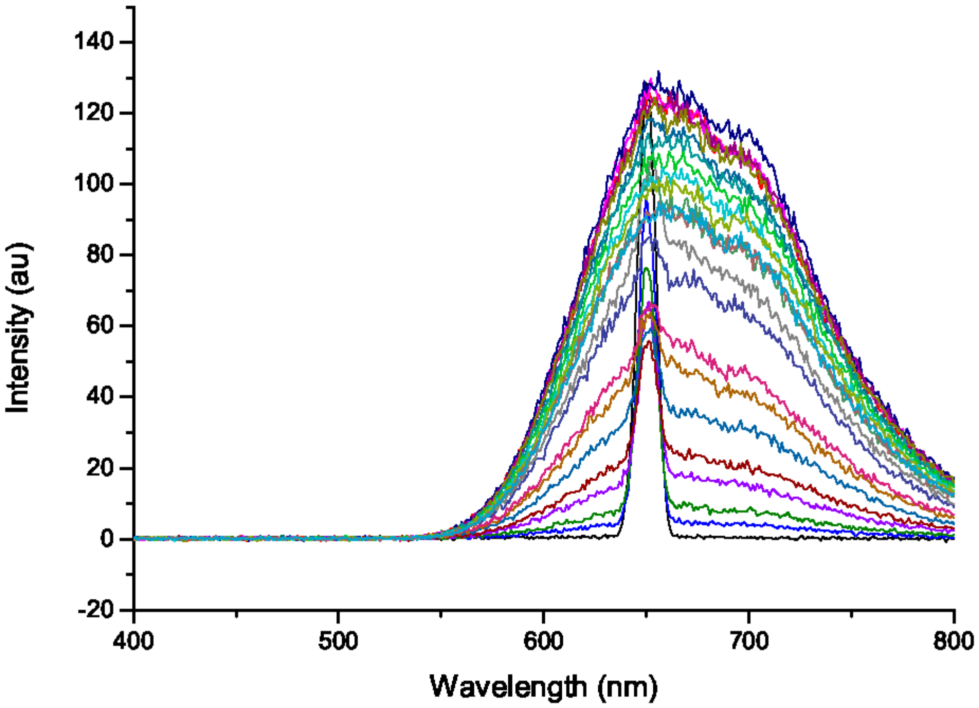

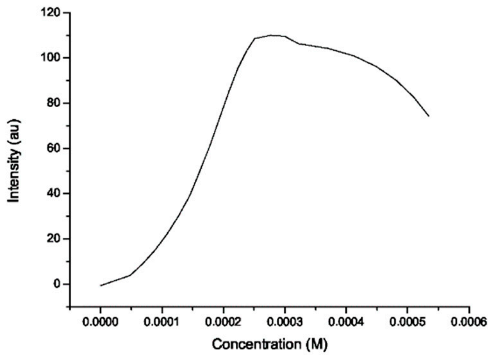

Figure S48. Above; concentration to fluorescence relationship investigated by titrating 0.96 mM solutions of $[Pt(Bequ)(PHEN)]^+$ into 400 µL H_2O Excitation at Em_{max} and emission measured between 400-800 nm and bellow; fluorescence intensity of $[Pt(Bequ)(PHEN)]^+$ at 700 nm as concentration increases.

[Pt(Bequ)(5MePHEN)]$^{+}$

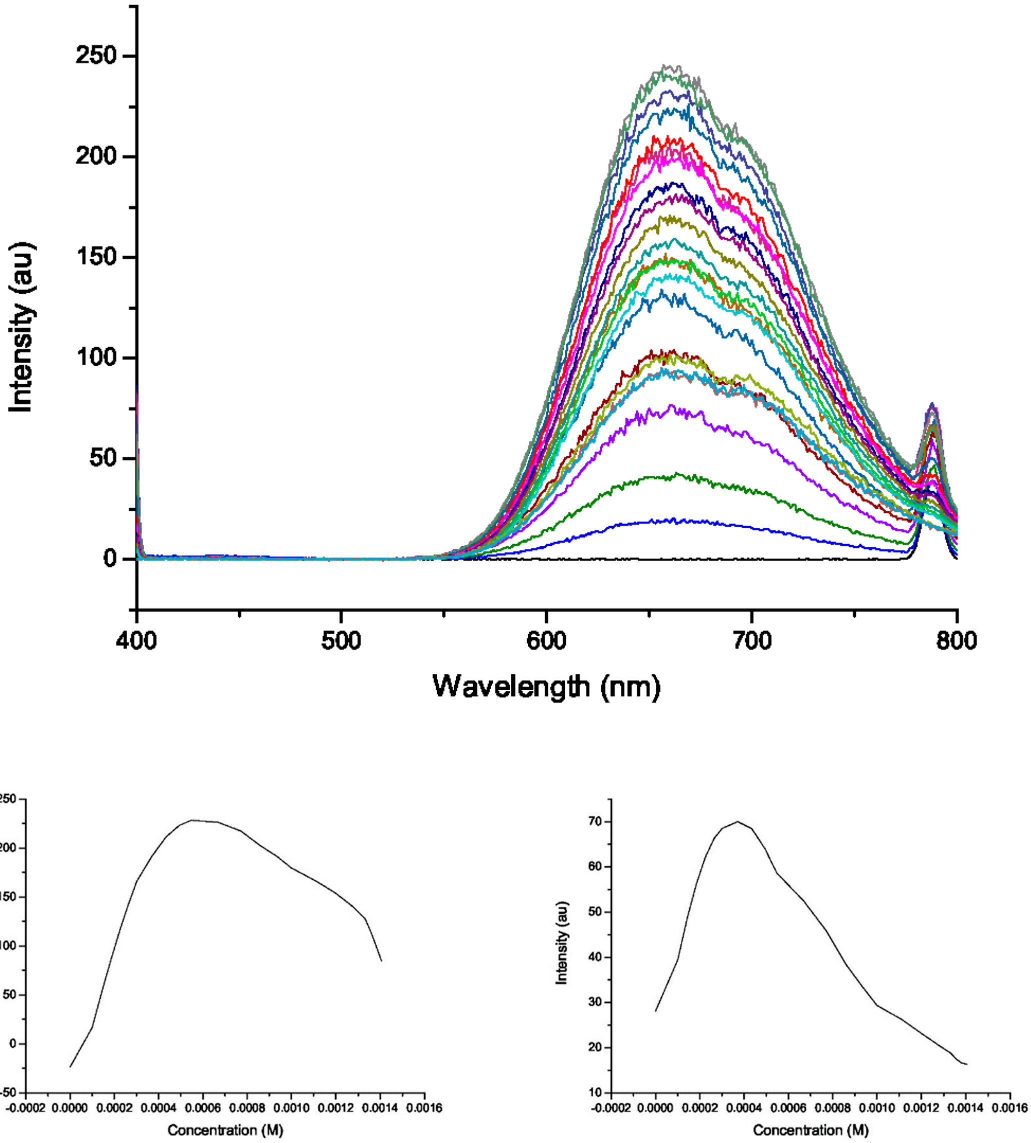

Figure S49. Above; concentration to fluorescence relationship investigated by titrating 1.19 mM solutions of [Pt(Bequ)(5MePHEN)]$^{+}$ into 400 µL H_2O Excitation at Em_{max} and emission measured between 400-800 nm and bellow; fluorescence intensity of [Pt(Bequ)(5MePHEN)]$^{+}$ at 666 (right) and 790 (left) nm as concentration increases.

[Pt(Bequ)(56MePHEN)]+

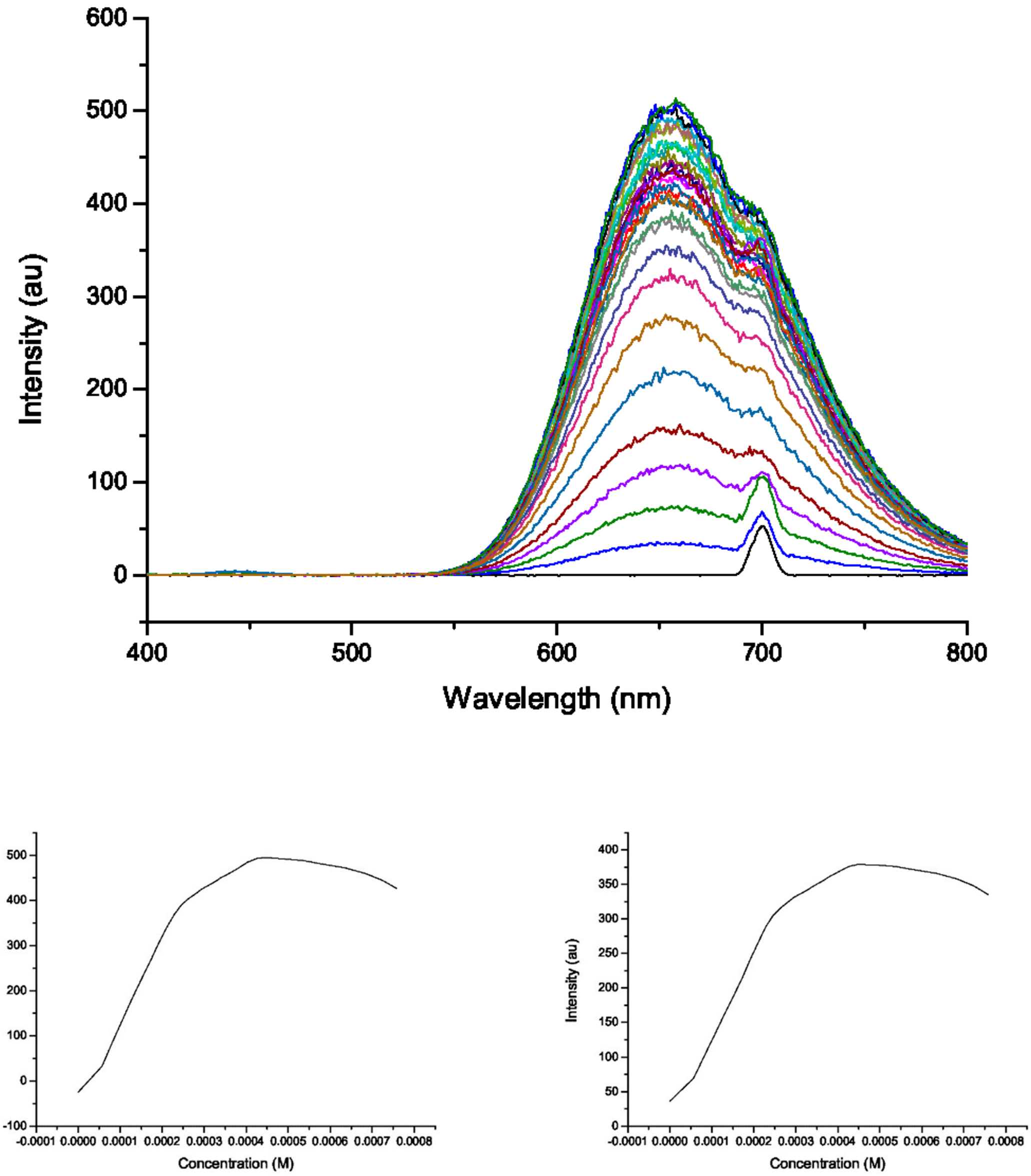

Figure S50. Above, concentration to fluorescence relationship investigated by titrating 1.1 mM solutions of $[Pt(Bequ)(56Me_2PHEN)]^+$ into 400 µL H_2O Excitation at Em_{max} and emission measured between 400-800 nm and bellow; fluorescence intensity of $[Pt(Bequ)(56Me_2PHEN)]^+$ at 657 (right) and 700 (left) nm as concentration increases.

[Pt(Bequ)(TMP)]$^+$

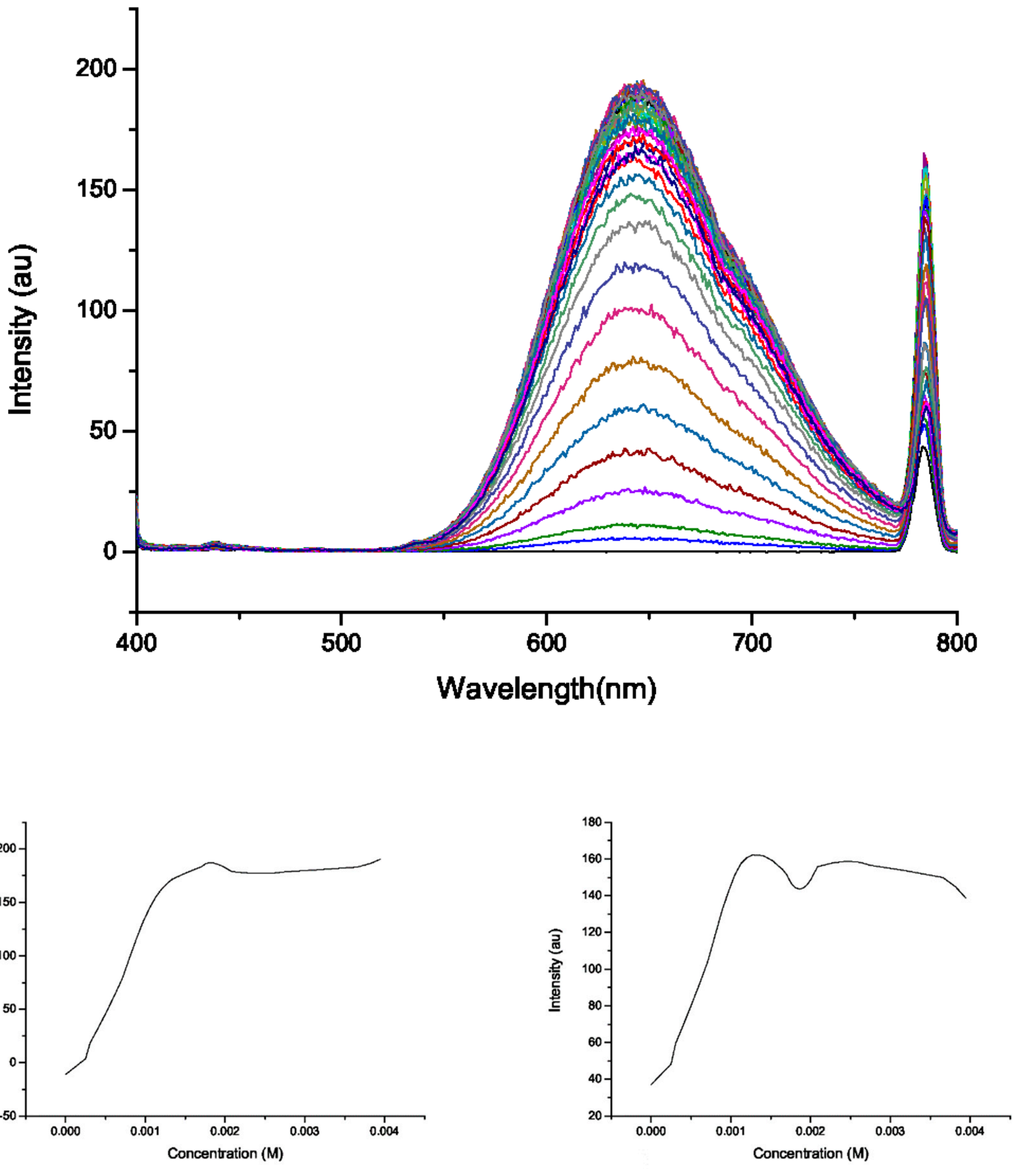

Figure S51. Above; concentration to fluorescence relationship investigated by titrating 5.0 mM solutions of [Pt(Bequ)(TMP)]$^+$ into 400 µL H_2O Excitation at Em_{max} and emission measured between 400-800 nm and bellow; fluorescence intensity of [Pt(Bequ)(TMP)]$^+$ at 645 (right) and 785 (left) nm as concentration increases.

$[Pt(Bequ)(DPQ)]^+$

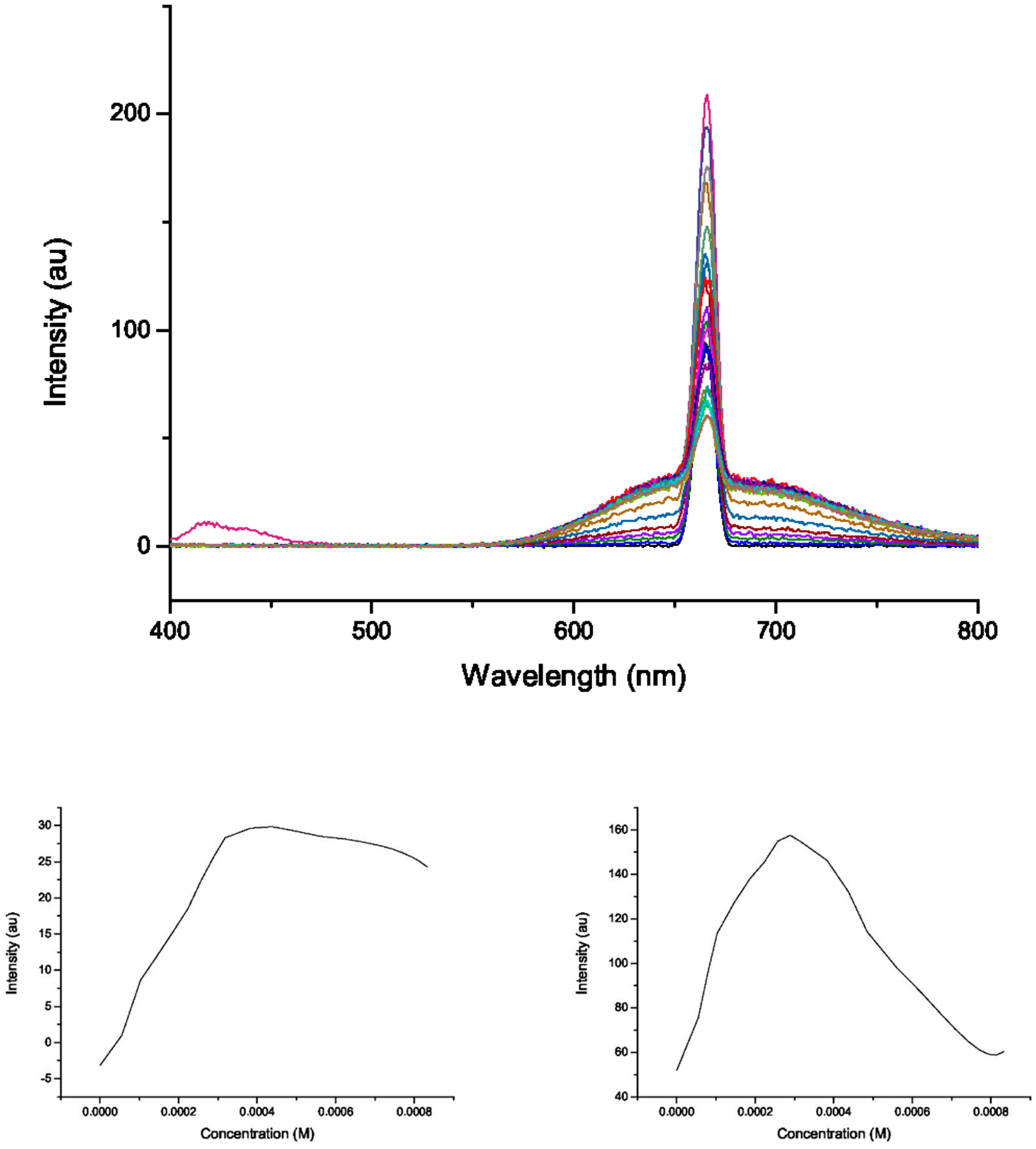

Figure S52. Above; concentration to fluorescence relationship investigated by titrating 1.1 mM solutions of $[Pt(Bequ)(DPQ)]^+$ into 400 µL H_2O Excitation at Em_{max} and emission measured between 400-800 nm and bellow; fluorescence intensity of $[Pt(Bequ)(DPQ)]^+$ at 522 (right) and 790 (left) nm as concentration increases.

[Pt(Bequ)(BathoPHEN)]$^+$

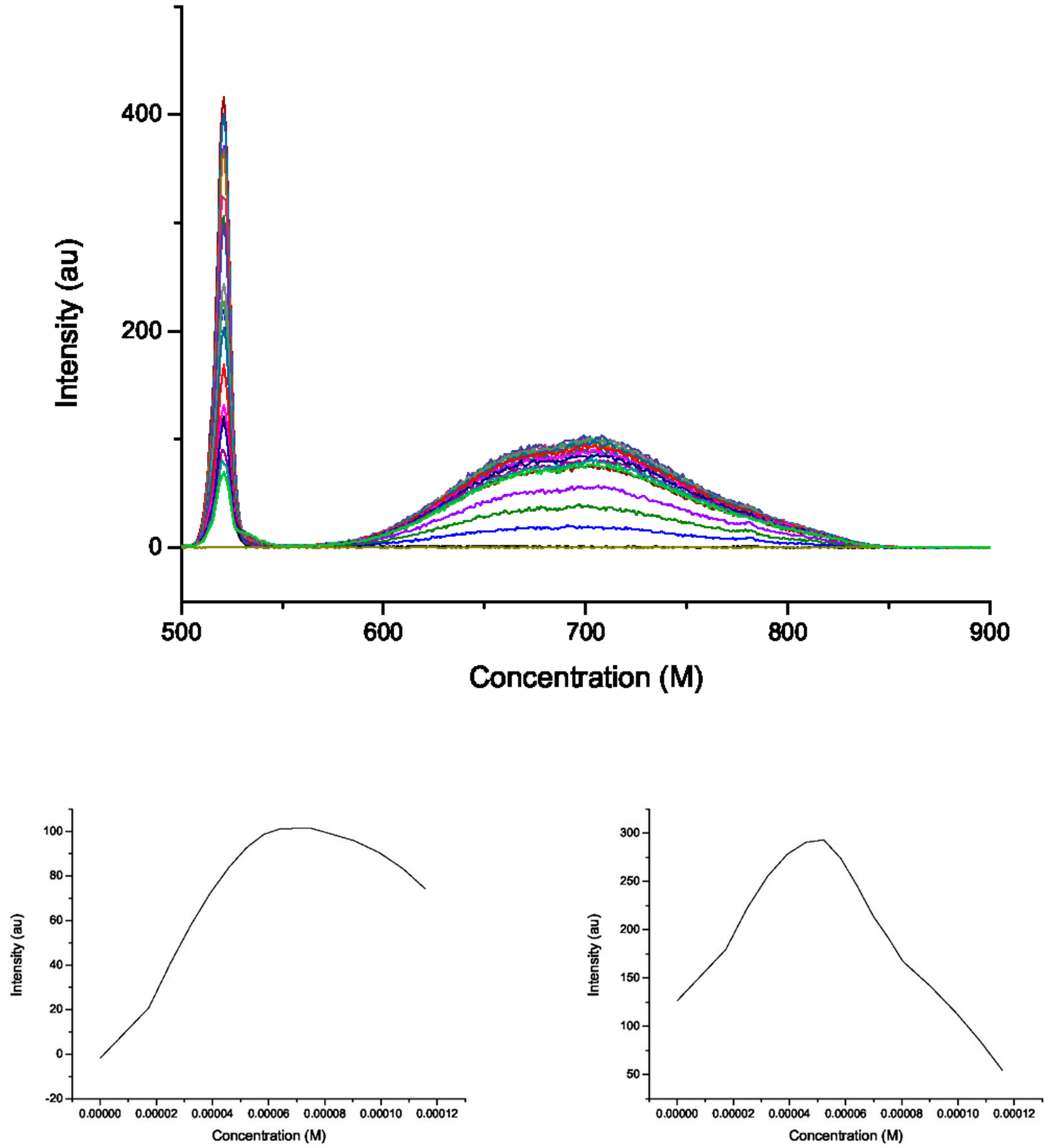

Figure S53. Above; concentration to fluorescence relationship investigated by titrating 0.34 mM solutions of [Pt(Bequ)(BathoPHEN)]$^+$ into 400 µL H_2O Excitation at Em_{max} and emission measured between 500-800 nm and bellow; fluorescence intensity of [Pt(Bequ)(TMP)]$^+$ at 706 (right) and 522 (left) nm as concentration increases.

DNA Melts

Table S3. melting temperature od C-MYC and H-TELO QDNA annealed with and without MCs 1-9 from 0 up to 6 molar equivalents.

		MC equiv.	[Pt(BPY)(Bequ)]$^{+}$	[Pt(44BPY)(Bequ)]$^{+}$	[Pt(tertBPY)(Bequ)]$^{+}$	[Pt(Phen)(Bequ)]$^{+}$	[Pt(5MePhen)(Bequ)]$^{+}$	[Pt(56Me2Phen)(Bequ)]$^{+}$	[Pt(TMP)(Bequ)]$^{+}$	[Pt(DPQ)(Bequ)]$^{+}$	[Pt(Batho)(Bequ)]$^{+}$
C-MYC	Annealed w/o MC	0	70.02 ± 1.28	70.02 ± 1.28	70.02 ± 1.28	70.02 ± 1.28	70.02 ± 1.28	70.02 ± 1.28	70.02 ± 1.28	70.02 ± 1.28	70.02 ± 1.28
		1	70.09 ± 1.23	74.84 ± 0.54	69.15 ± 1.93	71.09 ± 1.03	70.09 ± 1.23	69.69 ± 1.18	70.96 ± 0.14	70.11 ± 1.89	69.93 ± 0.74
		2	72.05 ± 0.19	75.30 ± 1.99	70.74 ± 0.90	73.64 ± 1.12	71.20 ± 1.96	70.78 ± 1.49	71.40 ± 0.79	70.34 ± 0.28	72.63 ± 1.98
		3	72.11 ± 1.96	76.37 ± 0.34	72.77 ± 1.99	75.02 ± 1.76	74.57 ± 1.23	71.58 ± 1.31	72.99 ± 0.73	70.87 ± 0.07	73.77 ± 1.69
		4	76.51 ± 1.85	76.39 ± 1.17	73.62 ± 0.91	76.67 ± 1.10	76.93 ± 1.80	71.92 ± 1.13	73.97 ± 1.58	71.58 ± 1.54	76.06 ± 0.44
		5	78.14 ± 1.41	76.76 ± 1.08	74.37 ± 0.77	79.86 ± 1.13	80.03 ± 1.03	76.44 ± 1.63	79.87 ± 1.00	74.76 ± 0.22	76.63 ± 0.96
		6	79.62 ± 1.36	78.14 ± 1.98	83.94 ± 1.91	81.83 ± 1.23	82.88 ± 0.16	76.69 ± 1.74	84.48 ± 1.14	80.17 ± 0.67	■
	Annealed w MC	0	70.02 ± 1.28	70.02 ± 1.28	70.02 ± 1.28	70.02 ± 1.28	70.02 ± 1.28	70.02 ± 1.28	70.02 ± 1.28	70.02 ± 1.28	70.02 ± 1.28
		1	69.59 ± 1.45	70.29 ± 1.18	68.63 ± 0.11	71.16 ± 0.11	69.41 ± 1.52	70.60 ± 1.33	72.97 ± 1.14	66.52 ± 1.72	67.20 ± 1.12
		2	74.24 ± 1.44	70.73 ± 0.13	69.41 ± 0.64	71.69 ± 0.64	70.45 ± 0.49	70.84 ± 1.78	73.79 ± 1.40	70.36 ± 0.30	71.11 ± 1.73
		3	74.83 ± 0.30	71.20 ± 0.73	69.80 ± 0.24	73.12 ± 0.24	72.23 ± 1.66	70.95 ± 0.81	73.94 ± 0.54	71.87 ± 1.27	71.70 ± 1.56
		4	78.02 ± 1.47	73.81 ± 0.41	70.33 ± 0.08	73.33 ± 0.08	72.85 ± 1.10	71.73 ± 0.46	74.44 ± 0.75	72.22 ± 1.57	72.22 ± 1.26
		5	78.13 ± 1.99	74.39 ± 0.54	72.63 ± 1.96	73.65 ± 1.96	73.57 ± 1.05	74.18 ± 0.98	74.85 ± 0.35	73.74 ± 0.63	72.95 ± 0.75
		6	85.50 ± 0.37	79.33 ± 1.37	72.72 ± 0.14	78.73 ± 0.14	80.95 ± 2.00	75.07 ± 0.71	75.40 ± 1.49	73.85 ± 1.51	■
H-TELO	Annealed w/o MC	0	65.87 ± 2.01	65.87 ± 2.01	65.87 ± 2.01	65.87 ± 2.01	65.87 ± 2.01	65.87 ± 2.01	65.87 ± 2.01	65.87 ± 2.01	65.87 ± 2.01
		1	71.37 ± 1.83	71.28 ± 1.90	70.88 ± 0.30	70.00 ± 1.80	75.27 ± 0.14	65.89 ± 0.77	69.34 ± 0.29	69.16 ± 0.15	73.57 ± 0.60
		2	72.18 ± 1.10	74.83 ± 1.06	77.00 ± 0.53	71.50 ± 0.01	76.44 ± 0.13	70.62 ± 0.53	70.55 ± 1.95	72.26 ± 0.25	73.93 ± 0.02
		3	73.92 ± 0.20	75.30 ± 0.75	■	77.03 ± 1.53	81.29 ± 1.69	70.85 ± 1.96	71.22 ± 0.07	73.57 ± 1.57	74.03 ± 0.56
		4	74.19 ± 0.24	75.34 ± 0.95	■	78.75 ± 1.86	82.41 ± 1.55	71.31 ± 0.69	71.46 ± 1.83	73.72 ± 1.80	74.29 ± 0.34
		5	79.05 ± 1.23	75.65 ± 1.13	■	81.45 ± 1.77	■	77.03 ± 0.53	72.98 ± 1.09	74.08 ± 1.12	78.11 ± 1.48
		6	85.36 ± 0.15	76.06 ± 0.55	■	■	■	79.55 ± 0.65	81.25 ± 0.52	81.28 ± 2.00	79.55 ± 1.57
	Annealed w MC	0	65.87 ± 2.01	65.87 ± 2.01	65.87 ± 2.01	65.87 ± 2.01	65.87 ± 2.01	65.87 ± 2.01	65.87 ± 2.01	65.87 ± 2.01	65.87 ± 2.01
		1	70.27 ± 1.05	71.60 ± 0.19	68.33 ± 0.86	66.88 ± 0.86	66.93 ± 1.37	72.42 ± 1.68	67.91 ± 1.44	70.46 ± 0.55	68.99 ± 1.10
		2	70.70 ± 1.38	72.30 ± 1.23	68.88 ± 1.01	68.42 ± 1.01	70.58 ± 1.59	72.72 ± 0.50	68.22 ± 0.06	71.47 ± 0.56	69.83 ± 0.90
		3	71.94 ± 1.36	73.26 ± 0.06	69.66 ± 0.50	69.78 ± 0.50	73.04 ± 0.32	72.83 ± 0.06	69.86 ± 1.40	71.89 ± 0.89	70.17 ± 0.55
		4	72.63 ± 1.39	78.73 ± 1.78	71.68 ± 1.26	70.21 ± 1.26	74.20 ± 0.18	72.97 ± 1.17	70.15 ± 0.91	72.50 ± 0.06	72.10 ± 1.76
		5	74.67 ± 1.73	82.37 ± 0.75	74.22 ± 0.86	70.82 ± 0.86	75.43 ± 0.58	73.26 ± 0.80	71.27 ± 0.28	72.51 ± 1.04	73.00 ± 0.82
		6	75.08 ± 1.99	■	83.07 ± 0.79	74.70 ± 0.79	77.39 ± 0.89	■	73.25 ± 0.28	77.77 ± 1.80	76.37 ± 0.41

■ Indicates Melt data did not obtain a melting curve in any of the triplicates –all other melts are the average between all three replicates.

Table S4. Change in melting temperature (ΔMT) from) equivalents of MC to 6 equivalents of MC annealed both with and without the presence of the metal complex.

	ΔMT 0-6*			
	Annealed w/o MC		Annealed wMC	
	Cmyc	H-telo	Cmyc	H-telo
**[Pt(Bequ)(BPY)]+**	9.6	19.49	15.48	9.21
**[Pt(Bequ)(44BPY)]+**	8.12	10.19	9.31	16.5
**[Pt(Bequ)(TertBPY)]+**	13.92	11.13	2.7	17.2
**[Pt(Bequ)(PHEN)]+**	11.81	15.58	8.71	8.83
**[Pt(Bequ)(5MePHEN)]+**	12.86	16.54	10.93	11.52
**[Pt(Bequ)(56MePHEN)]+**	6.67	13.68	5.05	7.39
**[Pt(Bequ)(TMP)]+**	14.46	15.38	5.38	7.38
**[Pt(Bequ)(DPQ)]+**	10.15	15.41	3.83	11.9
**[Pt(Bequ)(BathoPHEN)]+**	6.61	13.68	2.93	10.5

Lipophilicity

Lipophilicity was calculated using RP-HPLC using a standard method whereby a stock solution was injected at different isocratic ratios ranging from 70-90 % solvent B (organic) at a flow rate of 1 $mLmin^{-1}$[1–3]. The subsequent peaks were recorded, and K was calculated as per Equation S4.

Equation S4

$$k = \frac{t_r - t_0}{t_0}$$

Where k is the capacity factor, t_r is the retention time of the analyte and t_0 is the dead time.

For each compound the stock solution was run at 5 different isocratic ratios each repeated 3 times. Logk' was then calculated and plotted against the concentration of ACN in the mobile phase. The resulting linier equations could then be used to calculate log kw, expressed by Equation S5.

Equation S5

$$\log k = S\varphi + \log k_w$$

Where S is the slope, φ is the concentration of ACN in the mobile phase and log k_w represents the capacity factor of the compound in 100% water.

Figure S1 illustrates the linear plots of logK' against % ACN including the resulting equation. These experiments were undertaken on an Agilent Technologies 1260 Infinity machine equipped with a Phenomenex Onyx™ Monolithic C18 reverse phase column (100 × 4.6 mm, 130 Å). The mobile phase comprised of 0.06% TFA in water (solvent A) and 0.06% TFA in ACN.H_2O (90 . 10, solvent B). The dead time was determined using potassium iodide as an external dead volume marker.

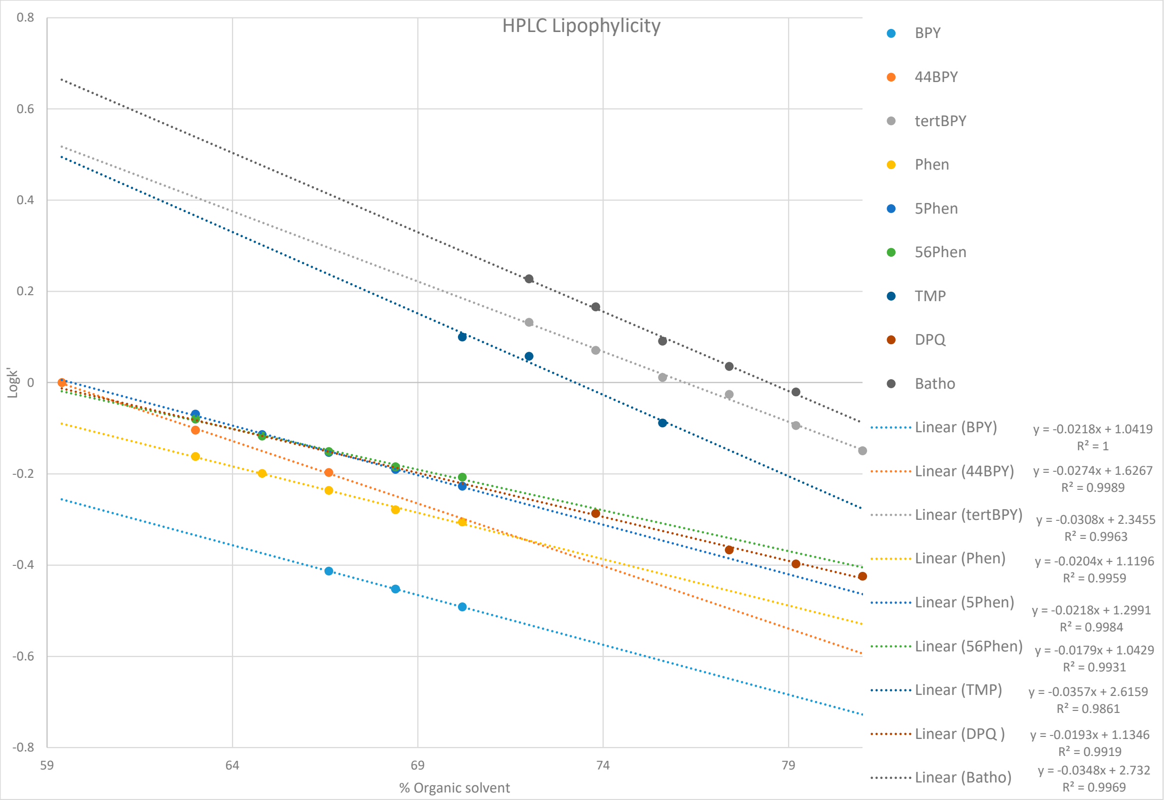

Figure S54. LogK' plotted against % ACN in the mobile phase.

1. Klose, M.H.M.; Theiner, S.; Varbanov, H.P.; Hoefer, D.; Pichler, V.; Galanski, M.; Meier-Menches, S.M.; Keppler, B.K. Development and Validation of Liquid Chromatography-Based Methods to Assess the Lipophilicity of Cytotoxic Platinum(IV) Complexes. *Inorganics* **2018**, *6*, 130, doi.10.3390/inorganics6040130.
2. Reithofer, M.R.; Bytzek, A.K.; Valiahdi, S.M.; Kowol, C.R.; Groessl, M.; Hartinger, C.G.; Jakupec, M.A.; Galanski, M.; Keppler, B.K. Tuning of Lipophilicity and Cytotoxic Potency by Structural Variation of Anticancer Platinum(IV) Complexes. *J. Inorg. Biochem.* **2011**, *105*, 46–51, doi.10.1016/j.jinorgbio.2010.09.006.
3. Valkó, K. Application of High-Performance Liquid Chromatography Based Measurements of Lipophilicity to Model Biological Distribution. *J. Chromatogr. A* **2004**, *1037*, 299–310, doi.10.1016/j.chroma.2003.10.084.

Correlation analysis

To investigate correlation between MC physical and biological properties the MC's were split into three groups. These were the "BPY" complexes 1-3, the "PHEN" complexes 4-6 and the "Large" complexes 7-9. We then identified three main categories of biophysical characterisation, size (including lipophilicity), DNA stabilising ability in terms of change in temperature (ΔTM) and GI_{50} values. Each biophysical property was plotted against each other for each of the three groups of MC's and the R^2 values were calculated from these graphs. In order to visualise trends in R^2 values we then tabulated them and colour coordinated them so that the highest values were bright yellow and the smallest values deep red. This enabled us to gain insights into relationships we otherwise would have missed. We have included these colour-coordinated tables here as well as the graphs directly mentioned in the paper.

Table S5. R^2 values of "BPY" group complexes showing correlation between size, lipophilicity, GI_{50} and change in melting temperature for C-MYC and H-TELO after 6 equivalents of MC. Colour indicates relative value where the highest values are bright yellow and the lowest deep red- colours evenly distributed between to accurately portray relative value.

	distance	volume	Lipophilicity	SA	Cmyc	H-telo	RA Cmyc	RA H-telo	HT29	U87	MCF-7	H460	A431	Du145	BE2-C	SJ-G2	MIA	MCF10A	ADDP
distance			0.9794		0.8776	0.7457	0.9886	0.427	0.3517	0.1816	0.0128	0.0165	0.0676	0.0392	8E-08	0.0133	0.2429	0.205	0.003
volume			0.9479		0.783	0.3883	0.93	0.553	0.7166	0.5294	0.666	0.0593	0.0123	0.0304	0.1345	0.0656	0.6038	0.0189	0.1215
Lipophilicity	0.9794	0.9479		0.9784	0.5707	0.6117	0.9986	0.7688	0.4936	0.3044	0.0009	0.0002	0.0141	0.003	0.0209	0.0008	0.3755	0.1297	0.0157
SA			0.9784		0.7116	0.4652	0.9662	0.6346	0.6392	0.4466	0.0314	0.0263	0.008	0.0085	0.0832	0.0307	0.5217	0.048	0.0728
Cmyc	0.8776	0.783	0.5707	0.7116					0.994	0.9279	0.4597	0.0005	0.0242	0.0086	0.5739	0.4576	0.9618	0.115	0.5547
H-telo	0.7457	0.3883	0.6117	0.4652					0.0112	0.0078	0.358	0.3732	0.5062	0.4427	0.2535	0.3607	0.002	0.7448	0.2706
RA Cmyc	0.9886	0.93	0.9986	0.9662					0.4563	0.2706	0.0005	0.4447	0.3148	0.3751	0.0116	0.00008	0.339	0.1558	0.0078
RA H-telo	0.427	0.553	0.7688	0.6346					0.075	0.0068	0.206	0.2183	0.338	0.2794	0.1218	0.2076	0.0247	0.5843	0.1348
HT29	0.3517	0.7166	0.4936	0.6392	0.994	0.0112	0.4563	0.075											
U87	0.1816	0.5294	0.3044	0.4466	0.9279	0.0078	0.2706	0.0068											
MCF-7	0.0128	0.666	0.0009	0.0314	0.4597	0.358	0.0005	0.206											
H460	0.0165	0.0593	0.0002	0.0263	0.0005	0.3732	0.4447	0.2183											
A431	0.0676	0.0123	0.0141	0.008	0.0242	0.5062	0.3148	0.338											
Du145	0.0392	0.0304	0.003	0.0085	0.0086	0.4427	0.3751	0.2794											
BE2-C	8E-08	0.1345	0.0209	0.0832	0.5739	0.2535	0.0116	0.1218											
SJ-G2	0.0133	0.0656	0.0008	0.0307	0.4576	0.3607	0.00008	0.2076											
MIA	0.2429	0.6038	0.3755	0.5217	0.9618	0.002	0.339	0.0247											
MCF10A	0.205	0.0189	0.1297	0.048	0.115	0.7448	0.1558	0.5843											
ADDP	0.003	0.1215	0.0157	0.0728	0.5547	0.2706	0.0078	0.1348											

Table S6. R^2 values of "PHEN" group complexes showing correlation between size, lipophilicity, GI_{50} and change in melting temperature for C-MYC and H-TELO after 6 equivalents of MC. Colour indicates relative value where the highest values are bright yellow and the lowest deep red- colours evenly distributed between to accurately portray relative value.

	distance	volume	Lipophilicity	SA	Cmyc	H-telo	RA Cmyc	RA H-telo	HT29	U87	MCF-7	H460	A431	Du145	BE2-C	SJ-G2	MIA	MCF10A	ADDP
distance			0.047		0.127	0.0348	0.0196	0.0266	1	0.9986	0.8622	0.9638	0.972	0.9868	0.9464	0.8322	1	0.9601	0.994
volume			0.092		0.6019	0.426	0.3798	0.118	0.75	0.7812	0.6796	0.8937	0.8789	0.8421	0.9183	0.9897	0.75	0.8995	0.8138
Lipophilicity	0.047	0.092		0.081	0.5707	0.6117	0.9986	0.7688	0.047	0.0326	0.0259	0.0007	0.0025	0.0107	0.002	0.0409	0.047	0.0003	0.0198
SA			0.081		0.5857	0.4097	0.3638	0.1075	0.756	0.7947	0.984	0.9037	0.8895	0.854	0.9272	0.9928	0.7642	0.9092	0.8265
Cmyc	0.127	0.6019	0.5707	0.5857					0.127	0.1526	0.4593	0.2785	0.2579	0.2127	0.3171	0.5011	0.127	0.2871	0.1828
H-telo	0.0348	0.426	0.6117	0.4097					0.0348	0.0495	0.2892	0.1369	0.1213	0.0887	0.1672	0.3278	0.0348	0.1435	0.0686
RA Cmyc	0.0196	0.3798	0.9986	0.3638					0.0196	0.0184	0.2475	0.1062	0.0923	0.0638	0.1336	0.2844	0.0196	0.0008	0.0467
RA H-telo	0.0266	0.118	0.7688	0.1075					0.029	0.0311	0.0423	0.0003	0.00002	0.0034	0.0037	0.0608	0.0296	0.1121	0.0091
HT29	1	0.75	0.047	0.756	0.127	0.0348	0.0196	0.029											
U87	0.9986	0.7812	0.0326	0.7947	0.1526	0.0495	0.0184	0.0311											
MCF-7	0.8622	0.6796	0.0259	0.984	0.4593	0.2892	0.2475	0.0423											
H460	0.9638	0.8937	0.0007	0.9037	0.2785	0.1369	0.1062	0.0003											
A431	0.972	0.8789	0.0025	0.8895	0.2579	0.1213	0.0923	0.00002											
Du145	0.9868	0.8421	0.0107	0.854	0.2127	0.0887	0.0638	0.0034											
BE2-C	0.9464	0.9183	0.002	0.9272	0.3171	0.1672	0.1336	0.0037											
SJ-G2	0.8322	0.9897	0.0409	0.9928	0.5011	0.3278	0.2844	0.0608											
MIA	1	0.75	0.047	0.7642	0.127	0.0348	0.0196	0.0296											
MCF10A	0.9601	0.8995	0.0003	0.9092	0.2871	0.1435	0.0008	0.1121											
ADDP	0.994	0.8138	0.0198	0.8265	0.1828	0.0686	0.0467	0.0091											

Table S7. R^2 values of "LARGE" group complexes showing correlation between size, lipophilicity, GI_{50} and change in melting temperature for C-MYC and H-TELO after 6 equivalents of MC. Colour indicates relative value where the highest values are bright yellow and the lowest deep red- colours evenly distributed between to accurately portray relative value.

	distance	volume	Lipophilicity	SA	Cmyc	H-telo	RA Cmyc	RA H-telo	HT29	U87	MCF-7	H460	A431	Du145	BE2-C	SJ-G2	MIA	MCF10A	ADDP
distance			0.0437		0.8556	0.3104	0.9583	0.9121	1.1153	0.0238	0.1884	0.0168	0.025	0.0145	0.0169	0.0229	0.0264	0.0022	0.0417
volume			0.7072		0.2974	0.8493	0.2144	0.0385	0.8218	0.6555	0.8929	0.6314	0.659	0.6225	0.6317	0.6526	0.6623	0.5504	0.7026
Lipophilicity	0.0437	0.7072		0.44699	0.5707	0.6117	0.9986	0.7688	0.9816	0.9969	0.9442	0.9935	0.9973	0.9919	0.9936	0.9966	0.9978	0.9736	1
SA			0.44699		0.5351	0.9762	0.4399	0.0021	0.4148	0.7023	0.605	0.3901	0.4184	0.3812	0.3904	0.4118	0.4228	0.3115	0.4649
Cmyc	0.8556	0.2974	0.5707	0.5351					0.0197	0.0025	0.058	0.0057	0.0022	0.0072	0.0057	0.0029	0.0018	0.0248	0.0001
H-telo	0.3104	0.8493	0.6117	0.9762					0.451	0.2686	0.5532	0.2466	0.2719	0.2387	0.2469	0.2659	0.2758	0.1793	0.3145
RA Cmyc	0.9583	0.2144	0.9986	0.4399					0.0021	0.0021	0.0217	0.029	0.0201	0.0321	0.0289	0.022	0.0018	0.0629	0.0091
RA H-telo	0.9121	0.0385	0.7688	0.0021					0.3503	0.5394	0.2564	0.5644	0.5357	0.2387	0.5641	0.5424	0.5313	0.1793	0.489
HT29	0.1153	0.8218	0.9816	0.4148	0.0197	0.451	0.0021	0.3503											
U87	0.0238	0.6555	0.9969	0.7023	0.0025	0.2686	0.0021	0.5394											
MCF-7	0.1884	0.8929	0.9442	0.605	0.058	0.5532	0.0217	0.2564											
H460	0.0168	0.6314	0.9935	0.3901	0.0057	0.2466	0.029	0.5644											
A431	0.025	0.659	0.9973	0.4184	0.0022	0.2719	0.0201	0.5357											
Du145	0.0145	0.6225	0.9919	0.3812	0.0072	0.2387	0.0321	0.2387											
BE2-C	0.0169	0.6317	0.9936	0.3904	0.0057	0.2469	0.0289	0.5641											
SJ-G2	0.0229	0.6526	0.9966	0.4118	0.0029	0.2659	0.022	0.5424											
MIA	0.0264	0.6623	0.9978	0.4228	0.0018	0.2758	0.0018	0.5313											
MCF10A	0.0022	0.5504	0.9736	0.3115	0.0248	0.1793	0.0629	0.1793											
ADDP	0.0417	0.7026	1	0.4649	0.0001	0.3145	0.0091	0.489											

GI_{50}

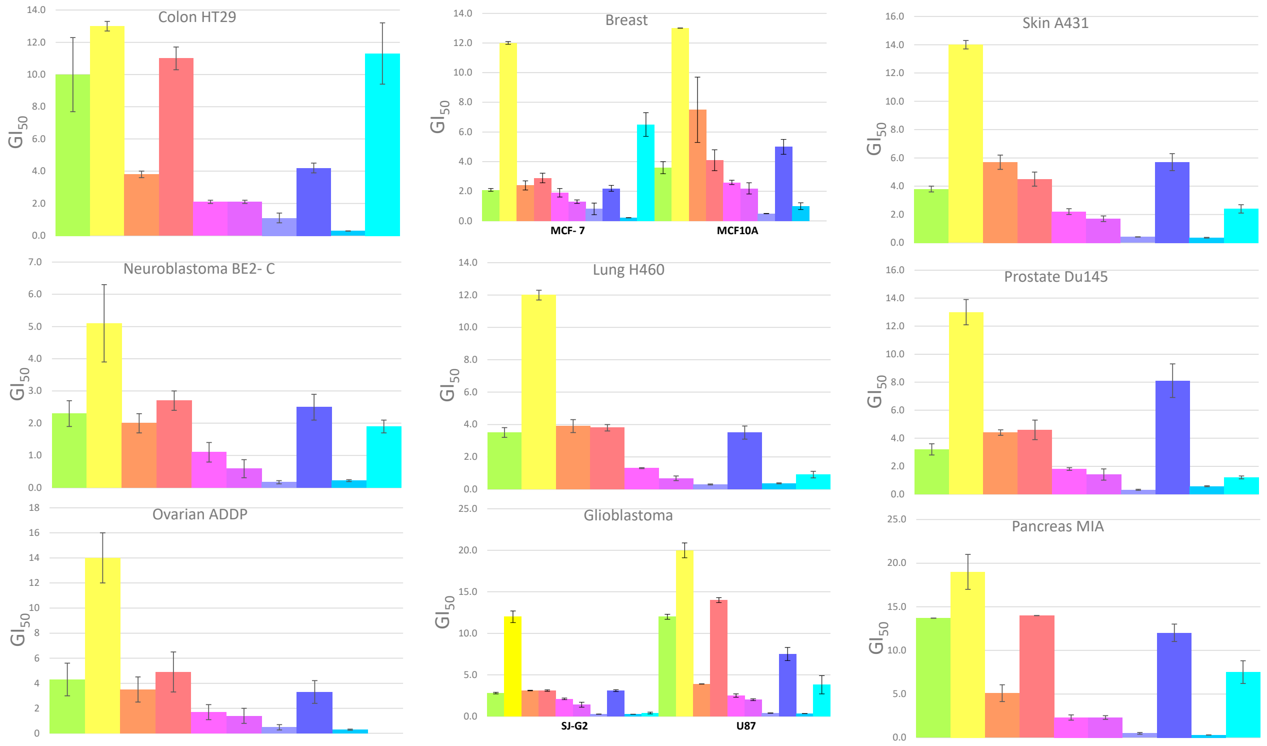

Figure S1. GI_{50} values of 1 $[Pt(Bequ)(BPY)]^+$, 2 $[Pt(Bequ)(44BPY)]^+$, 3 $[Pt(Bequ)(tertBPY)]^+$, 4 $[Pt(Bequ)(PHEN)]^+$, 5 $[Pt(Bequ)(5MePHEN)]^+$, 6 $[Pt(Bequ)(56Me_2PHEN)]^+$, 7 $[Pt(Bequ)(TMP)]^+$, 8 $[Pt(Bequ)(DPQ)]^+$, 9 $[Pt(Bequ)(BathoPHEN)]^+$, and ■ cisplatin in multiple cell lines. HT29 colon, U87 and SJ-G2 glioblastoma, H460 lung, A431 skin, BE2-C neuroblastoma, MIA pancreas, Du145 prostate, A2780 ovarian, MCF-7 breast and MCF10A breast (normal).

Table S1. A summary of the GI_{50} values of all nine complexes with errors

	**DOSE RESPONSE. GI50 = Concentration (µM) that inhibits cell growth by 50%**										
	(The lower the value the greater the growth inhibition)										
	**HT29**	**U87**	**MCF-7**	**H460**	**A431**	**Du145**	**BE2-C**	**SJ-G2**	**MIA**	**MCF10A**	**ADDP**
	Colon	*Glioblastoma*	*Breast*	*Lung*	*Skin*	*Prostate*	*Neuroblastoma*	*Glioblastoma*	*Pancreas*	*Breast (Normal)*	*Cis Res A2780 Ova*
	n=3	*n=3*	*n=3*	*n=3*	*n=3*	*n=3*	*n=3*	*n=3*	*n=3*	*n=3*	*n=3*
**PtBequBPY**	10 ± 2.3	12 ± 0.3	2.1 ±0.09	3.5 ± 0.3	3.8 ± 0.2	3.2 ± 0.4	2.3 ± 0.4	2.8 ± 0.1	13.7 ± 0	3.6 ± 0.4	4.3 ± 1.3
**PtBequ44BPY**	13 ± 0.3	20 ± 0.9	12 ± 0.3	12 ± 0.3	14 ± 0.3	13 ± 0.9	5.1 ± 1.2	12 ± 0.7	19 ± 2	13 ± 0	14 ± 2
**PtBequtertBPY**	3.8 ± 0.2	3.9 ± 0.0	2.4 ± 0.3	3.9 ± 0.4	5.7 ± 0.5	4.4 ± 0.2	2.0 ± 0.3	3.1 ±0.03	5.1 ±0.97	7.5 ± 2.2	3.5 ± 1.0
**PtBequPhen**	11 ± 0.7	14 ± 0.3	2.9 ±0.32	3.8 ± 0.2	4.5 ± 0.5	4.6 ± 0.7	2.7 ± 0.3	3.1 ±0.09	14 ± 0	4.1 ± 0.7	4.9 ± 1.6
**PtBequ5Phen**	2.1 ± 0.1	2.5 ± 0.2	1.9 ±0.29	1.3 ±0.03	2.2 ± 0.2	1.8 ± 0.1	1.1 ± 0.3	2.1 ± 0.1	2.3 ± 0.3	2.6 ±0.15	1.7 ± 0.6
**PtBequ56Phen**	2.1 ± 0.1	2.0 ± 0.1	1.3 ±0.12	0.67 ± 0.14	1.7 ± 0.2	1.4 ± 0.4	0.59 ± 0.28	1.4 ± 0.3	2.3 ± 0.2	2.2 ±0.38	1.4 ± 0.6
**PtBequTMP**	1.1 ± 0.3	0.38 ± 0.02	0.82 ± 0.39	0.29 ± 0.03	0.42 ± 0.01	0.32 ± 0.04	0.17 ± 0.05	0.27 ± 0.01	0.49 ± 0.10	0.50 ± 0.01	0.49 ± 0.21
**PtBequDPQ**	4.2 ± 0.3	7.5 ± 0.8	2.2 ± 0.2	3.5 ± 0.4	5.7 ± 0.6	8.1 ± 1.2	2.5 ± 0.4	3.1 ± 0.1	12 ± 1	5.0 ± 0.5	3.3 ± 0.9
**PtBequBatho**	0.29 ± 0.01	0.33 ± 0.03	0.22 ± 0.009	0.36 ± 0.03	0.36 ± 0.02	0.57 ± 0.04	0.22 ± 0.03	0.26 ± 0.00	0.30 ± 0.01	1.00 ± 0.23	0.30 ± 0.04
**Cisplatin**	11.3 ± 1.9	3.8 ± 1.1	6.5 ± 0.8	1 ± 0.1	0.9 ± 0.2	2.4 ± 0.3	1.2 ± 0.1	1.9 ± 0.2	0.4 ± 0.1		
